# Sulfation of Phenolic Acids: Chemoenzymatic vs. Chemical Synthesis

**DOI:** 10.3390/ijms232315171

**Published:** 2022-12-02

**Authors:** Viola Kolaříková, Katerina Brodsky, Lucie Petrásková, Helena Pelantová, Josef Cvačka, Libor Havlíček, Vladimír Křen, Kateřina Valentová

**Affiliations:** 1 Institute of Microbiology of the Czech Academy of Sciences, Vídeňská 1083, CZ 14220 Prague, Czech Republic; 2 Department of Biochemistry and Microbiology, University of Chemistry and Technology Prague, Technická 5, CZ 16628 Prague, Czech Republic; 3 Institute of Organic Chemistry and Biochemistry, Czech Academy of Sciences, Flemingovo nám. 2, CZ 16610 Prague, Czech Republic; 4 Institute of Experimental Botany of the Czech Academy of Sciences, Vídeňská 1083, CZ 14220 Prague, Czech Republic

**Keywords:** sulfation, phenolic acids, aryl sulfotransferase, flavonoid metabolites, biotransformation

## Abstract

Phenolic acids are known flavonoid metabolites, which typically undergo bioconjugation during phase II of biotransformation, forming sulfates, along with other conjugates. Sulfated derivatives of phenolic acids can be synthesized by two approaches: chemoenzymatically by 3′-phosphoadenosine-5′-phosphosulfate (PAPS)-dependent sulfotransferases or PAPS-independent aryl sulfotransferases such as those from *Desulfitobacterium hafniense*, or chemically using SO_3_ complexes. Both approaches were tested with six selected phenolic acids (2-hydroxyphenylacetic acid (2-HPA), 3-hydroxyphenylacetic acid (3-HPA), 4-hydroxyphenylacetic acid (4-HPA), 3,4-dihydroxyphenylacetic acid (DHPA), 3-(4-hydroxyphenyl)propionic acid (4-HPP), and 3,4-dihydroxyphenylpropionic acid (DHPP)) to create a library of sulfated metabolites of phenolic acids. The sulfates of 3-HPA, 4-HPA, 4-HPP, DHPA, and DHPP were all obtained by the methods of chemical synthesis. In contrast, the enzymatic sulfation of monohydroxyphenolic acids failed probably due to enzyme inhibition, whereas the same reaction was successful for dihydroxyphenolic acids (DHPA and DHPP). Special attention was also paid to the counterions of the sulfates, a topic often poorly reported in synthetic works. The products obtained will serve as authentic analytical standards in metabolic studies and to determine their biological activity.

## 1. Introduction

Flavonoids are natural polyphenolic compounds commonly present in the human diet [1] that have beneficial effects on health, such as lowering blood pressure [2,3] or reducing the risk of death from cardiovascular diseases [4,5]. These effects have previously been attributed to the antioxidant, vasodilator, or anticoagulant properties of flavonoids [6,7]. More recently, a modern approach of para-hormesis has been promoted, suggesting that flavonoids with their weak pro-oxidant activity stimulate cellular cytoprotective mechanisms based on the Nrf2/ARE pathway [8,9]. However, these theories usually do not consider the very low bioavailability of individual flavonoids (typically plasma concentrations of max. 1 μM are reached) and their complex metabolism [1,10,11]. Several studies have shown that polyphenols are degraded by intestinal microbiota and broken down into small-molecule phenols and phenolic acids. During phase II metabolism, these small phenolic catabolites are then bioconjugated to form sulfated, glucuronidated, and methylated metabolites [10,12], which may be present in plasma in higher concentrations [13]. Therefore, sulfates of phenolic acids represent an intriguing class of compounds, both as standards for studying the metabolism of various polyphenols and as compounds with potentially interesting biological properties.

Chemical or chemoenzymatic methods may be used in the preparation of sulfates, and the target sulfates are often obtained in the form of sodium, potassium, or (trialkyl)ammonium salts. A common problem in sulfate synthesis is the potential contamination with inorganic salts, which are difficult to detect and remove due to the good water solubility of sulfates and which can significantly interfere with biological or other tests [14]. Silica gel chromatography, which is commonly used for purification, is often impractical because of the high polarity of the desired products. A further complication is the identification of the sulfates. Since the presence of the sulfate moiety at the original hydroxy group cannot be directly detected by conventional NMR methods, a combination of several analyses (MS, comparison of NMR with the spectrum of the starting material, or derivatization–methylation) is required to confirm these structures.

In the chemical synthesis of phenol sulfates, SO_3_ complexes with a nitrogen base are usually used. The SO_3_-pyridine complex (SO_3_·pyridine) was previously used in the sulfation of various phenols and phenolic acids, such as benzoic acid, isovanillic acid, or 3,4-dihydroxyphenylacetic acid [15,16], while the SO_3_-*N*-triethylamine complex (SO_3_·NEt_3_) was used in the synthesis of 3,4-dihydroxybenzoic acid sulfates [17] and various sulfates of quercetin [18]. A new method using the SO_3_-*N*-tributylamine complex (SO_3_·NBu_3_) was recently developed and used in the synthesis of sodium 4-methoxyphenyl sulfate [19]. This method allows easier purification since the tributylammonium sulfate intermediates are soluble in organic solvents. The SO_3_-*N*-trimethyl amine complex (SO_3_·NMe_3_) [20] and the SO_3_-dimethylformamide complex (SO_3_·DMF) [21] were also used for the preparation of sulfates. For persulfated compounds, microwave-assisted reactions can be advantageous and significantly increase the yield [20].

Occasionally, chemical sulfation can be accompanied by the formation of benzenesulfonic acids, products of electrophilic substitution on the aromatic ring. Such a by-product was detected in the reaction of hydroxytyrosol acetate with the SO_3_·pyridine complex. It has been suggested that the undesirable sulfonation occurs as a subsequent degradation reaction of the target sulfates and can be prevented by carrying out the reaction at a lower temperature and neutralizing the reaction mixture during the workup [16].

An alternative to SO_3_ complexes is chlorosulfonic acid, which has been successfully used in the preparation of various phenyl sulfates, including *p*-coumaric acid sulfate [22] and sulfated flavonoids [23]. A modification of this method uses chlorosulfonic acid esters (e.g., chlorosulfuric acid 2,2,2-trichloroethyl ester) [24,25] or sulfuryl imidazolium salts [26], which allow milder conditions but require subsequent deprotection of the sulfate group. Sulfur (VI) fluoride exchange (SuFEx) reaction [27] is another modern sulfation method, in which aryl fluorosulfates react with silyl-ethers to generate sulfuric acid diesters, which are subsequently reduced by hydrogenolysis to the target sulfates. However, this method would almost certainly not tolerate the presence of the carboxylic moiety.

Enzymatic reactions are often much more selective than typical chemical reagents. Significant progress has been made in the field of enzymatic sulfation in the last two decades. In nature, enzymatic sulfation usually involves sulfotransferases (SULT), with 3′-phosphoadenosine-5′-phosphosulfate (PAPS) as the sulfate donor. However, this method is unsuitable for preparative sulfation due to the high cost and lability of PAPS [28]. Current research instead employs PAPS-independent bacterial aryl sulfotransferases (AST), e.g., aryl sulfotransferase from *Desulfitobacterium hafniense*, in combination with a sulfate donor, such as *p*-nitrophenyl sulfate (*p*-NPS) [28], *N*-hydroxysuccinimide sulfate [29], or other sulfate donors [30]. These sulfations are relatively fast, proceed under mild conditions, tolerate many substrates, and can be used in preparative synthesis in the range of tens to hundreds of milligrams. Several ASTs have been successfully used for the sulfation of polyphenols, including flavonoids and flavonolignans [28,29,31,32].

**Scheme 1:**
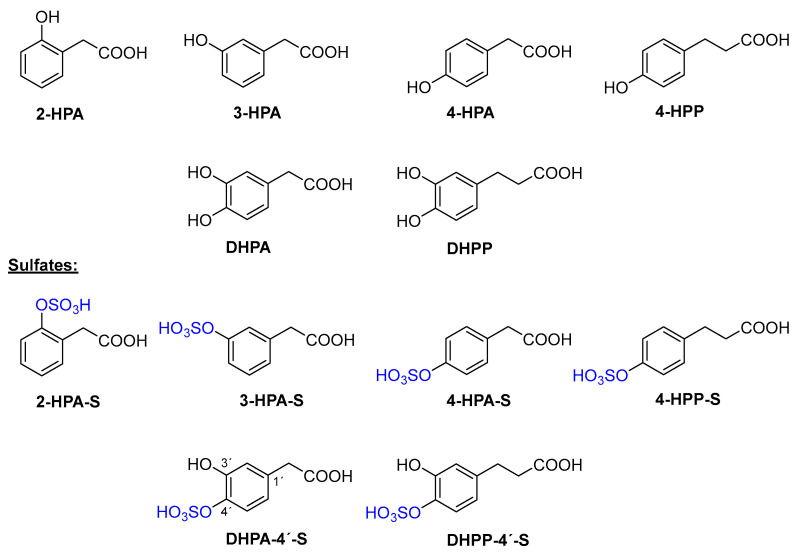
Phenolic acids selected for sulfation and their target sulfates; 2-hydroxyphenylacetic acid (**2-HPA**), 3-hydroxyphenylacetic acid (**3-HPA**), 4-hydroxyphenylacetic acid (**4-HPA**), 3,4-dihydroxyphenylacetic acid (**DHPA**), 3-(4-hydroxyphenyl)propionic acid (**4-HPP**), and 3,4-dihydroxyphenylpropionic acid (**DHPP**), 2-hydroxyphenylacetic acid 2-*O*-sulfate (**2-HPA-S**), 3-hydroxyphenylacetic acid 3-*O*-sulfate (**3-HPA-S**), 4-hydroxyphenylacetic acid 4-*O*-sulfate (**4-HPA-S**), 3,4-dihydroxyphenylacetic acid 4-*O*-sulfate (**DHPA-4-S**), 3-(4-hydroxyphenyl)propionic acid 4′-*O*-sulfate (**4-HPP-S**), and 3,4-dihydroxyphenylpropionic acid 4′-*O*-sulfate (**DHPP-4-S**).

In this work, we have focused on the sulfation of a series of mono- and dihydroxyphenolic acids (Scheme 1). We have synthesized a library of sulfates and compared the various methods of chemical and enzymatic sulfation. We also investigated the often-ignored counterions of these sulfates, which have previously been misattributed [33].

## 2. Results and Discussion

In the synthesis of phenolic acid sulfates, we explored the use of various sulfating agents: sulfur trioxide pyridine complex (SO_3_·pyridine), sulfur trioxide dimethyl formamide complex (SO_3_·DMF), chlorosulfuric acid, sulfur trioxide pyridine complex with subsequent addition of tributyl amine, chlorosulfuric acid-2,2,2-trichloroethyl ester. The workup and isolation method was carefully optimized to obtain the desired products. We also investigated the enzymatic sulfation of phenolic acids using the aryl sulfotransferase from *Desulfitobacterium hafniense* and *p*-NPS (*p*-nitrophenyl sulfate) as a sulfate donor.

### 2.1. Chemical Sulfation of Monohydroxyphenolic Acids

#### 2.1.1. Synthesis of Potassium Salts

**Scheme 2:**
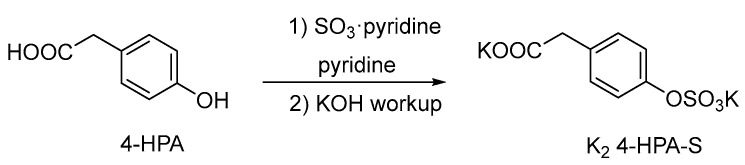
Chemical sulfation using SO_3_·pyridine in pyridine.

In 2018, Hartmann et al. reported the synthesis of 4-hydroxyphenylacetic acid sulfate (**4-HPA-S**), using a SO_3_·pyridine complex in pyridine [33]. We successfully replicated this method and obtained the same product with the corresponding properties and NMR. However, ^13^C NMR comparison with the parent 4-HPA showed a small shift in the carboxyl region and a significant shift in the CH_2_ group region (>6 ppm). Subsequent IR analysis revealed a significant shift of the C=O stretching peak, from 1686 cm^−1^ of 4-HPA to 1576 cm^−1^ of the obtained product, which is typical for the carbonyl vibration of carboxylates (COO^−^). With this knowledge, we reexamined the reaction and found that the reaction was followed by a strongly basic workup with potassium hydroxide. Elemental analysis, although not always reliable for sulfates or for potassium salts, showed the presence of both sulfur (9.1%) and potassium (20.1%), further confirming the presence of sulfate and tentatively suggesting the presence of two potassium atoms in the molecule. In view of all these data, we concluded that the compound misidentified as 4-hydroxyphenylacetic acid sulfate and reported in a free acid form [33] was in fact a potassium salt of the same compound, **K_2_ 4-HPA-S** (Scheme 2 and Table 1).

We subsequently tested this method with other phenolic acids. Furthermore, 3-hydroxyphenylacetic acid (3-HPA) and 4-hydroxyphenylpropionic acid (4-HPP) gave the expected products in reasonable yields; however, 2-hydroxyphenylacetic acid (2-HPA) was completely unreactive, possibly because of the steric hindrance of the OH group. In the cases where the target sulfate was obtained, it always contained a small amount of the potassium salt of the parent acid, which could not be removed (separation experiments with Sephadex LH-20 led to the decomposition of the sulfate); moreover, the presence of traces of inorganic salts could not be excluded. The results are summarized in Table 1.

#### 2.1.2. Synthesis of Sodium Salts

**Scheme 3:**
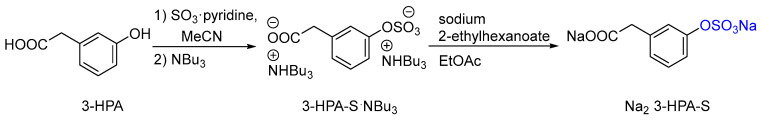
Chemical sulfation using SO_3_·pyridine via a tributylammonium intermediate.

The recently developed sulfation by tributylsulfoammonium betaine (TBSAB; SO_3_·NBu_3_) [19] has proven effective on a variety of phenolic substrates, including steroids [34] and phenolic acid salts and esters [35]. A modification of this method uses a SO_3_-pyridine complex, followed by the addition of tributylamine [36]. We successfully employed this method for the sulfation of several phenolic acids (Scheme 3 and Table 2). The phenolic acids were reacted with SO_3_·pyridine in acetonitrile at 90 °C for several hours, followed by the addition of tributylamine. Purification by column chromatography gave crude tributylammonium salts (HPA-S·NBu_3_ or HPP-S·NBu_3_), which were later treated with sodium 2-ethylhexanoate in ethyl acetate to yield the desired sulfates as sodium salts (Na_2_ HPA-S or Na_2_ HPP-S). The principle of this method dwells in the different solubility of the various reagents involved: sodium 2-ethylhexanoate, as well as the intermediate tributylammonium salts, are well-soluble in ethyl acetate, while the target sodium salts are insoluble in most organic solvents and naturally precipitate from the solution (NaI, another sodium reagent reported previously [36], proved inefficient in this case). The target sulfates **Na_2_ 3-HPA-S**, **Na_2_ 4-HPA-S**, and **Na_2_ 4-HPP-S** were obtained in excellent purity; however, 2-HPA again proved resistant to sulfation.

#### 2.1.3. Other Attempted Chemical Methods

**Scheme 4:**
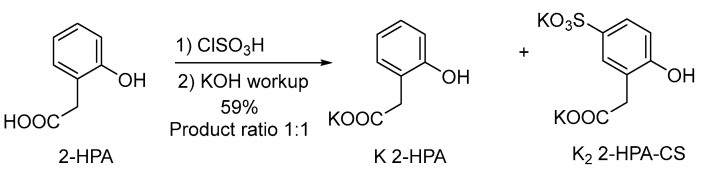
Sulfation of 2-HPA by chlorosulfuric acid.

We have investigated several other sulfation methods, but none of them has been particularly successful. We were especially interested in the synthesis of 2-hydroxyphenylacetic acid sulfate (2-HPA-S), but the steric hindrance of its parent 2-hydroxyphenylacetic acid proved very difficult because 2-HPA remained inert to most sulfating agents and conditions (SO_3_·DMF in DMF at RT/reflux, SO_3_·Et_3_N in dioxane, SO_3_·pyridine in pyridine or dioxane at RT/reflux, etc.). Finally, the reaction of 2-HPA with chlorosulfuric acid was carried out, followed by workup with KOH. We obtained an inseparable mixture of the potassium salt of the starting acid (K 2-HPA) and a product of sulfonation on the aromatic ring **K_2_ 2-HPA-CS** in a 1:1 ratio (Scheme 4). We attempted to improve this method by introducing a solvent or using NBu_3_ to give the product of sulfonation as tributylammonium salt for better purification, but without success. For other substrates, reaction with chlorosulfuric acid resulted in complex mixtures containing aromatic sulfonation products.

We also tested the sulfation by chlorosulfuric acid-2,2,2-trichloroethyl ester, as this method has previously been used in the synthesis of various phenolic sulfates [24]; however, this method failed for all available substrates and gave complex mixtures, most likely due to the presence of the free carboxyl group. Protection of the carboxyl group could make the reaction feasible, but the need for protection and subsequent deprotection makes this method inefficient, especially considering the problems associated with sulfate purification.

### 2.2. Chemical Sulfation of Dihydroxyphenolic Acids

**Scheme 5:**
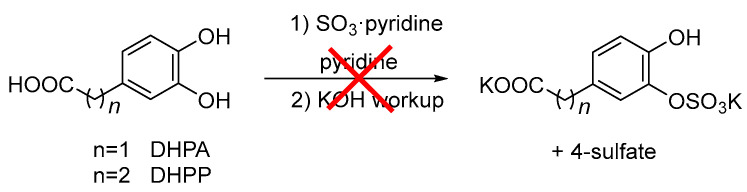
Attempted sulfation of dihydroxyphenolic acids.

Dihydroxyphenolic acids (DHPA and DHPP) showed remarkably different reactivity compared to their monohydroxyphenolic analogs. We first investigated their reaction with SO_3_·pyridine in pyridine; however, DHPA remained inert, whereas DHPP gave a complex, inseparable mixture of products, where the target product was not observed but benzenesulfonic acids were detected instead (Scheme 5). Similarly, the reaction of dihydroxyphenolic acids with SO_3_·pyridine at elevated temperature did not yield the target sulfates.

**Scheme 6:**
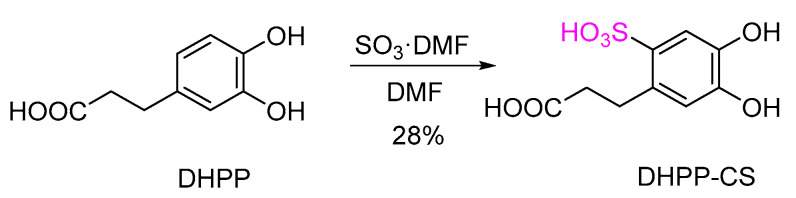
Synthesis of benzenesulfonic acid **DHPP-CS**.

We tested the reactivity of dihydroxyphenolic acids with SO_3_·DMF in DMF; however, none of the desired sulfates could be obtained. Instead, we observed the formation of benzenesulfonic acid **DHPP-CS** (Scheme 6), presumably due to *C*-sulfonation followed by *O*-desulfonation, as has previously been reported for other sulfates [16,37]. This process typically occurs at higher temperatures.

**Scheme 7:**
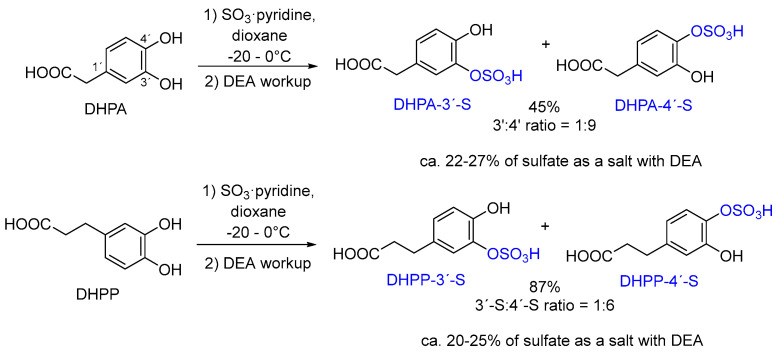
Synthesis of sulfates of dihydroxyphenolic acids **DHPA** and **DHPP**.

To prevent the formation of benzenesulfonic acids, we followed the procedure of Gomes et al. [16]. The reaction of DHPA with the SO_3_-pyridine complex in dioxane at low temperature (−20 °C), followed by workup with diethylamine (DEA), gave the target sulfate as a mixture of the 3′- and 4′-sulfate in a ratio of 1:9. Subsequently, we applied the same method to synthesize the sulfate of 3,4-dihydroxyphenylpropionic acid as a mixture of the 3′- and 4′-sulfate in a ratio of 1:6, which was determined by ^1^H NMR (Scheme 7). However, the target sulfates were not pure. They contained both the target sulfates as free acids and their diethylamine salts (20–27%), which could not be separated by column chromatography.

**Scheme 8:**
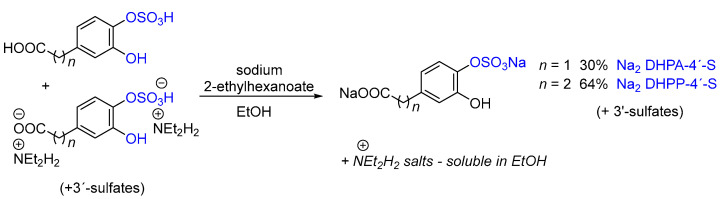
Synthesis of Na salts by reaction with sodium 2-ethylhexanoate.

To completely purify the products, we attempted to convert them to sodium salts using Dowex 50WX8 ion exchange resin column chromatography (eluent: water). The sulfates in the form of free acids were readily converted to sodium salts, however, the sulfates in the form of diethylamine salts remained as such and could not be removed from the mixture since they eluted with the sodium salts; moreover, prolonged exposure of the sulfates to these conditions led to their degradation. Therefore, the sulfates obtained were instead dissolved in ethanol and treated with sodium 2-ethyl hexanoate, upon which the sodium salts **Na_2_ DHPA-S** and **Na_2_ DHPP-S** precipitated from the solution. This is an extension of the method previously used in the synthesis of sodium sulfates of monohydroxyphenolic acids (Na_2_ HPA-S and Na_2_ HPP-S). This method worked relatively well for **Na_2_ DHPP-S** but gave poor yield in the case of **Na_2_ DHPA-S**, probably due to its partial solubility in ethanol (Scheme 8); however, the target sulfates were obtained in excellent purity and free of diethylammonium salts.

### 2.3. Enzymatic Sulfation of Phenolic Acids

**Scheme 9:**
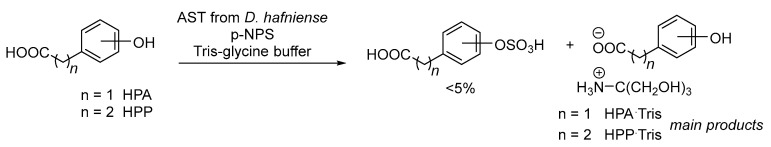
Attempted sulfation of monohydroxyphenolic acids using aryl sulfotransferase (AST) from *D. hafniense*.

Chemoenzymatic sulfation of phenolic acids was carried out based on our experience with the preparation of sulfated flavonoids [28,29,32], with adjustments in the purification and detection of sulfates. Initially, we tested the method for monohydroxyphenolic acids, which reacted with the aryl sulfotransferase from *D. hafniense* in a Tris-glycine buffer, with *p*-nitrophenolsulfate (*p*-NPS) acting as the sulfate donor. After purification by Sephadex LH-20 gel filtration column chromatography, we isolated the main products of the reactions. The presence of the sulfate moiety was detected by MS (ion [M-H]^+^); however, careful ^1^H and ^13^C NMR comparison with the parental acids showed strong shifts for carboxylic groups (Δ1.6–2.9 ppm in ^13^C NMR) and the neighboring α-CH_2_ group (Δ1.8–8.6 ppm in ^13^C NMR), while the majority of the shifts of the aromatic ring remained virtually unchanged. Furthermore, when compared with their parent acids, IR analysis of the new compounds showed significant shifts of the carbonyl groups (<1600 ppm), which is typical for carboxylates (COO^−^), while the presence of the SO_3_ group could not be confirmed. Therefore, we realized that the major products observed were the Tris salts of the parent phenolic acids, which were later also confirmed by their chemical synthesis, whereas the target sulfates were present only in trace amounts (up to 5%) (Scheme 9).

To limit the formation of Tris salts, we attempted the enzymatic sulfation of 3-hydroxyphenylacetic acid (3-HPA) in Britton–Robinson buffer (pH ~ 11), which allowed us to maintain the pH of the mixture at 8.5 (other buffers were not considered because the enzyme is the most active at pH 9.5 [30]). After the addition of the enzyme, the reaction turned yellow almost immediately, confirming the conversion of *p*-NPS to *p*-nitrophenol (*p*-NP). The blank experiment without the enzyme remained colorless throughout the reaction time (4 h). Another blank experiment under the same conditions but without the donor 3-HPA was colorless until we added *p*-NPS to the reaction, causing it to immediately turn yellow. Aliquots of the reaction and blank samples were taken every hour for HPLC analysis. Preliminary TLC analysis showed that little *p*-NP was present in the reaction mixture in all aliquots. HPLC analysis showed that only a small amount of *p*-NPS reacted and the enzyme was probably inhibited after a short time. The *p*-NP content did not change throughout the 4 h and no sulfated product was detected. We hypothesize that the enzyme bound to both substrates (donor and acceptor), cleaved the sulfate from *p*-NPS, and released *p*-NP into the reaction mixture but failed to complete the sulfate transfer to the target acid. The *p*-NP turned yellow in the alkaline reaction and gave a qualitative false positive result. The enzyme was bound to the acceptor acid and the sulfate moiety and remained in this state throughout the reaction.

**Scheme 10:**
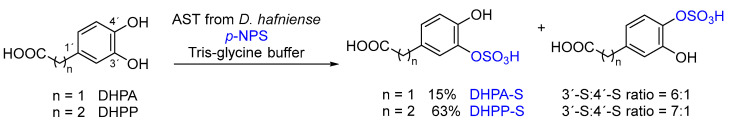
Enzymatic sulfation of dihydroxyphenolic acids.

In contrast, the reaction of the dihydroxyphenolic acids DHPA and DHPP with aryl sulfotransferase of *D. hafniense* and *p*-NPS gave the target products as mixtures of the 3′- and 4′-sulfates (Scheme 10). We hypothesize that the sulfate transfer was successful thanks to the two adjacent hydroxyl groups, allowing the enzyme to bind to one of the hydroxyl groups and realize sulfate transfer to the free hydroxyl group. In contrast to chemical sulfation, the 3′-sulfates were the major products of enzymatic sulfation (the ratios were determined by ^1^H NMR). In both cases, the compounds obtained contained tris(hydroxymethyl)aminomethane, which most likely forms salts with the sulfates obtained.

## 3. Materials and Methods

### 3.1. Chemicals and Reagents

2-Hydroxyphenylacetic acid (2-HPA), 3-hydroxyphenylacetic acid (3-HPA), 4-hydroxyphenylacetic acid (4-HPA), 3-(4-hydroxyphenyl)propionic acid (4-HPP), and *p*-nitrophenyl sulfate (*p*-NPS) were purchased from Sigma-Aldrich (Prague, Czech Republic). Furthermore, 3,4-dihydroxyphenylacetic acid (DHPA), pyridinium sulfate (48–50%) and SO_3_·DMF (47%) were purchased from Acros Organics (Thermo-Fisher Scientific, Waltham, MA, USA). Furthermore, 3,4-dihydroxyphenylpropionic acid (DHPP) was purchased from Carbosynth (Biosynth, Compton, UK). Common chemicals and solvents were purchased from Sigma-Aldrich, Lach-Ner (Neratovice, Czech Republic) or VWR chemicals (Stříbrná Skalice, Czech Republic).

Analytical TLC was performed on Al plates (silica gel 60 F_254_; Merck, Darmstadt, Germany) and visualized using UV light (254 nm).

### 3.2. HPLC

Data from the HPLC analyses were obtained using the Shimadzu Prominence System (Shimadzu, Kyoto, Japan), which consists of a mobile phase degasser (DGU-20A), two solvent delivery units (LC-20AD), a cooling autosampler (SIL-20AC), a column oven (CTO-10AS), a diode array detector (SPD-M20A), and a single quadrupole mass detector (LC-MS-2020) equipped with electrospray ionization. Data were collected and analyzed using Shimadzu LabSolutions software (ver. 5.75 SP2, Shimadzu Corporation, Tokyo, Japan) at a frequency of 40 Hz.

The reaction mixtures, purified fractions, and final products were analyzed using analytical HPLC. Separation of phenolic acids and their sulfates was performed using the temperature-controlled (45 °C) HPLC column Kinetex 5 µm PFP (pentafluorophenyl), 150 × 4.6 mm (Phenomenex, Torrance, CA, USA), with a guard column (5 × 4.6 mm; Merck, Germany) using linear gradient with the mobile phase ammonium acetate (10 mM), 0.1% HCOOH, pH 3.3 (phase A), and 100% methanol (phase B), 0 min 20% B, 0–20 min 20–50% B, 20–21 min 50–20% B, and 21–24 min 20% B to equilibrate of the column at a flow rate of 0.6 mL/min. The PDA detector data were recorded in the range of 200–450 nm and the signals of the absorption maximum of each compound were extracted.

### 3.3. LRMS

Low-resolution mass spectrometry (LRMS). Samples were dissolved in methanol/acetonitrile (1:1, methanol was added to improve the solubility of the samples) and injected (1 µL) into the mobile phase of acetonitrile (300 µL min^−1^) using a 50-µL loop. The values for spray, capillary, tube lens voltage, and capillary temperature were 3.5 kV, −16 V, −120 V, and 250 °C, respectively. A single quadrupole mass spectrometer (Shimadzu LC-MS-2020) equipped with electrospray ionization was used.

### 3.4. HRMS

Mass spectra were measured using an LTQ Orbitrap XL hybrid mass spectrometer (Thermo Fisher Scientific, Waltham, MA, USA) equipped with an electrospray ion source. The mobile phase was methanol/water (4:1, *v*/*v*) at a flow rate of 100 μL min^−1^. Samples were dissolved in methanol or methanol/water and injected into the mobile phase flow using a 5-μL loop. For the negative ion mode, spray voltage, capillary voltage, tube lens voltage, and capillary temperature were 5.0 kV, −25 V, −125 V, and 275 °C, respectively. For the positive ion mode, the spray voltage, capillary voltage, tube lens voltage, and capillary temperature were 5.0 kV, 9 V, 150 V, and 275 °C, respectively. The spectra were recorded with a resolution of 100,000.

### 3.5. IR

All IR analyses were carried out on a Nicolet iS5 spectrometer by the ATR method.

### 3.6. NMR

NMR data were acquired on Bruker Avance III 700 MHz (700.13 MHz for ^1^H, 176.05 MHz for ^13^C), Bruker Avance III 600 MHz (600.23 MHz for ^1^H, 150.93 MHz for ^13^C), and Bruker Avance III 400 MHz (399.87 MHz for ^1^H, 100.55 MHz for ^13^C) spectrometers in DMSO-*d*_6_ at 30 °C. The residual solvent signals were used as a reference (δ_H_ 2.499 ppm, δ_C_ 39.46 ppm). Standard ^1^H NMR, ^13^C NMR, ^1^H^13^C gHSQC, and ^1^H^13^C gHMBC experiments were performed using the manufacturer’s software TopSPin 3.5 (Bruker BioSpin, Rheinstetten, Germany). NMR structural analysis: Proton spin systems were assigned by COSY and then transferred to carbons by HSQC experiments. Quaternary carbons, singlets, and spin systems were put together using the HMBC experiment. Since hydroxyl groups resonated as very broad singlets that gave no correlations in the HMBC spectra, the positions of the sulfates were determined indirectly based on the comparison of the carbon chemical shifts with the parent sulfate acceptors. The sulfation was manifested by the upfield shift of the attached carbon atom together with the downfield shift of the two neighboring carbon atoms, as described by Purchartová et al. [28] for the catechol moiety. The formation of the salt was manifested in carbon spectra by the upfield shift of the carboxyl and the adjacent methylene carbon.

All analytical data (HPLS, ^1^H and ^13^C NMR, LCMS, HRMS, and IR) are shown in the Supplementary Materials.

### 3.7. Chemical Synthesis of Monohydroxyphenolic Acid Sulfates

#### 3.7.1. General Procedure A for the Synthesis of Potassium Salts

Furthermore, 3-HPA (500 mg, 3.29 mmol, 1 equiv.) and SO_3_·pyridine (46–48% purity, 1.07 g, 3.29 mmol, 1 equiv.) were suspended in dry pyridine (6 mL) and stirred for 3 days. After evaporation of pyridine, the residue was dissolved in as little water as possible, the pH of the mixture was adjusted to 6–7 with 25% KOH solution in water and the mixture was washed with EtOAc (3 × 2 mL). The solids formed were filtered off; the aqueous phase was evaporated and then dissolved in a small amount of water. The pH was adjusted to 10 with a 25% KOH solution and the mixture was then stirred at 60 °C for 1 h. After cooling, the pH of the mixture was adjusted to 7 with 20% sulfuric acid, the mixture was evaporated and then dissolved in as little water as possible (5 mL) at 40 °C. The addition of methanol (10 mL) caused the precipitation of a white powder (inorganic salts), which was filtered off, washed with methanol (5 mL), and then discarded while the combined liquid phases were allowed to stand overnight 0–4 °C. The newly formed precipitate (inorganic salts) was filtered off; the liquid phase was evaporated and then dissolved in as little methanol as possible (1 mL). The mixture was then sonicated, and the white precipitate was filtered off and discarded. Re-evaporation gave a white powder, which was again dissolved in as little methanol as possible and sonicated until a white precipitate was formed. Filtration gave the product **K_2_ 3-HPA-S** as a white solid (400 mg, 52%, contained 12% of K_2_ 3-HPA). For HPLC, ^1^H and ^13^C NMR, IR, and MS see Table S1 and Figures S1–S6 in the Supplementary Materials.

**K_2_ 4-HPA-S** was obtained following procedure A from 4-HPA (500 mg, 3.29 mmol, 1 equiv.), pyridine·SO_3_ (46–48% purity, 1.07 g, 3.29 mmol, 1 equiv.), and pyridine (6 mL) to give 4-HPA-S as a white powder (217 mg, 28%, contained 13% of K_2_ 4-HPA). For HPLC, ^1^H and ^13^C NMR, IR, and MS see Table S3 and Figures S13–S18 in the Supplementary Materials.

**K_2_ 4-HPP-S** was obtained by means of procedure A from 4-HPP (1.00 g, 6.02 mmol, 1 equiv.), pyridine·SO_3_ (46–48% purity, 1.95 g, 6.02 mmol, 1 equiv.), and pyridine (11 mL) to give 4-HPP-S as a white powder (670 mg, 45%, contained 13% of K_2_ 4-HPP). For HPLC, ^1^H and ^13^C NMR, IR, and MS see Table S5 and Figures S25–S30 in the Supplementary Materials.

#### 3.7.2. General Procedure B for the Synthesis of Sodium Salts

Moreover, 3-HPA (182 mg, 1.20 mmol, 1 equiv.) and SO_3_·pyridine (46–48% purity, 974 mg, 3.00 mmol, 2.5 equiv.) were suspended in dry acetonitrile (2 mL) under argon atmosphere, heated to 90 °C, and stirred for 4.5 h. Tributylamine (556 mg, 3.00 mmol, 2.5 equiv.) was then added to the mixture; the mixture was heated to 90 °C for another hour and then cooled to RT. After evaporation of the solvents, the residue was purified by column chromatography (eluent DCM:MeOH 3:1) to give the crude **3-HPA-S**·**NBu_3_**. The solid obtained was subsequently dissolved in ethanol (12 mL), sodium 2-ethylhexanoate (831 mg, 5.00 mmol, 4.17 equiv.) was added, and the mixture was stirred for 1.5 h at RT. The formed solid was then centrifuged and the liquid separated. The solid was again suspended in ethanol (10 mL) and centrifuged; this procedure was repeated three times. The combined liquid phases were then stirred overnight and then centrifuged again. The combined solids from all separations were washed with EtOAc (10 mL) and dried under vacuum to give the target **Na_2_ 3-HPA-S** as a white solid (77 mg, 23%, >99% purity). For HPLC, ^1^H and ^13^C NMR, IR, and MS see Table S2 and Figures S7–S12 in the Supplementary Materials.

**Na_2_ 4-HPA-S** was obtained according to procedure B. Starting from 4-HPA (152 mg, 1.00 mmol, 1 equiv.), SO_3_·pyridine (46–48% purity, 974 g, 3.00 mmol, 3 equiv.), and acetonitrile (2 mL) the mixture was stirred at 90 °C for 4 h, after which tributylamine (556 mg, 3 mmol, 3 equiv.) was added and the mixture was stirred for an additional hour. Crude **4-HPA-S**·**NBu_3_** was obtained by gradient column chromatography (eluent EtOAc:MeOH 40:1→10:1), dissolved in EtOH (12 mL), and reacted with sodium 2-ethylhexanoate (831 mg, 5.00 mmol, 5 equiv.) for 2.5 h. The mixture was then centrifuged, and the solids were suspended in EtOH (2 mL) and centrifuged again. The solids were then suspended in EtOAc (8 mL), centrifuged again, and dried under vacuum to afford the target **Na_2_ 4-HPA-S** as a white solid (147 mg, 53%, >99% purity). For HPLC, ^1^H and ^13^C NMR, IR, and MS see Table S4 and Figures S19–S24 in the Supplementary Materials.

**Na_2_ 4-HPP-S** was obtained following procedure B from 4-HPP (166 mg, 1.00 mmol, 1 equiv.), SO_3_·pyridine (46–48% purity, 974 g, 3.00 mmol, 3 equiv.), and acetonitrile (2 mL). The mixture was stirred at 90 °C for 7 h then tributylamine (556 mg, 3 mmol, 3 equiv.) was added and the mixture was stirred for another hour. Crude **4-HPP-S**·**NBu_3_** was obtained by column chromatography (eluent EtOAc:MeOH 25:1), dissolved in EtOH (12 mL), and reacted with sodium 2-ethylhexanoate (831 mg, 5.00 mmol, 5 equiv.) for 2.5 h. The mixture was then centrifuged, and the solids were suspended in EtOH (2 mL) and centrifuged again. The solids were then suspended in EtOAc (8 mL), centrifuged again, and dried under vacuum to afford the target **Na_2_ 4-HPP-S** as a white solid (45 mg, 16%, >99% purity). For HPLC, ^1^H and ^13^C NMR, IR, and MS see Table S6 and Figures S31–S36 in the Supplementary Materials.

#### 3.7.3. Sulfation of 2-HPA with Chlorosulfonic Acid

Chlorosulfonic acid (66.5 µL, 0.326 mmol, 1 equiv.) in DCM (0.28 mL) was added dropwise to 2-HPA (182 mg, 1.14 mmol, 3.5 equiv.), the mixture was briefly sonicated, and then stirred for 2 h. After evaporation of the solvent, the residue was dissolved in water (2 mL), the pH of the mixture was adjusted to 6–7 with 25% KOH solution in water, and the mixture was washed with EtOAc (3 × 2 mL). The aqueous phase was evaporated and then dissolved in a small amount of water, the pH was adjusted to 10 with 25% KOH solution and the mixture was then stirred at 60 °C for 1 h. After cooling, the pH of the mixture was adjusted to 7 with 20% sulfuric acid, the mixture was evaporated and then dissolved in as little water as possible (1.5 mL) at 40 °C. Addition of methanol (3 mL) resulted in the precipitation of a white powder (inorganic salts), which was filtered off, washed with methanol (2 mL), and then discarded, while the combined liquid phases were evaporated and dissolved in as little methanol as possible (1 mL). The mixture was then sonicated, and the white precipitate was filtered off and discarded. Re-evaporation produced a white powder, which was again dissolved in as little methanol as possible and precipitated overnight. After removing the liquid, the solid phase was dried under vacuum to give a mixture of **K 2-HPA** and **K_2_ 2-HPA-CS** in a 1:1 ratio as a white solid (45 mg, 59%). For HPLC, ^1^H and ^13^C NMR, IR, and MS see Tables S7 and S8 and Figures S37–S44 in the Supplementary Materials.

### 3.8. Chemical Synthesis of Dihydroxyphenolic Acid Sulfates

#### 3.8.1. General Procedure for the Synthesis of Dihydroxyphenolic Acid Sulfates

DHPA (500 mg, 2.97 mmol, 1 equiv.) and SO_3_·pyridine (46–48% purity, 1.93 g, 5.95 mmol, 2 equiv.) were dissolved in dry dioxane (10 mL) at 0 °C under argon atmosphere and stirred for 30 min. The mixture was then stored in a freezer (−20 °C) for 3 days. Water (25 mL) was then added and the reaction was neutralized with diethylamine. The mixture was washed with Et_2_O (2 × 50 mL), the aqueous phase was evaporated, and the residue was purified by column chromatography (eluent EtOAc:MeOH 9:2.5). The resulting yellow powder was lyophilized from *t*BuOH/water (10:1) to give a mixture of **DHPA-3-S** and **DHPA-4-S** in a 1:9 ratio (333 mg, 45%, ca. 10% of the products in the form of salts with diethylamine). For HPLC, ^1^H and ^13^C NMR, IR, and MS see Table S10 and Figures S45–S50 in the Supplementary Materials.

A mixture of **DHPP-3-S** and **DHPP-4-S** was prepared according to the general procedure above. Starting with DHPP (182 mg, 1.00 mmol, 1 equiv.) and SO_3_·pyridine (46–48% purity, 649 g, 2.00 mmol, 2 equiv.) in dioxane (3.4 mL), the mixture was stirred at 0 °C for 20 min and then kept at −20 °C for six days. After workup and purification by column chromatography (eluent EtOAc:MeOH 9:2.5), the target sulfates were obtained as a mixture of **DHPP-3-S** and **DHPP-4-S** in a 1:6 ratio (230 mg, 87%, ca. 10% of the products in the form of salts with diethylamine, pale yellow solid). For HPLC, ^1^H and ^13^C NMR, IR, and MS see Table S11 and Figures S51–S56 in the Supplementary Materials.

#### 3.8.2. Preparation of Sodium Salts of Dihydroxyphenolic Acids

A mixture of **DHPA-3-S** and **DHPA-4-S** (80 mg, 0.290 mmol, containing 10% of the salt with diethylamine) and sodium 2-ethylhexanoate (416 mg, 2.50 mmol, 8.6 equiv.) was dissolved in EtOH (3 mL) and stirred for 20 h to form a suspension. The mixture was then centrifuged, the liquid was removed, EtOAc (10 mL) was added to the solid, and the mixture was centrifuged again. The solid was dried under vacuum to give a mixture of **Na_2_ DHPA-3-S** and **Na_2_ DHPA-4-S** as a white powder (25 mg, 30%, >99% purity). For HPLC, ^1^H and ^13^C NMR, IR, and MS see Table S12 and Figures S57–S62 in the Supplementary Materials.

A mixture of **DHPP-3-S** and **DHPP-4-S** (82 mg, 0.279 mmol, containing 10% of the salt with diethylamine) and sodium 2-ethylhexanoate (332 mg, 2.00 mmol, 7.2 equiv.) was dissolved in EtOH (5 mL) and stirred for 5.5 h to form a suspension. The mixture was then centrifuged, the liquid was removed, EtOAc (10 mL) was added to the solid, and the mixture was centrifuged again. The solid was dried under vacuum to give a mixture of **Na_2_ DHPP-3-S** and **Na_2_ DHPP-4-S** as a white powder (55 mg, 64%, >99% purity). For HPLC, ^1^H and ^13^C NMR, IR, and MS see Table S13 and Figures S63–S68 in the Supplementary Materials.

#### 3.8.3. Preparation of Benzenesulfonic Acid DHPP-CS

DHPP (182 mg, 1.00 mmol, 1 equiv.) and SO_3_·DMF (47% purity, 1.30 g, 4.00 mmol, 4 equiv.) were dissolved in dry DMF (10 mL) and stirred overnight at RT. The solvent was then removed and the residue was loaded onto a Sephadex LH-20 column (GE Healthcare Bio-Sciences, Uppsala, Sweden; 30 g dry weight, 3 cm i.d.), which was packed and equilibrated in 80% methanol in water, eluting for 3 days, flow rate 0.15 mL/min, 25 °C, and 8 mL/fraction. After evaporation of solvents, **DHPP-CS** was obtained as a pale orange oil (71 mg, 27%). For HPLC, ^1^H and ^13^C NMR, IR and MS see Table S9 and Figures S40–S44 in the Supplementary Materials.

### 3.9. Chemoenzymatic Sulfation

#### 3.9.1. Preparation of Aryl Sulfotransferase from Desulfitobacterium Hafniense

Aryl sulfotransferase (AST) from *Desulfitobacterium hafniense* was heterologously expressed in *Escherichia coli* as described in our previous works [28,29]. Crude enzyme-containing cell lysate was used for the reactions.

#### 3.9.2. Preparation and Purification of Sulfates

The respective phenolic acid (HPA, DHPA, 4-HPP, or DHPP, 200 mg, 1 equiv.) was dissolved in 5 mL of acetone in a flask. Then, *p*-NPS in a Tris-glycine buffer was added to the solution (25 mg/mL, 1 equiv., 288 mg for HPA, 260 mg for DHPA, 264 mg for HPP, 241 mg for DHPP) containing 24 mL Tris-glycine buffer (100 mM, pH 8.9) and 1.5 mL AST enzyme (480 mU/mL of reaction mixture). The reaction mixture was then incubated in a shaking incubator at 30 °C for approximately 5 h under an inert atmosphere (Ar) using flask lids with septa. Aliquots of about 100 µL were taken to monitor reaction progress using TLC (mobile phase ethyl acetate/methanol/formic acid, 9/1/0.01; detection with UV light and iodine). After incubation, the reaction mixtures were heated to 95 °C and stored at −20 °C until purification.

For purification, the reaction mixture was partially evaporated in a rotary evaporator to remove acetone. The pH was then adjusted to 7.5–7.7 with formic acid. The reaction mixture was then extracted with ethyl acetate (3 × 50 mL) to remove *p*-nitrophenol (*p*-NP) from the reaction mixture (control by TLC; ethyl acetate/methanol/formic acid, 9/1/0.01). The remaining aqueous phase was completely evaporated, dissolved in 2–5 mL of 80% methanol in water, then centrifuged (5000 rpm, 20 min), and loaded onto a Sephadex LH-20 column (GE Healthcare Bio-Sciences, Uppsala, Sweden; 30 g dry weight, 3 cm i.d.), which was packed and equilibrated in 80% methanol in water, eluting for 2–3 days, flow rate 0.15 mL/min, 25 °C, and 2 mL/fraction. Fractions were analyzed by TLC (mobile phase ethyl acetate/methanol/formic acid, 9/1/0.01; detection with UV light and iodine). Fractions containing the product(s) were combined, evaporated in vacuo at 45 °C, then lyophilized, and stored at −20 °C until characterization by HPLC, MS, IR, and NMR.

**2-HPA·Tris** was obtained as a clear oil (267 mg, 74%). For HPLC, ^1^H and ^13^C NMR, IR, and MS see Table S14 and Figures S69–S72 in the Supplementary Materials.

**3-HPA·Tris** was obtained as a clear oil (265 mg, 74%). For HPLC, ^1^H and ^13^C NMR, IR, and MS see Table S15 and Figures S73–S76 in the Supplementary Materials.

**4-HPA·Tris** was obtained as a clear oil (339 mg, 94%). For HPLC, ^1^H and ^13^C NMR, IR, and MS see Table S16 and Figures S77–S80 in the Supplementary Materials.

**4-HPP·Tris** was obtained as a clear oil (139 mg, 40%). For HPLC, ^1^H and ^13^C NMR, IR, and MS see Table S17 and Figures S81–S84 in the Supplementary Materials.

A mixture of **DHPA-3′-S** and **DHPA-4′-S** was obtained as a white oil (45 mg, 15%). For ^1^H and ^13^C NMR, IR, and MS see Section 3.8.1

A mixture of **DHPA-3′-S** and **DHPA-4′-S** was obtained as a white oil (183 mg, 63%). For ^1^H and ^13^C NMR, IR, and MS see Section 3.8.1.

#### 3.9.3. Chemical Synthesis of Tris Salts of Phenolic Acids

The starting acid (46 mg for HPA or 50 mg for 4-HPP, 0.3 mmol) and tris(hydroxymethyl)aminomethan (37 mg, 0.3 mmol) were dissolved in water and stirred overnight at RT. After evaporation of solvents, the resulting salts **HPA·Tris** or **HPP·Tris** were obtained as clear oils in excellent purity (85 mg for HPA·Tris or 87 mg for HPP·Tris, clear oils, quant.). For ^1^H and ^13^C NMR, IR, and MS see Section 3.8.2.

## 4. Conclusions

We have investigated different approaches to the synthesis of sulfates of phenolic acids.

For monohydroxyphenolic acids, the best method in terms of product purity was the use of the SO_3_·pyridine complex in acetonitrile followed by the addition of tributylamine, giving tributylammonium intermediates that were later converted to sodium salts, with yields ranging between 16 and 53% (previously, only **Na_2_ 4-HPP-S** has been prepared in a 40% yield [38]). Sulfation with a SO_3_·pyridine complex followed by KOH workup gave similar yields (24–44%), but the products contained inseparable impurities (K^+^ salts of starting acids). Other chemical methods (other SO_3_ complexes, chlorosulfonic acid, chlorosulfuric acid-2,2,2-trichloroethyl ester) either did not give the desired sulfates or resulted in complex mixtures.

We have also discovered that some compounds previously reported as free acids [33] were actually in the form of salts. Therefore, we emphasize the need for a combination of analytical methods in the analysis of these compounds (^1^H and ^13^C NMR including comparison with parent compounds, IR, LCMS, and HRMS).

For the monohydroxyphenolic acids, enzymatic sulfation by AST from *D. hafniense* failed, possibly due to the inhibition of the enzyme, which bound to both substrates (phenolic acid and *p*-NPS) but failed to complete sulfate transfer to the target acid. Therefore, the major product(s) were salts of the parent acids with Tris, which was part of the buffer used.

In the case of dihydroxyphenolic acids, the typical conditions for chemical sulfation resulted in the significant formation of benzenesulfonic acids (products of C-sulfonation). Therefore, the sulfates were synthesized using the SO_3_·pyridine complex in dioxane at low temperature (−20 °C), which resulted in the formation of mixtures of 3- and 4-sulfates, with 4-sulfates as the major products (yields 30% and 64%). In contrast, enzymatic sulfation by AST from *D. hafniense* also gave mixtures of 3- and 4-sulfates, but 3-sulfates were the main products (yields 15% and 63%; previously, only enzymatic synthesis of DHPA-S was reported and the product was not isolated from the reaction mixture [39]). The sulfates obtained were purified by conversion to sodium salts.

The obtained products will serve as authentic analytical standards in metabolic studies and for determination of their biological activity.

## Figures and Tables

**Table 1:** Chemical sulfation by SO_3_·pyridine in pyridine.

Starting Acid	Product	Product Abbreviation	Yield [%]	Purity [%]	Side Products
2-HPA	No reaction	-	-	-	-
3-HPA	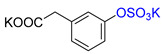	**K_2_ 3-HPA-S**	44	88	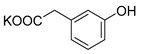
4-HPA	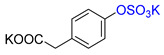	**K_2_ 4-HPA-S**	24	94	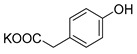
4-HPP	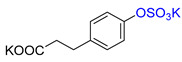	**K_2_ 4-HPP-S**	38	87	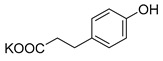

**Table 2:** Chemical sulfation using SO_3_·pyridine via a tributylammonium intermediate.

Starting Acid	Product	Product Abbreviation	Yield [%]	Purity [%]
2-HPA	No reaction	-	-	-
3-HPA	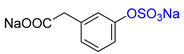	**Na_2_ 3-HPA-S**	23	>99
4-HPA	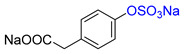	**Na_2_ 4-HPA-S**	53	>99
4-HPP	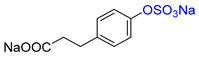	**Na_2_ 4-HPP-S**	16	>99

## Data Availability

The data presented in this study are available in the article or supplementary material.

## Supplementary Materials

The supporting information can be downloaded at: https://www.mdpi.com/article/10.3390/ijms232315171/s1.

## Abbreviations

ARE	Antioxidant responsive element
DCM	Dichloromethane
DEA	Diethylamine
DHPA	3,4-Dihydroxyphenylacetic acid
DHPP	3,4-Dihydroxyphenylpropionic acid
2-HPA	2-Hydroxyphenylacetic acid
3-HPA	3-Hydroxyphenylacetic acid
4-HPA	4-Hydroxyphenylacetic acid
4-HPP	3-(4-Hydroxyphenyl)propionic acid
p-NPS	p-Nitrophenyl sulfate
p-NP	p-Nitrophenol
Nrf2	NF-E2-related factor 2
Tris	Tris(hydroxymethyl)aminomethane
