# Supplementary material for: Sulfation of Phenolic Acids: Chemoenzymatic vs. Chemical Synthesis
Source: Int J Mol Sci. 2022 Dec 2;23(23):15171. doi: 10.3390/ijms232315171 (PMC9736156; doi:10.3390/ijms232315171)
Supplement: Supplementary file 1 [file ijms-23-15171-s001.zip › ijms-2037328-supplementary.pdf]

Supplementary Materials

Sulfation of Phenolic Acids: Chemoenzymatic vs. Chemical Synthesis

Viola Kolaříková[1], Katerina Brodsky[1,2], Lucie Petrásková[1], Helena Pelantová[1], Josef Cvačka[3], Libor Havlíček[4], Vladimír Křen[1] and Kateřina Valentová[1,*]

[1] Institute of Microbiology of the Czech Academy of Sciences, Vídeňská 1083, CZ 142 20 Prague, Czech Republic
[2] Department of Biochemistry and Microbiology, University of Chemistry and Technology Prague Technická 5, CZ 16628 Prague 6 (Czech Republic)
[3] Institute of Organic Chemistry and Biochemistry, Czech Academy of Sciences, Flemingovo nám. 2, CZ 166 10 Prague, Czech Republic
[4] Institute of Experimental Botany of the Czech Academy of Sciences, Vídeňská 1083, 14220 Prague, Czech Republic
* Correspondence: kata.valentova@email.cz

Contents

Potassium 2-(3-(sulfonatooxy)phenyl)acetate (K_2 3-HPA-S) **6**

Figure S1. HPLC chromatogram of **K_2 3-HPA-S** 6

Table S1. ^{1}H and ^{13}C NMR data for **K_2 3-HPA-S** 6

Figure S2. ^{1}H NMR spectrum of **K_2 3-HPA-S** 7

Figure S3. ^{13}C NMR spectrum of **K_2 3-HPA-S** 8

Figure S4. IR spectrum of **K_2 3-HPA-S.** 8

Figure S5. MS (ESI$^-$) spectrum of **K_2 3-HPA-S.** 9

Figure S6. HRMS (ESI$^-$) spectrum of **K_2 3-HPA-S.** 9

Sodium 2-(3-(sulfonatooxy)phenyl)acetate (Na_2 3-HPA-S) **9**

Figure S7. HPLC chromatogram of **Na_2 3-HPA-S** 10

Table S2. ^{1}H and ^{13}C NMR data for **Na_2 3-HPA-S** 10

Figure S8. ^{1}H NMR spectrum of **Na_2 3-HPA-S** 11

Figure S9. ^{13}C NMR spectrum of **Na_2 3-HPA-S** 11

Figure S10. IR spectrum of **Na_2 3-HPA-S.** 12

Figure S11. MS (ESI$^-$) spectrum of **Na_2 3-HPA-S**. 12

Figure S12. HRMS (ESI$^-$) spectrum of **Na_2 3-HPA-S.** 13

Potassium 2-(4-(sulfonatooxy)phenyl)acetate (K_2 4-HPA-S) **13**

Figure S13. HPLC chromatogram of **K_2 4-HPA-S** 13

Table S3. ^{1}H and ^{13}C NMR data for **K_2 4-HPA-S** 14

Figure S14. ^{1}H NMR spectrum of **K_2 4-HPA-S** 14

Figure S15. ^{13}C NMR spectrum of **K_2 4-HPA-S** 15

Figure S16. IR spectrum of **K_2 4-HPA-S**. 15

Figure S17. MS (ESI$^-$) spectrum of **K_2 4-HPA-S.** 16

Figure S18. HRMS (ESI$^-$) spectrum of **K_2 4-HPA-S.** 16

Sodium 2-(4-(sulfonatooxy)phenyl)acetate (Na_2 4-HPA-S) **16**

Figure S19. HPLC chromatogram of **Na_2 4-HPA-S** 17

Table S4. ^{1}H and ^{13}C NMR data for **Na_2 4-HPA-S** 17

Figure S20. ^{1}H NMR spectrum of **Na_2 4-HPA-S** 17

Figure S21. ^{13}C NMR spectrum of **Na_2 4-HPA-S** 18

Figure S22. IR spectrum of **Na_2 4-HPA-S.** 18

Figure S23. MS (ESI$^-$) spectrum of **Na_2 4-HPA-S.** 19

Figure S24. HRMS (ESI$^-$) spectrum of **Na_2 4-HPA-S.** 19

Potassium 3-(4-(sulfonatooxy)phenyl)propanoate (K_2 4-HPP-S) **19**

Figure S25. HPLC chromatogram of **K_2 4-HPP-S** 20

Table S5. ^{1}H and ^{13}C NMR data for **K_2 4-HPP-S**........20
Figure S26. ^{1}H NMR spectrum of **K_2 4-HPP-S**........21
Figure S27. ^{13}C NMR spectrum of **K_2 4-HPP-S**........21
Figure S28. IR spectrum of **K_2 4-HPP-S**.........22
Figure S29. MS (ESI$^-$) spectrum of **K_2 4-HPP-S**.........22
Figure S30. HRMS (ESI$^-$) spectrum of **K_2 4-HPP-S**.........23
Sodium 3-(4-(sulfonatooxy)phenyl)propanoate (Na_2 4-HPP-S)........23
Figure S31. HPLC chromatogram of **Na_2 4-HPP-S**........23
Table S6. ^{1}H and ^{13}C NMR data for **Na_2 4-HPP-S**........24
Figure S32. ^{1}H NMR spectrum of **Na_2 4-HPP-S**........24
Figure S33. ^{13}C NMR spectrum of **Na_2 4-HPP-S**........25
Figure S34. IR spectrum of **Na_2 4-HPP-S**.........25
Figure S35. MS (ESI$^-$) spectrum of **Na_2 4-HPP-S**.........26
Figure S36. HRMS (ESI$^-$) spectrum of **Na_2 4-HPP-S**.........26
Potassium 2-(2-hydroxyphenyl)acetate (K 2-HPA) and potassium 2-(2-hydroxy-5-sulfonatophenyl)acetate (K_2 2-HPA-CS)........26
Figure S37. HPLC chromatogram of **K 2-HPA** and **K_2 2-HPA-CS**........27
Table S7. ^{1}H and ^{13}C NMR data for **K 2-HPA**.........27
Table S8. ^{1}H and ^{13}C NMR data for **K_2 2-HPA-CS**.........28
Figure S38. ^{1}H NMR spectrum of **K 2-HPA** and **K_2 2-HPA-CS**.........28
Figure S39. ^{13}C NMR spectrum of **K 2-HPA** and **K_2 2-HPA-CS**.........29
3-(4,5-Dihydroxy-2-sulfophenyl)propanoic acid (DHPP-CS)........29
Figure S40. HPLC chromatogram of **DHPP-CS**........29
Table S9. ^{1}H and ^{13}C NMR data for **DHPP-CS**.........30
Figure S41. ^{1}H NMR spectrum of **DHPP-CS**.........30
Figure S42. ^{13}C NMR spectrum of **DHPP-CS**........31
Figure S43. MS (ESI$^-$) spectrum of **DHPP-CS**.........31
Figure S44. HRMS (ESI$^-$) spectrum of **DHPP-CS**.........32
3,4-dihydroxyphenylacetic acid 3-*O*-sulfate (DHPA-3'-S) and 3,4-dihydroxyphenylacetic acid 4-*O*-sulfate (DHPA-4'-S)........32
Figure S45. HPLC chromatogram of **DHPA-3'-S** and **DHPA-4'-S**........32
Table S10. ^{1}H and ^{13}C NMR data for **DHPA-3'-S** and **DHPA-4'-S**........33
Figure S46. ^{1}H NMR spectrum of **DHPA-3'-S and DHPA-4'-S**........34
Figure S47. ^{13}C NMR spectrum of **DHPA-3'-S and DHPA-4'-S**.........34
Figure S48. IR spectrum of **DHPA-3'-S** and **DHPA-4'-S**.........35
Figure S49. MS (ESI$^-$) spectrum of **DHPA-S**.........35

Figure S50. HRMS (ESI$^-$) spectrum of compound **DHPA-S.** 36

3,4-Dihydroxyphenylpropionic acid sulfates (DHPP-S): 3,4-dihydroxyphenylpropionic acid 3′-*O*-sulfate (DHPP-3′-S) and 3,4-dihydroxyphenylpropionic acid 4′-*O*-sulfate (DHPP-4′-S)........ 36

Figure S51. HPLC chromatogram of **DHPP-3′-S** and **DHPP-4′-S** 37

Table S11. ^{1}H and ^{13}C NMR data of **DHPP-3′-S** and **DHPP-4′-S** 37

Figure S52. ^{1}H NMR spectrum of **DHPP-3′-S** and **DHPP-4′-S**. 38

Figure S53. ^{13}C NMR spectrum of **DHPP-3′-S** and **DHPP-4′-S**. 39

Figure S54. IR spectrum of **DHPP-3′-S** and **DHPP-4′-S**. 39

Figure S55. MS (ESI$^-$) spectrum of **DHPP-S**. 40

Figure S56. HRMS (ESI$^-$) spectrum of **DHPP-S**. 40

Sodium dihydroxyphenylacetate sulfates (Na_2 DHPA-S): sodium 2-(4-hydroxy-3-(sulfonatooxy)phenyl)acetate (DHPA-3′-S) and sodium 2-(3-hydroxy-4-(sulfonatooxy)phenyl)acetate (DHPA-4′-S) 40

Figure S57. HPLC chromatogram of **Na_2 DHPA-3′-S** and **Na_2 DHPA-4′-S** 41

Table S12. ^{1}H and ^{13}C NMR data of **Na_2 DHPA-3′-S** and **Na_2 DHPA-4′-S** 41

Figure S58. ^{1}H NMR spectrum of **Na_2 DHPA-3′-S** and **Na_2 DHPA-4′-S**. 42

Figure S59. ^{13}C NMR spectrum of **Na_2 DHPA-3′-S** and **Na_2 DHPA-4′-S**. 43

Figure S60. IR spectrum of compound **Na_2 DHPA-3′-S** and **Na_2 DHPA-4′-S**. 43

Figure S61. MS (ESI$^-$) spectrum of **Na_2 DHPA-S**. 44

Figure S62. HRMS (ESI$^-$) spectrum of **Na_2 DHPA-S**. 44

Sodium phenylpropanoate sulfates (DHPP-S): sodium 3-(4-hydroxy-3-(sulfonatooxy)phenyl)propanoate (DHPP-3′-S) and sodium 2-(3-hydroxy-4-(sulfonatooxy)phenyl)propanoate (DHPP-4′-S)........ 45

Figure S63. HPLC chromatogram of **Na_2 DHPP-3′-S** and **Na_2 DHPP-4′-S** 45

Table S13. ^{1}H and ^{13}C NMR data of **Na_2 DHPP-3′-S** and **Na_2 DHPP-4′-S**........ 45

Figure S64. ^{1}H NMR spectrum of **Na_2 DHPP-3′-S and Na_2 DHPP-4′-S**........ 46

Figure S65. ^{13}C NMR spectrum of **Na_2 DHPP-3′-S and Na_2 DHPP-4′-S**........ 47

Figure S66. IR spectrum of compound **Na_2 DHPP-3′-S** and **Na_2 DHPP-4′-S**. 47

Figure S67. MS (ESI$^-$) spectrum of **Na_2 DHPP-S**. 48

Figure S68. HRMS (ESI$^-$) spectrum of **Na_2 DHPP-S**........ 48

(Trioxomethyl)methylammonium (2-hydroxyphenyl)acetate (2-HPA·Tris)........ 48

Figure S69. HPLC chromatogram of **2-HPA·Tris**........ 49

Table S14. ^{1}H and ^{13}C NMR data for compound **2-HPA·Tris**........ 49

Figure S70. ^{1}H NMR spectrum of **2-HPA·Tris**. 50

Figure S71. ^{13}C NMR spectrum of **2-HPA·Tris**. 50

Figure S72. IR spectrum of **2-HPA·Tris**........ 51

(Trioxomethyl)methylammonium (3-hydroxyphenyl)acetate (3-HPA·Tris)........ 51

Figure S73. HPLC chromatogram of **3-HPA·Tris**. 51

Table S15. ^{1}H and ^{13}C NMR data for **3-HPA·Tris**. 52

Figure S74. ^{1}H NMR spectrum of **3-HPA·Tris**. 52

Figure S75. ^{13}C NMR spectrum of **3-HPA·Tris**. 53

Figure S76. IR spectrum of **3-HPA·Tris**. 53

(Trioxomethyl)methylammonium (4-hydroxyphenyl)acetate (4-HPA·Tris) **54**

Figure S77. HPLC chromatogram of **4-HPA·Tris**. 54

Table S16. ^{1}H and ^{13}C NMR data for **4-HPA·Tris**. 54

Figure S78. ^{1}H NMR spectrum of **4-HPA·Tris**. 55

Figure S79. ^{13}C NMR spectrum of **4-HPA·Tris**. 55

Figure S80. IR spectrum of **4-HPA·Tris**. 56

(Trioxomethyl)methylammonium (4-hydroxyphenyl)propanoate (4-HPP·Tris) **56**

Figure S81. HPLC chromatogram of **4-HPA·Tris**. 56

Table S17. ^{1}H and ^{13}C NMR data for **4-HPP·Tris**. 57

Figure S82. ^{1}H NMR spectrum of **4-HPP·Tris**. 57

Figure S83. ^{13}C NMR spectrum of **4-HPP·Tris**. 58

Figure S84. IR spectrum of **4-HPP·Tris**. 58

Potassium 2-(3-(sulfonatooxy)phenyl)acetate (K_2 3-HPA-S)

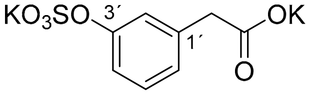

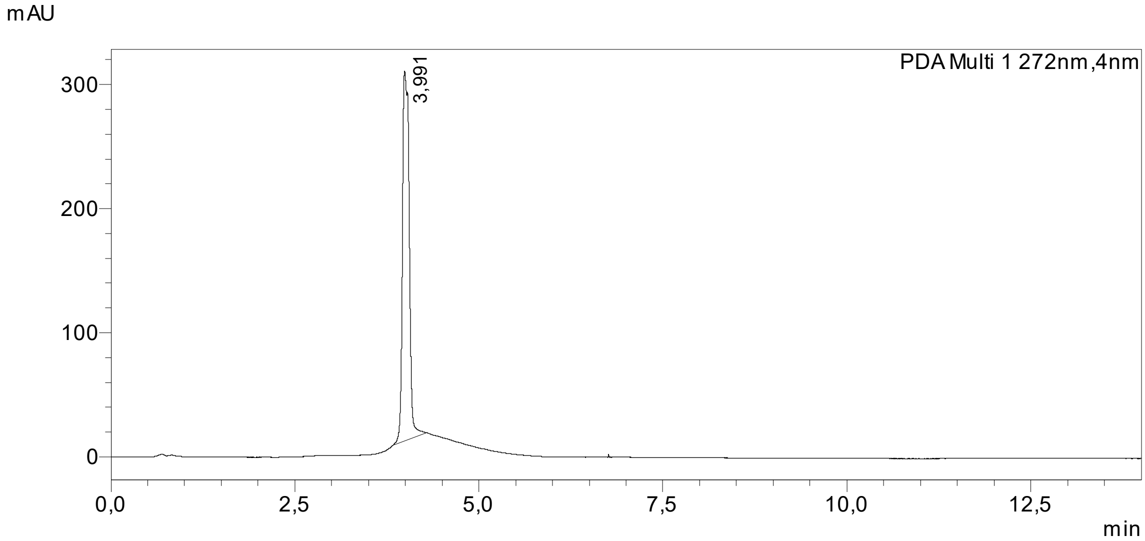

Figure S1. HPLC chromatogram of **K_2 3-HPA-S**

(RT= 3.991 min, 99% purity)

Table S1. ^{1}H and ^{13}C NMR data for **K_2 3-HPA-S**

(700.13 MHz for ^{1}H, 176.05 MHz for ^{13}C, DMSO-d_6)

Atom	δ_C	m.	δ_H	n_H	m.	*J* [Hz]	δ_C^{HPA}	$\delta_C - \delta_C^{HPA}$
**1**	173.95	S	-	0	-	-	172.52	1.43
**2**	45.29	T	3.243	2	s	-	40.72	4.57
**1′**	140.04	S	-	0	-	-	136.11	3.93
**2′**	121.45	D	7.084	1	dd	2.4, 1.5	116.12	5.33
**3′**	152.88	S	-	0	-	-	157.16	-4.28
**4′**	117.54	D	6.936	1	ddd	8.1, 2.4, 1.0	113.51	4.03
**5′**	127.73	D	7.101	1	dd	8.1, 7.5	129.08	-1.35
**6′**	124.16	D	6.908	1	ddd	7.5, 1.5, 1.0	119.83	4.33

Minority **K 3-HPA**:

Atom	δ_C	m.	δ_H	n_H	m.	*J* [Hz]	δ_C^{HPA}	$\delta_C - \delta_C^{HPA}$
**1**	174.10	S	-	0	-	-	172.52	1.58
**2**	45.19	T	3.191	2	s	-	40.72	4.47
**1'**	139.98	S	-	0	-	-	136.11	3.87
**2'**	116.32	D	6.726	1	dd	2.5, 1.4	116.12	0.2
**3'**	157.30	S	-	0	-	-	157.16	0.14
**4'**	112.32	D	6.525	1	ddd	8.0, 2.5, 0.9	113.51	-1.19
**5'**	128.23	D	6.963	1	dd	8.0, 7.5	129.08	-0.85
**6'**	119.49	D	6.584	1	ddd	7.5, 1.4, 0.9	119.83	-0.34

approximate molar ratio **K_2 3-HPA-S** : **K 3-HPA** = 88 : 12

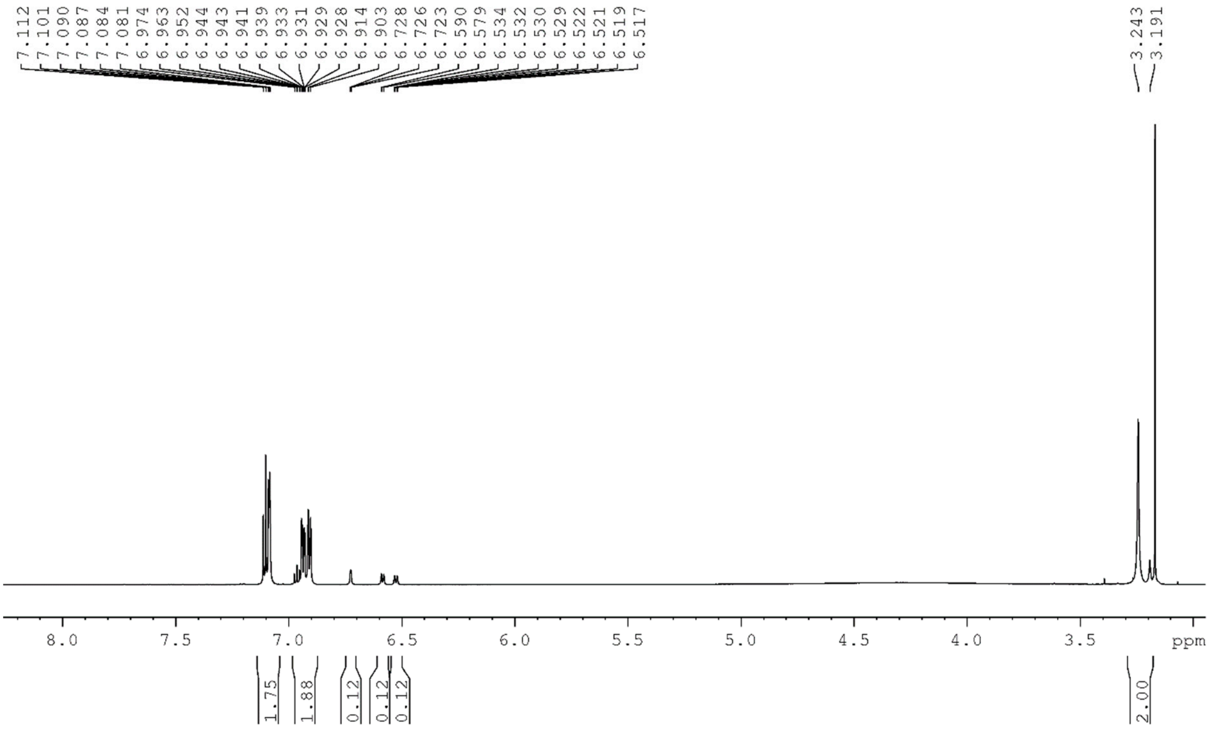

Figure S2. ^{1}H NMR spectrum of **K_2 3-HPA-S**

(700.13 MHz, DMSO-d_6)

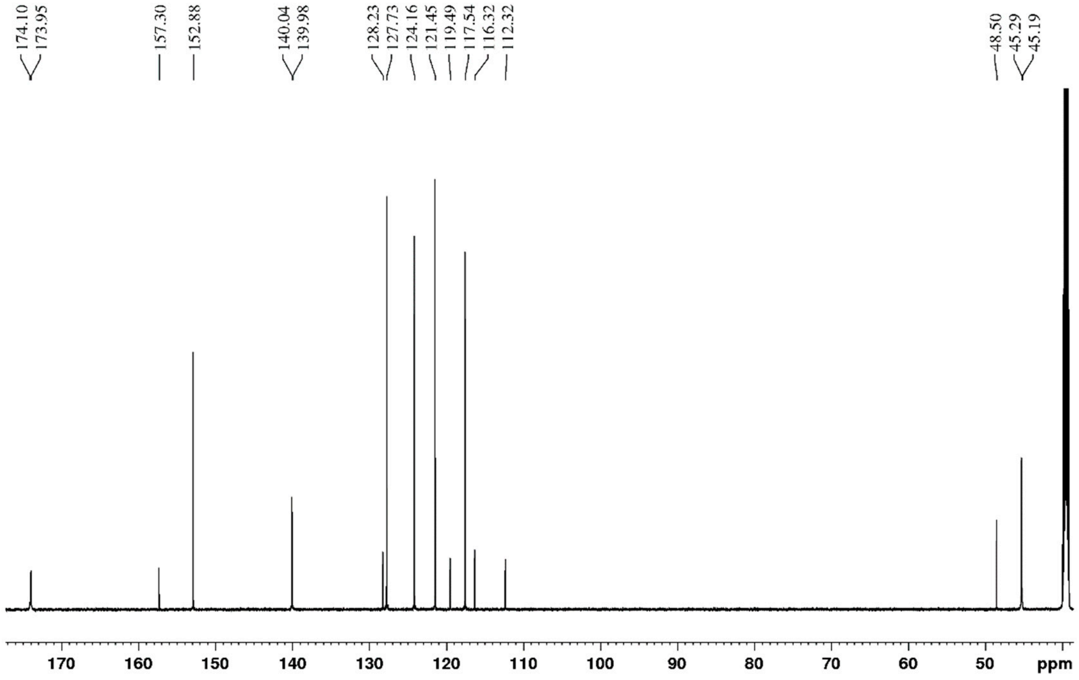

Figure S3. ^{13}C NMR spectrum of **K_2 3-HPA-S**
(176.05 MHz, DMSO-d_6)

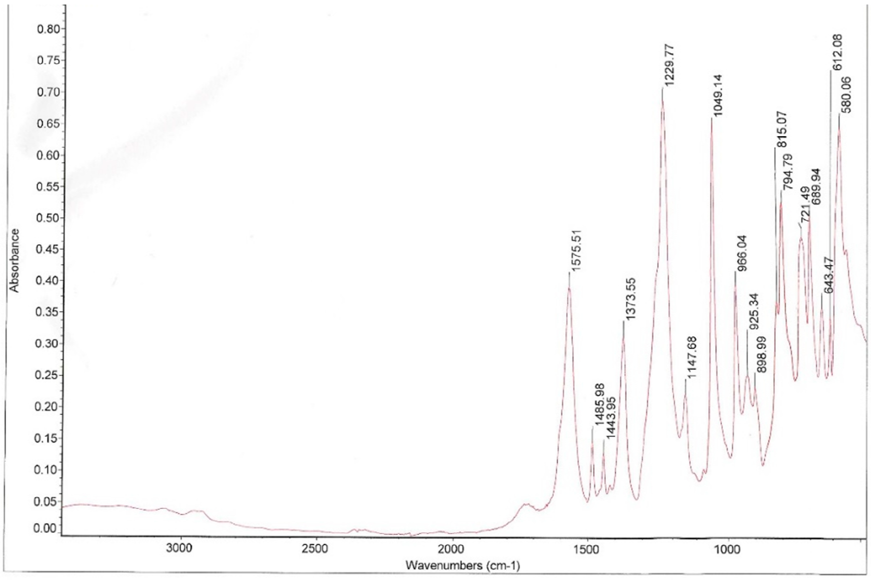

Figure S4. IR spectrum of **K_2 3-HPA-S.**

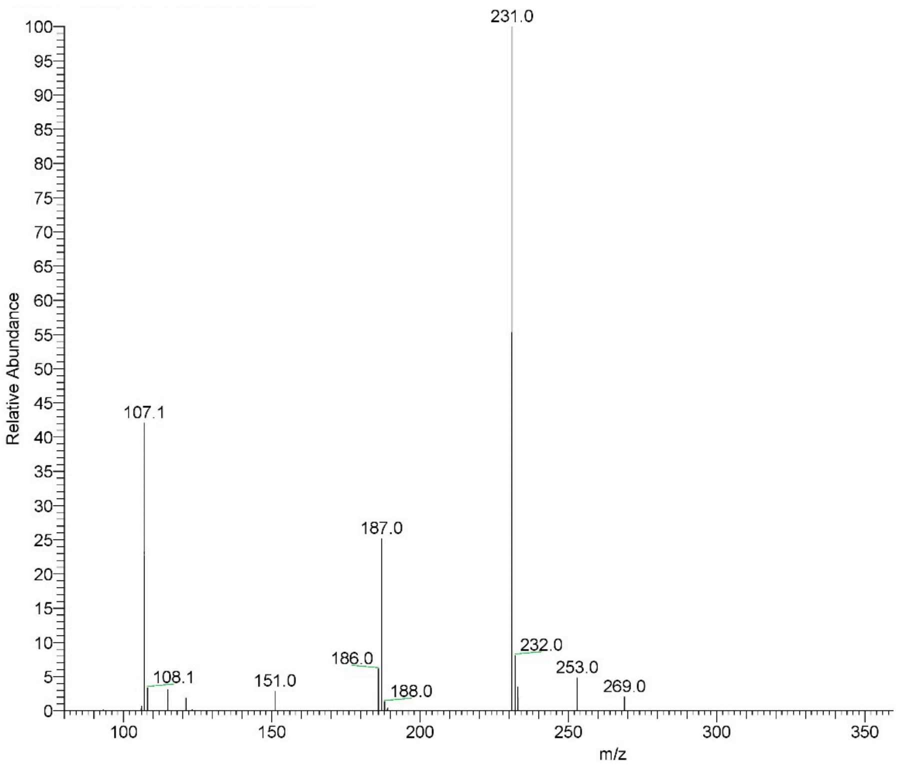

Figure S5. MS (ESI$^-$) spectrum of **K$_2$ 3-HPA-S.**

([M – 2K + H]$^-$, *m/z* 231.0).

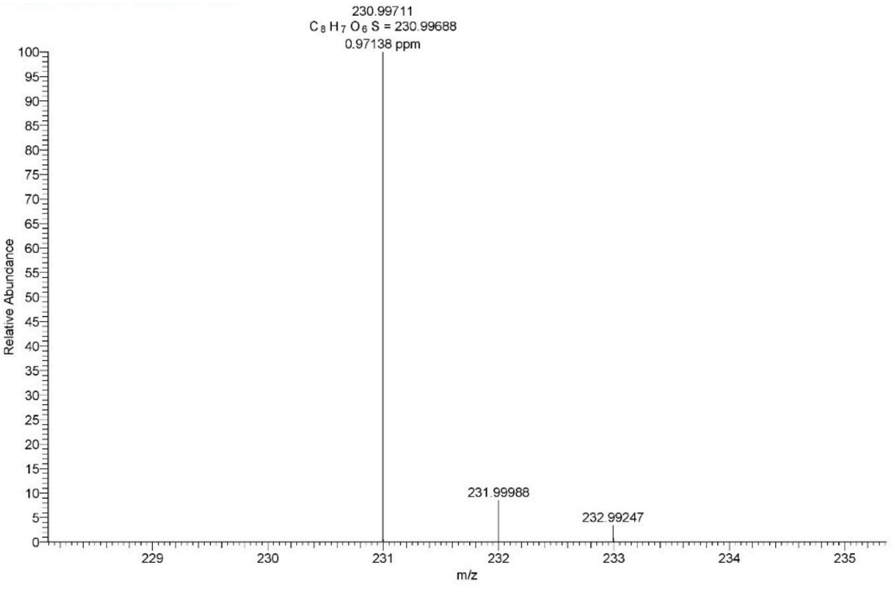

Figure S6. HRMS (ESI$^-$) spectrum of **K$_2$ 3-HPA-S.**

Calculated (for $C_8H_7O_6S^-$) 230.99688, measured 230.99711 (1.0 ppm).

Sodium 2-(3-(sulfonatooxy)phenyl)acetate (Na$_2$ 3-HPA-S)

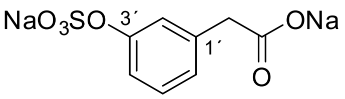

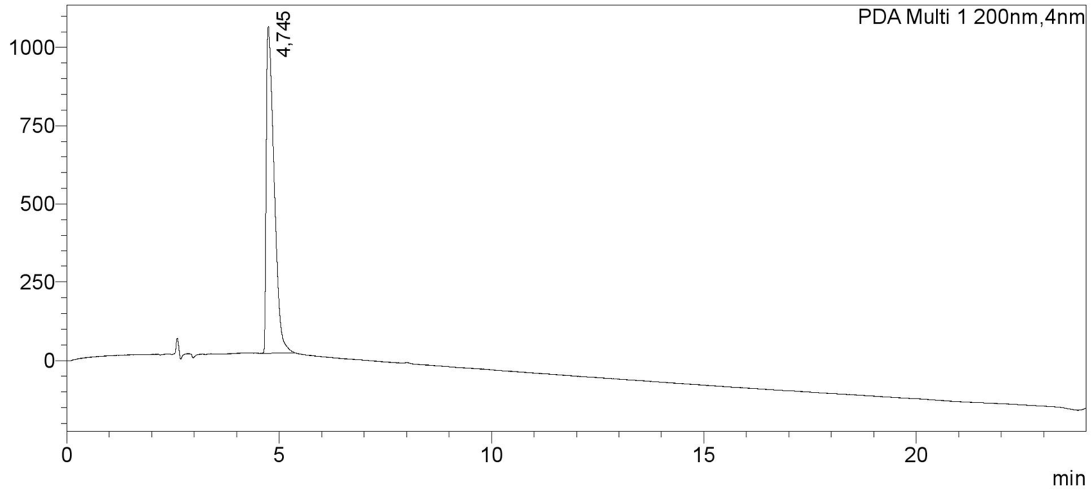

Figure S7. HPLC chromatogram of **Na_2 3-HPA-S**

(RT= 4.745 min, 99% purity)

Table S2. ^{1}H and ^{13}C NMR data for **Na_2 3-HPA-S**

(600.23 MHz for ^{1}H, 150.93 MHz for ^{13}C, DMSO-d_6)

Atom	δ_C	m.	δ_H	n_H	m.	J [Hz]	δ_C^{HPA}	δ_C - δ_C^{HPA}
**1**	174.97	S	-	0	-	-	172.52	2.45
**2**	45.76	T	3.214	2	s	-	40.72	5.04
**1’**	140.33	S	-	0	-	-	136.11	4.22
**2’**	121.44	D	7.048	1	dd	Σ*J* = 3.9	116.12	5.32
**3’**	152.82	S	-	0	-	-	157.16	-4.34
**4’**	117.30	D	6.945	1	ddd	8.1, 2.4, 1.1	113.51	3.79
**5’**	127.59	D	7.088	1	dd	Σ*J* = 15.6	129.08	-1.49
**6’**	124.08	D	6.931	1	ddd	7.5, Σ*J* = 2.5	119.83	4.25

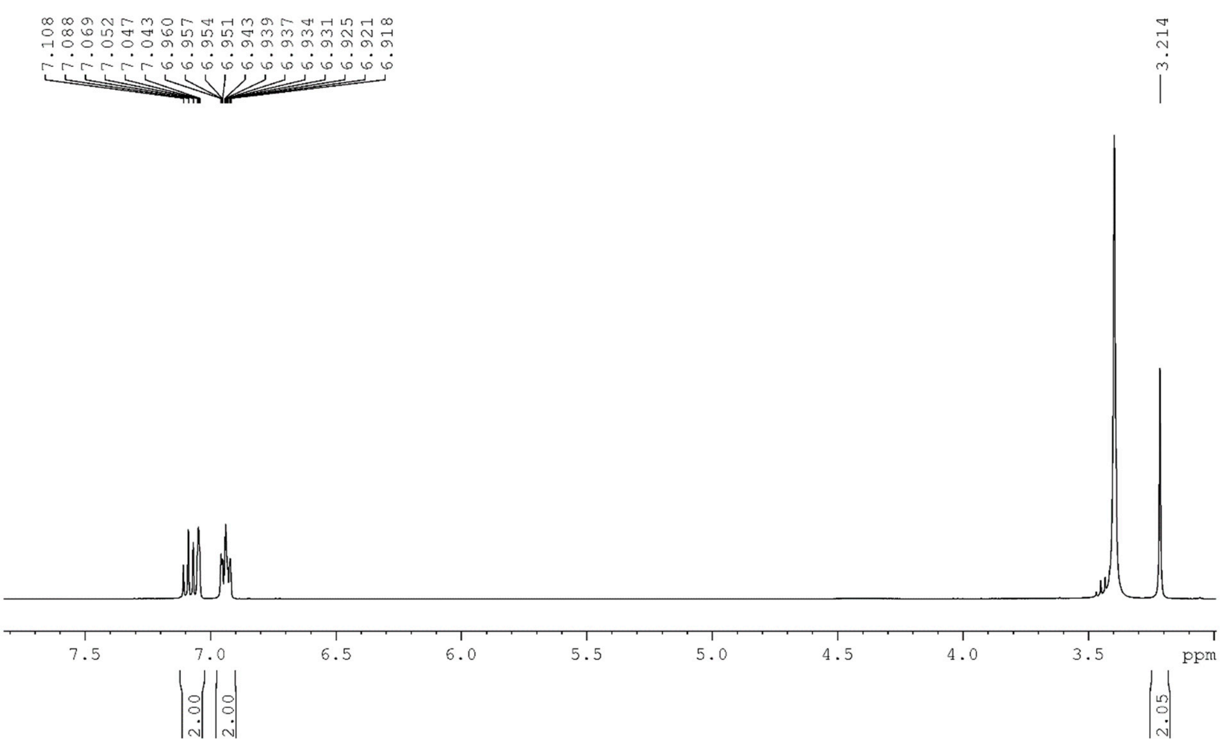

Figure S8. ^{1}H NMR spectrum of **Na2 3-HPA-S**

(600.23 MHz, DMSO-d_6)

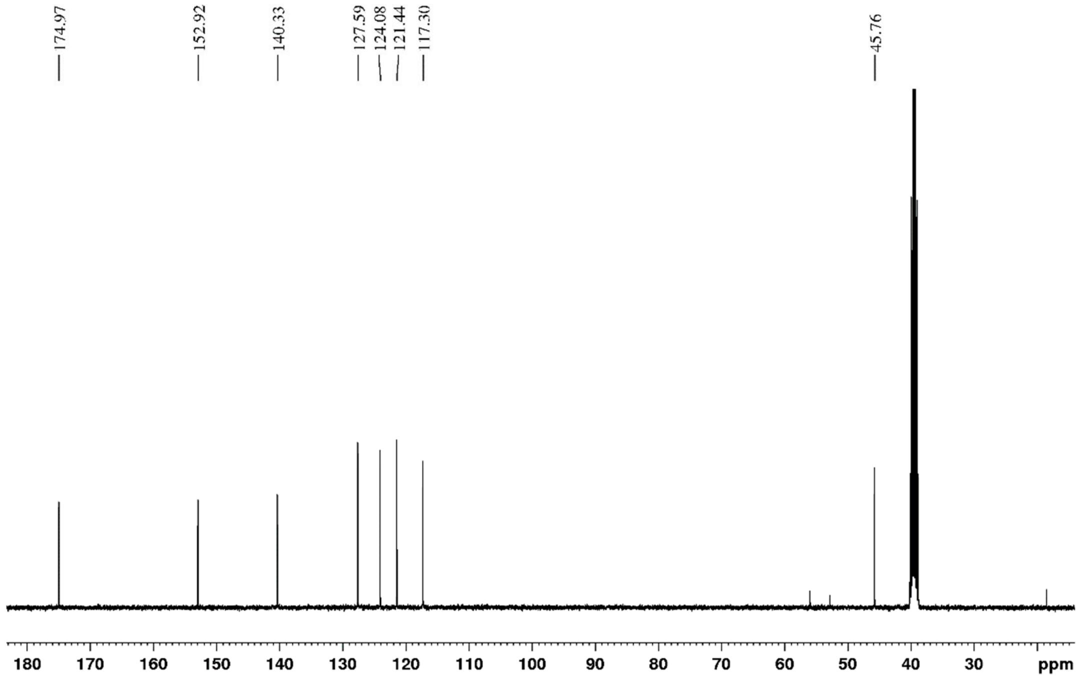

Figure S9. ^{13}C NMR spectrum of **Na2 3-HPA-S**

(150.93 MHz, DMSO-d_6)

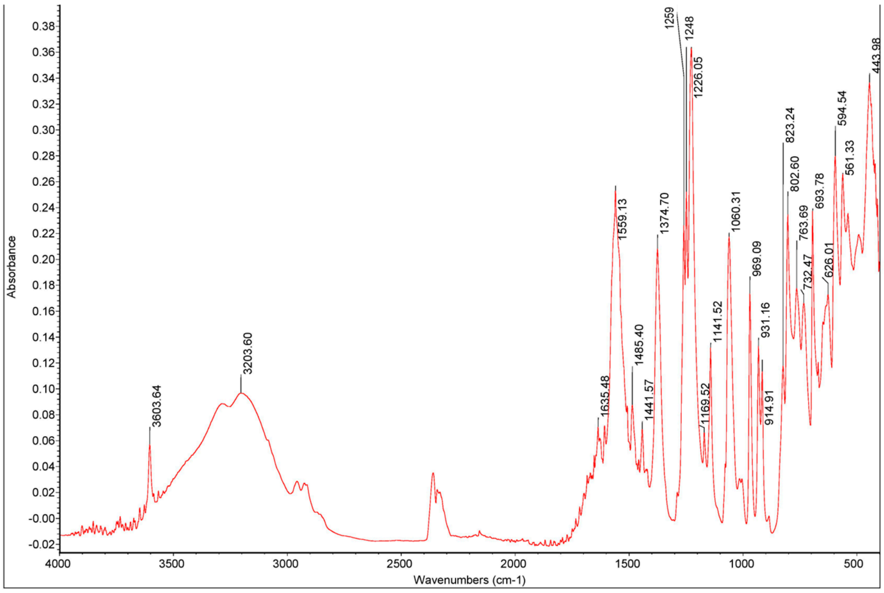

Figure S10. IR spectrum of **Na₂ 3-HPA-S.**

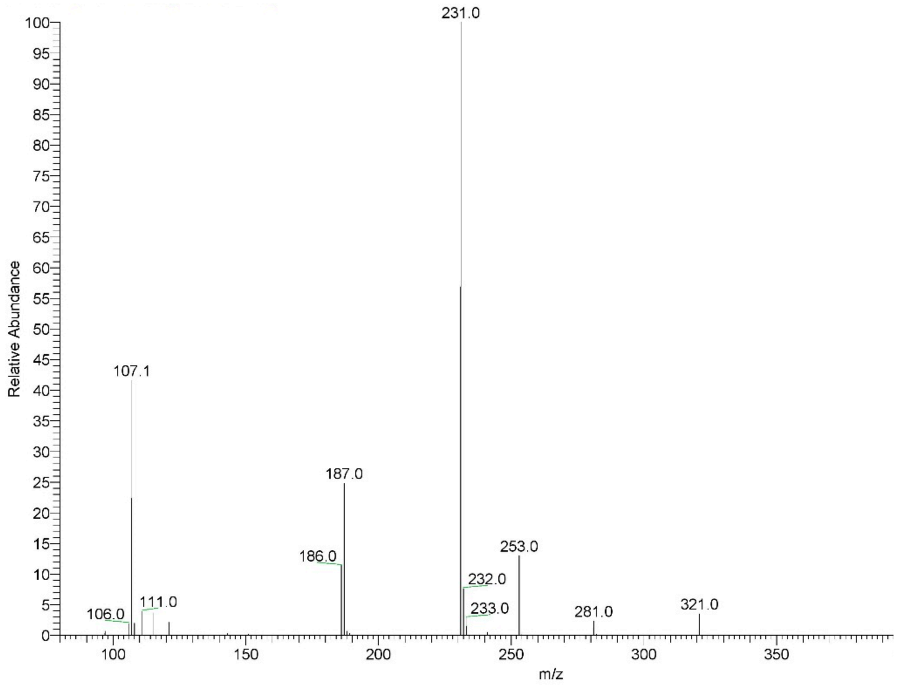

Figure S11. MS (ESI$^-$) spectrum of **Na$_2$ 3-HPA-S**.

([M − 2Na + H]$^-$, *m/z* 231.0; [M − Na]$^-$, *m/z* 253.0).

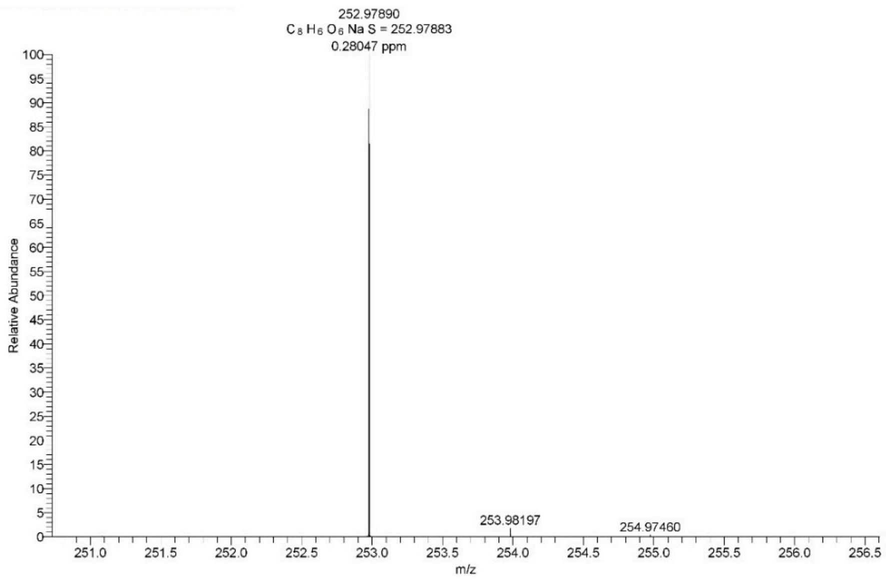

Figure S12. HRMS (ESI$^-$) spectrum of **Na$_2$ 3-HPA-S.**

Calculated (for $C_8H_6O_6NaS^-$) 252.97883, measured 252.97890 (0.3 ppm).

Potassium 2-(4-(sulfonatooxy)phenyl)acetate (K$_2$ 4-HPA-S)

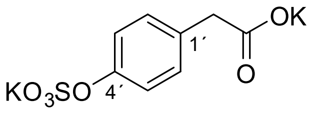

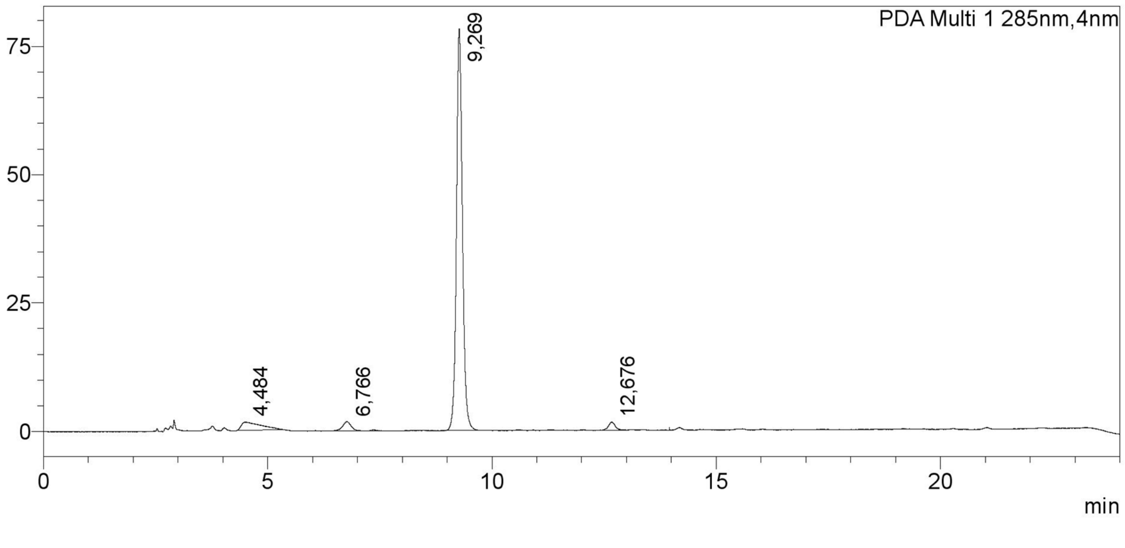

Figure S13. HPLC chromatogram of **K$_2$ 4-HPA-S**

(RT= 9.269 min, 89% purity)

Table S3. ^{1}H and ^{13}C NMR data for **K_2 4-HPA-S**

(399.83 MHz for ^{1}H, 100.54 MHz for ^{13}C, DMSO-d_6)

**Atom**	δ_C	**m.**	δ_H	n_H	m.	δ_C^{4HPA}	$\delta_C - \delta_C^{4HPA}$
**1**	173.96	S	-	0	-	172.99	0.97
**2**	45.86	T	3.131	2	s	39.79	6.07
**1’**	135.10	S	-	0	-	125.03	10.07
**2’**	129.13	D	7.086	2	m	130.13	-1.00
**3’**	119.80	D	6.982	2	m	114.92	4.88
**4’**	150.79	S	-	0	-	155.95	-5.16

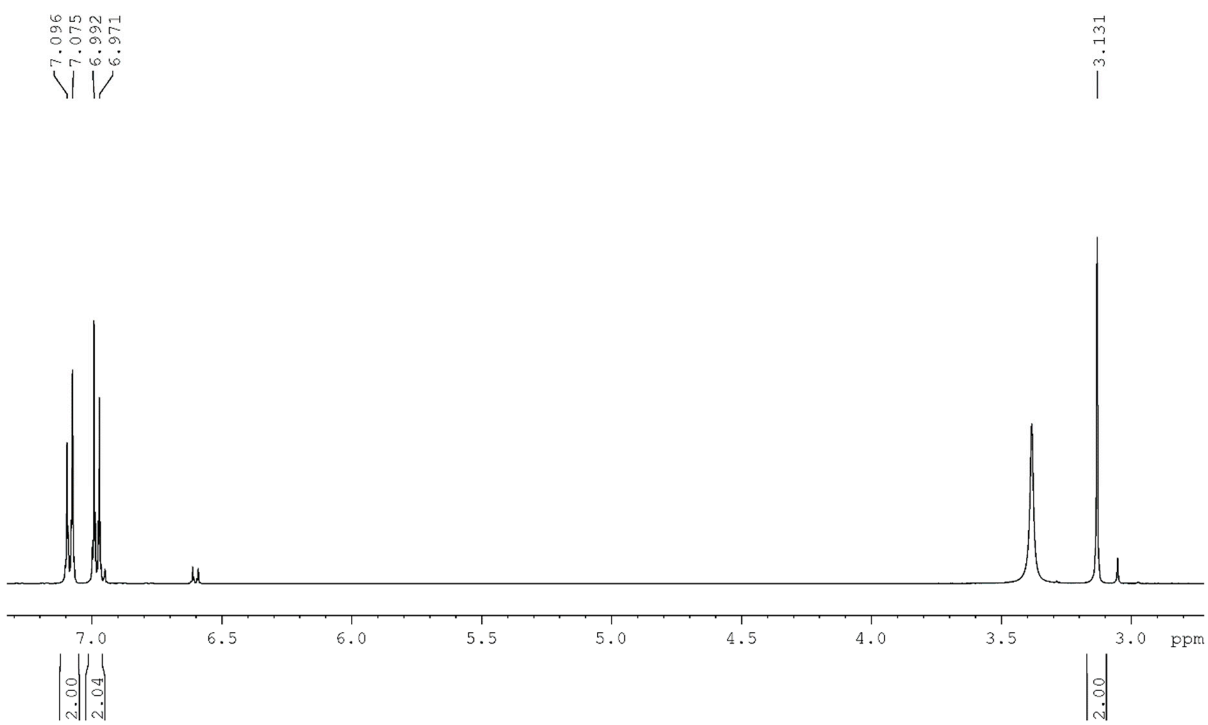

Figure S14. ^{1}H NMR spectrum of **K_2 4-HPA-S**

(399.83 MHz, DMSO-d_6)

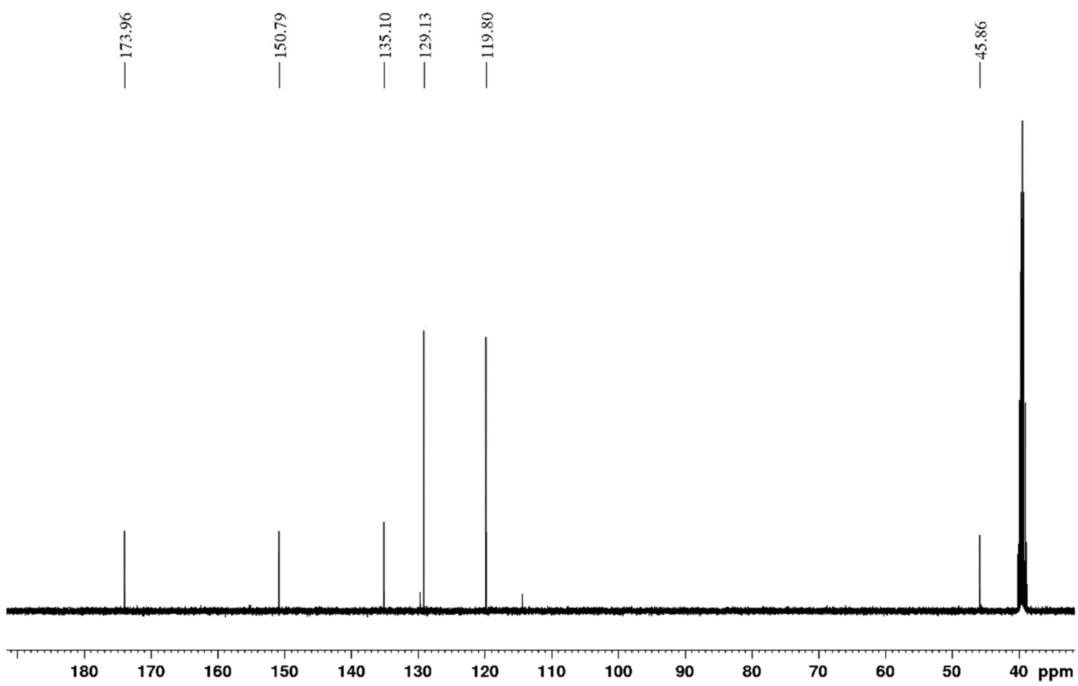

Figure S15. ^{13}C NMR spectrum of **K_2 4-HPA-S**

(100.54 MHz, DMSO-d_6)

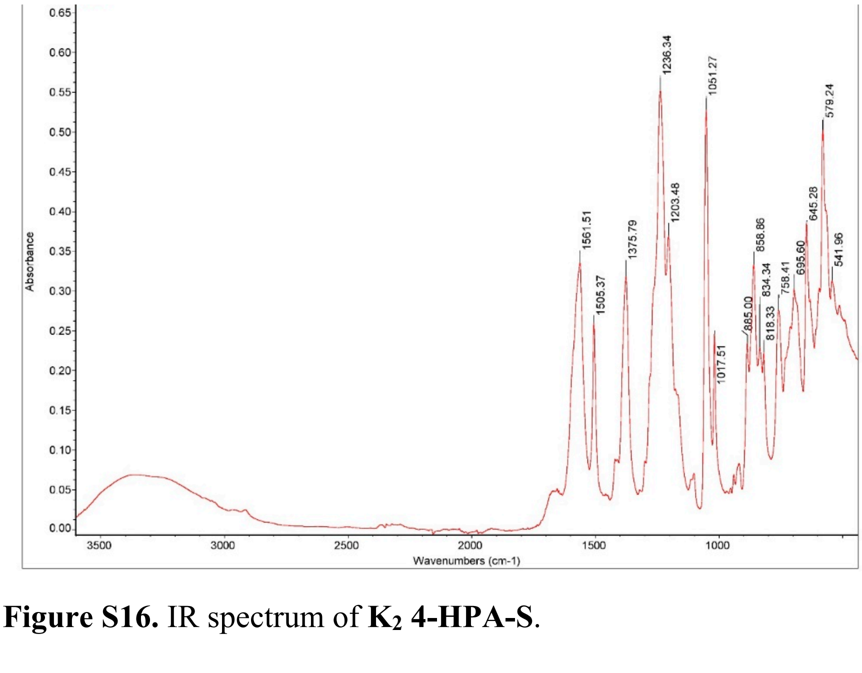

Figure S16. IR spectrum of **K_2 4-HPA-S**.

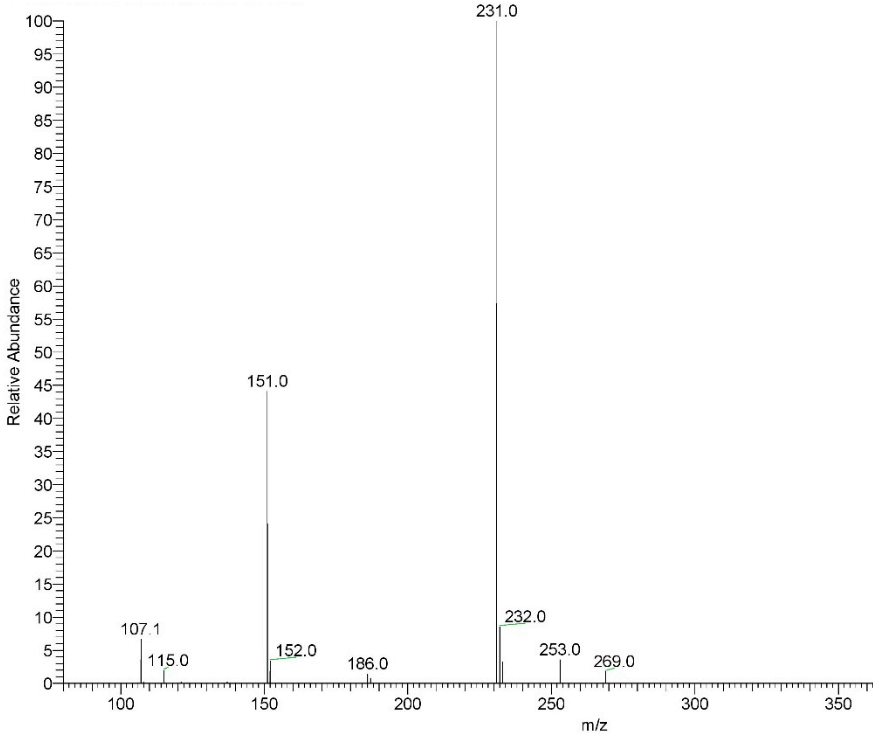

Figure S17. MS (ESI$^-$) spectrum of **K_2 4-HPA-S.**

([M – 2K + H]$^-$, *m/z* 231.0).

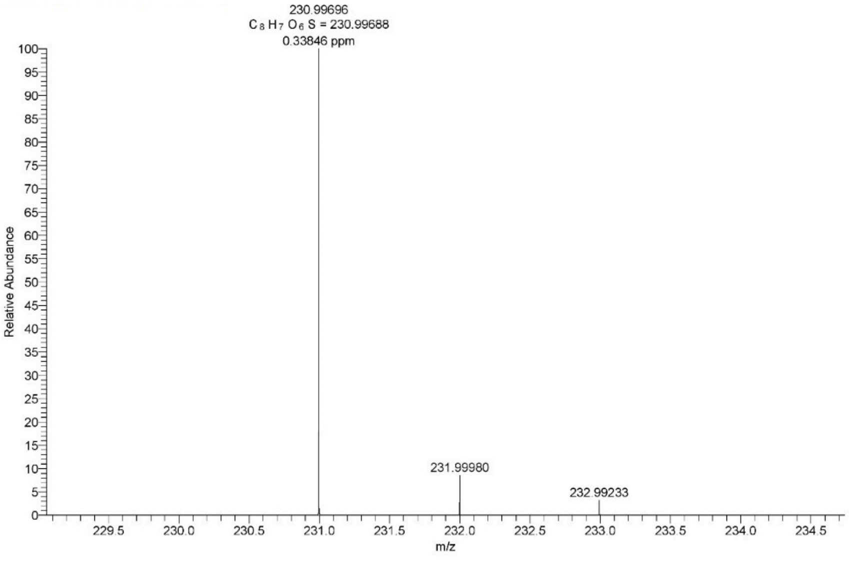

Figure S18. HRMS (ESI$^-$) spectrum of **K_2 4-HPA-S.**

Calculated (for $C_8H_7O_6S^-$) 230.99688, measured 230.99696 (0.3 ppm).

Sodium 2-(4-(sulfonatooxy)phenyl)acetate (Na_2 4-HPA-S)

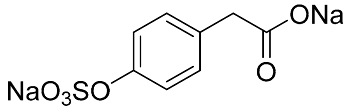

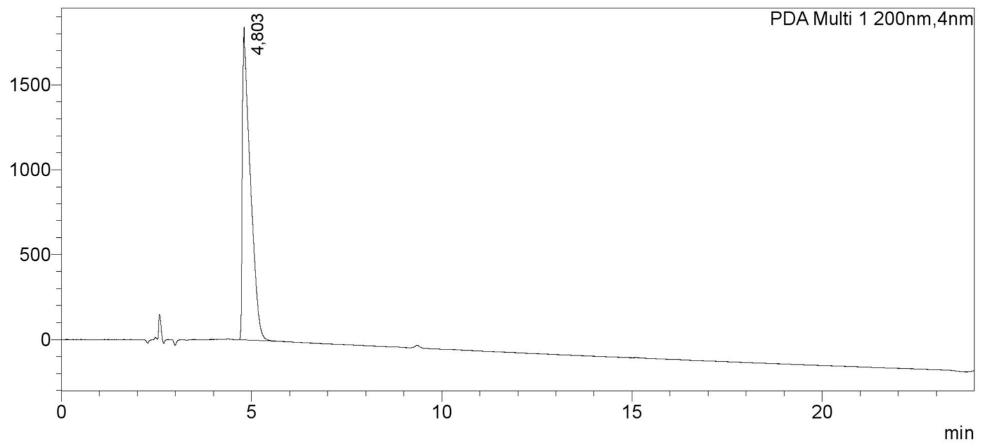

Figure S19. HPLC chromatogram of **Na_2 4-HPA-S**

(RT= 4.803 min, 99% purity)

Table S4. ^{1}H and ^{13}C NMR data for **Na_2 4-HPA-S**

(399.83 MHz for ^{1}H, 100.54 MHz for ^{13}C, DMSO-d_6)

Atom	δ_C	m.	δ_H	n_H	m.	δ_C^{4HPA}	$\delta_C - \delta_C^{4HPA}$
**1**	174.79	S	-	0	-	172.99	1.80
**2**	45.33	T	3.155	2	s	39.79	5.54
**1'**	134.60	S	-	0	-	125.03	9.57
**2'**	129.14	D	7.093	2	m	130.13	-0.99
**3'**	119.82	D	6.979	2	m	114.92	4.90
**4'**	150.96	S	-	0	-	155.95	-4.99

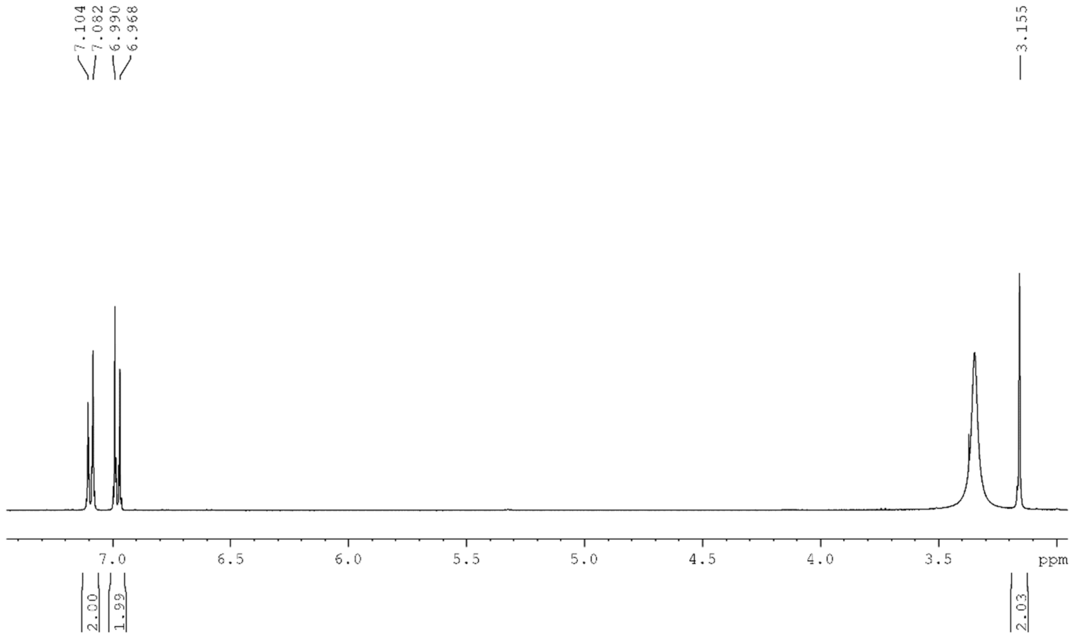

Figure S20. ^{1}H NMR spectrum of **Na_2 4-HPA-S**

(399.83 MHz, DMSO-d_6)

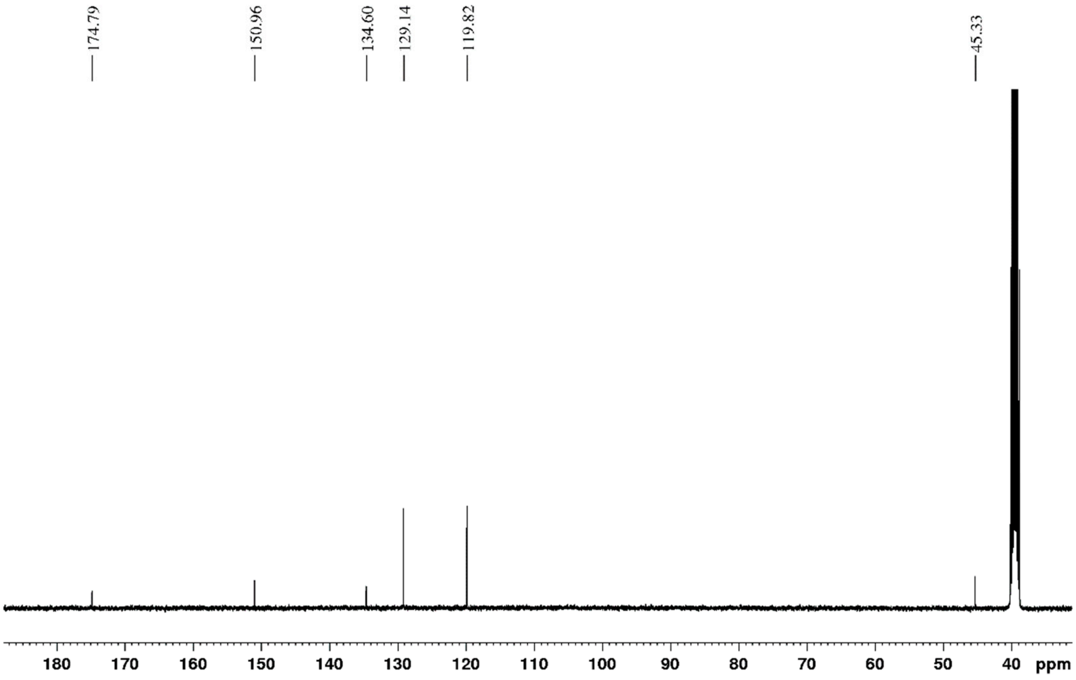

Figure S21. ^{13}C NMR spectrum of **Na$_2$ 4-HPA-S**

(100.54 MHz, DMSO-d_6)

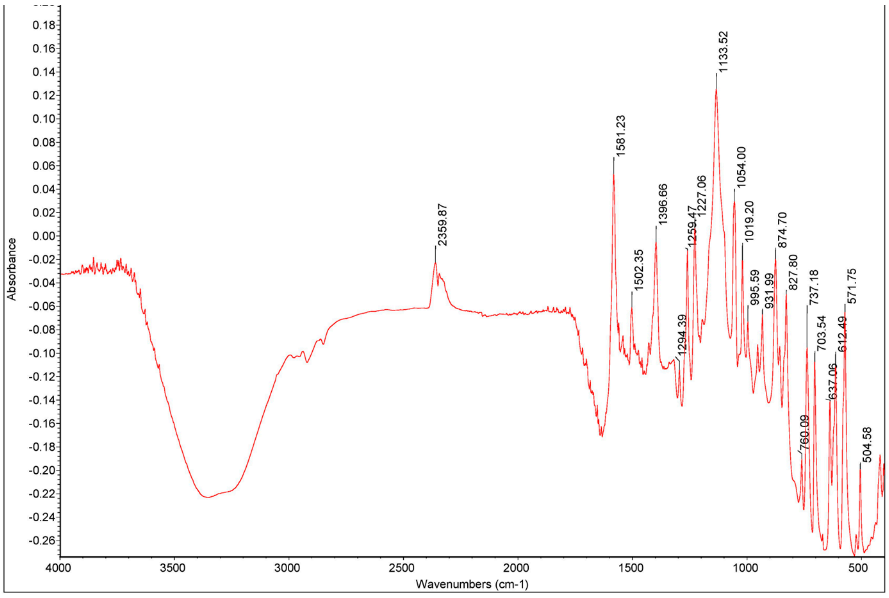

Figure S22. IR spectrum of **Na$_2$ 4-HPA-S.**

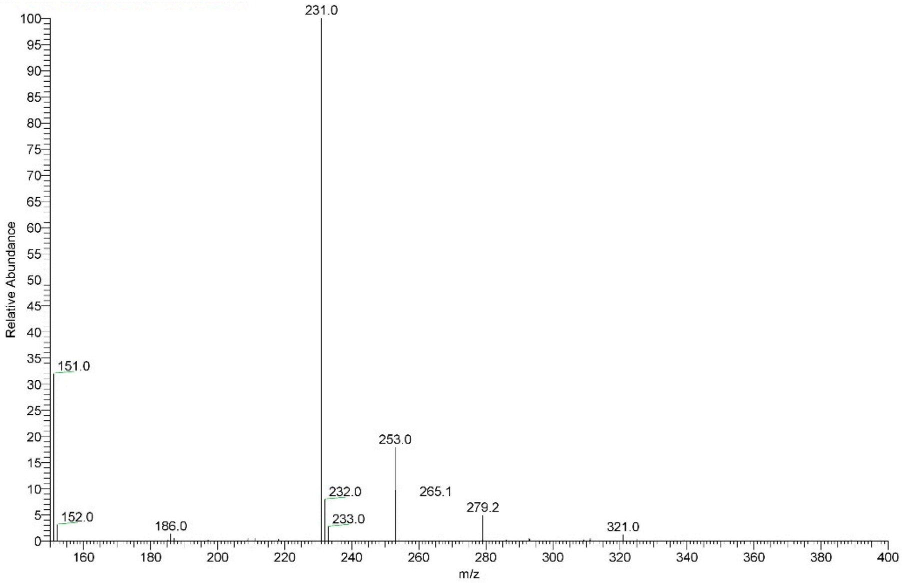

Figure S23. MS (ESI$^-$) spectrum of **Na_2 4-HPA-S.**

([M – 2Na + H]$^-$, *m/z* 231.0).

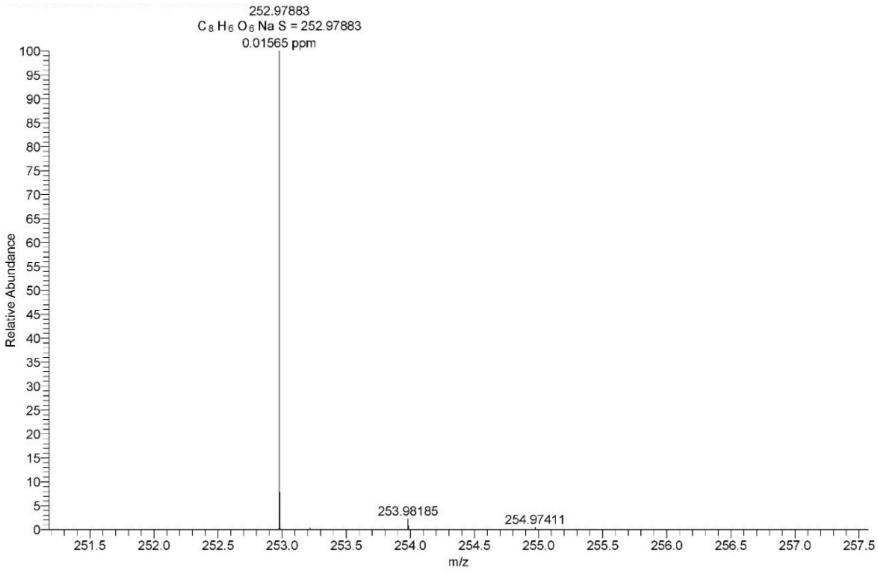

Figure S24. HRMS (ESI$^-$) spectrum of **Na_2 4-HPA-S.**
Calculated (for $C_8H_6O_6NaS^-$) 252.97883, measured 252.97883 (0.0 ppm).

Potassium 3-(4-(sulfonatooxy)phenyl)propanoate (K_2 4-HPP-S)

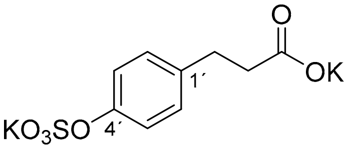

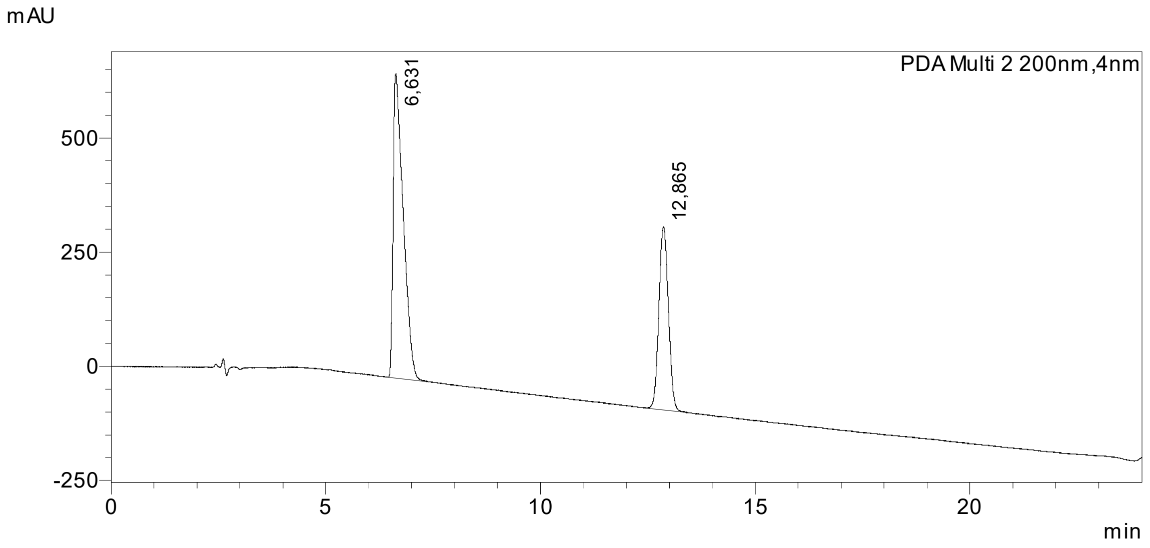

Figure S25. HPLC chromatogram of **K_2 4-HPP-S**

(RT= 6.631 min, 64% purity, the peak at 12.865 min belongs to the starting 4-HPP and might be partially enhanced by degradation during HPLC analysis under acidic conditions)

Table S5. ^{1}H and ^{13}C NMR data for **K_2 4-HPP-S**

(700.13 MHz for ^{1}H, 176.05 MHz for ^{13}C, DMSO-d_6)

**Atom**	δ_C	**m.**	δ_H	n_H	**m.**	***J* [Hz]**	δ_C^{HPP}	$\delta_C - \delta_C^{HPP}$
**1**	174.85	S	-	0	-	-	173.76	1.09
**2**	37.28	T	2.369	2	m	-	35.64	1.64
**3**	30.50	T	2.736	2	m	-	29.50	1
**1'**	136.35	S	-	0	-	-	130.84	5.51
**2'6'**	128.28	D	7.089	2	m	Σ*J* = 8.6	128.97	-0.69
**3'5'**	120.34	D	7.042	2	m	Σ*J* = 8.6	114.97	5.37
**4'**	151.44	S	-	0	-	-	155.43	-3.99

Minority **K 4-HPP**

**Atom**	δ_C	**m.**	δ_H	n_H	m.	***J* [Hz]**	δ_C^{HPP}	$\delta_C - \delta_C^{HPP}$
**1**	174.9	S	-	0	-	-	173.76	1.14
**2**	37.42	T	2.331	2	m	-	35.64	1.78
**3**	30.28	T	2.667	2	m	-	29.50	0.78
**1'**	131.64	S	-	0	-	-	130.84	0.8
**2'6'**	128.86	D	6.973	2	m	Σ*J* = 8.5	128.97	-0.11
**3'5'**	114.92	D	6.643	2	m	Σ*J* = 8.5	114.97	-0.05
**4'**	155.36	S	-	0	-	-	155.43	-0.07

approximate molar ratio **K_2 4-HPP-S** : **K 4-HPP** = 87 : 13

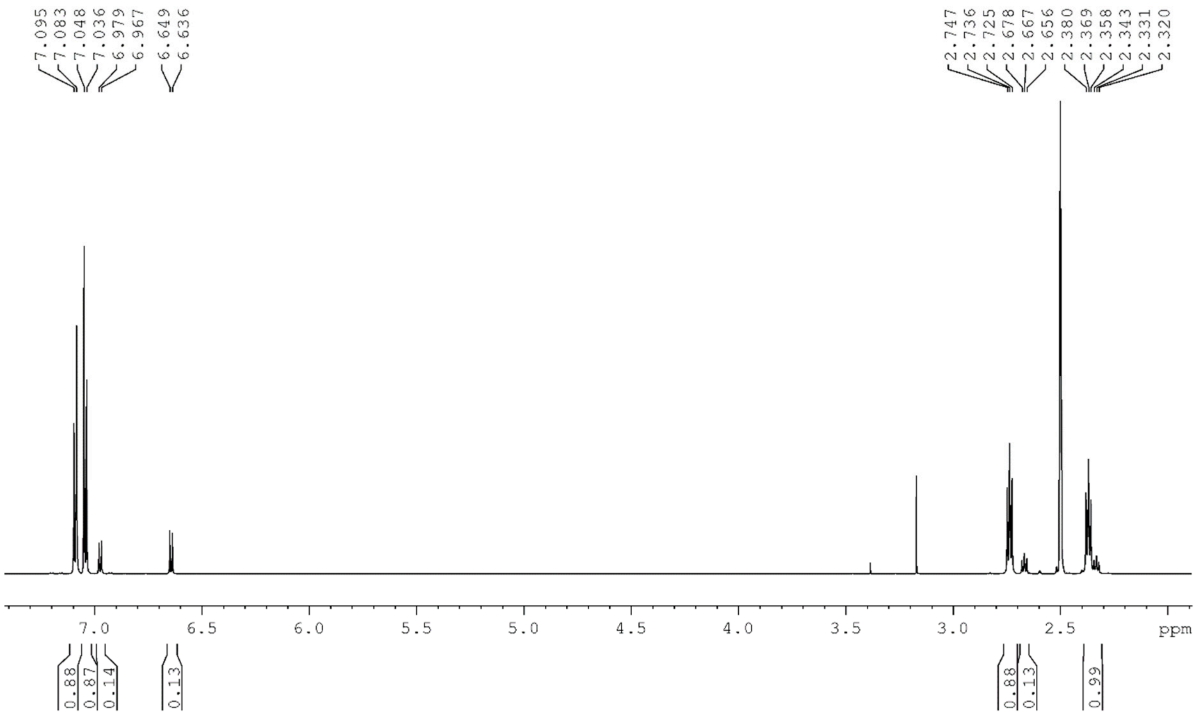

Figure S26. ^{1}H NMR spectrum of **K_2 4-HPP-S**

(700.13 MHz, DMSO-d_6)

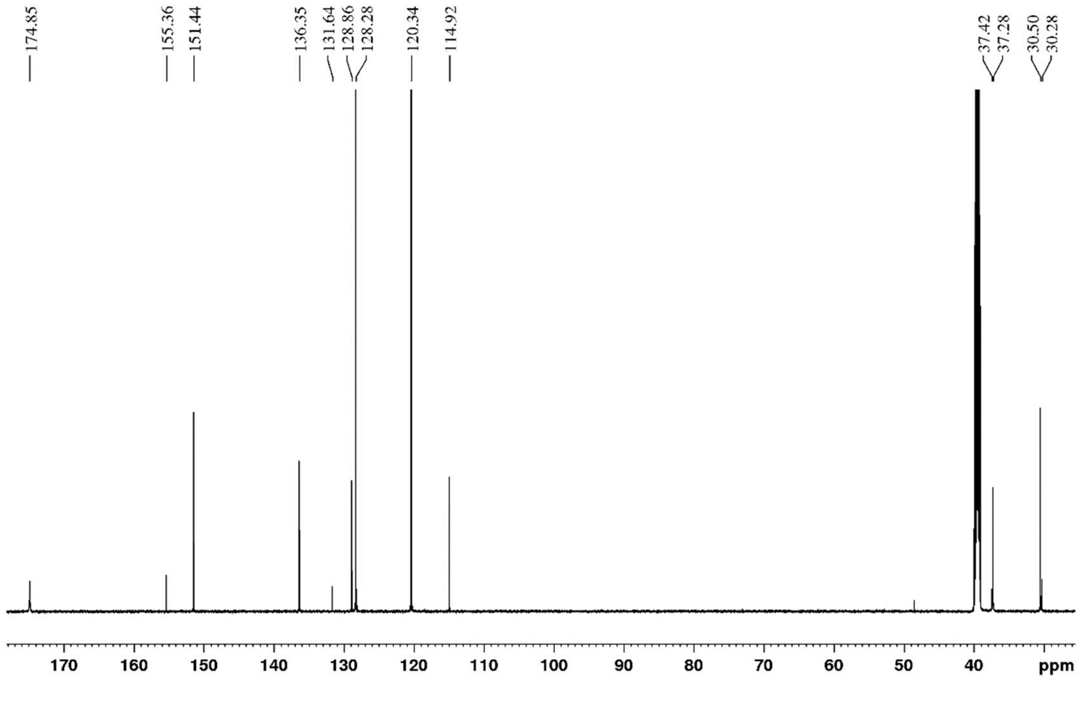

Figure S27. ^{13}C NMR spectrum of **K_2 4-HPP-S**

(176.05 MHz, DMSO-d_6)

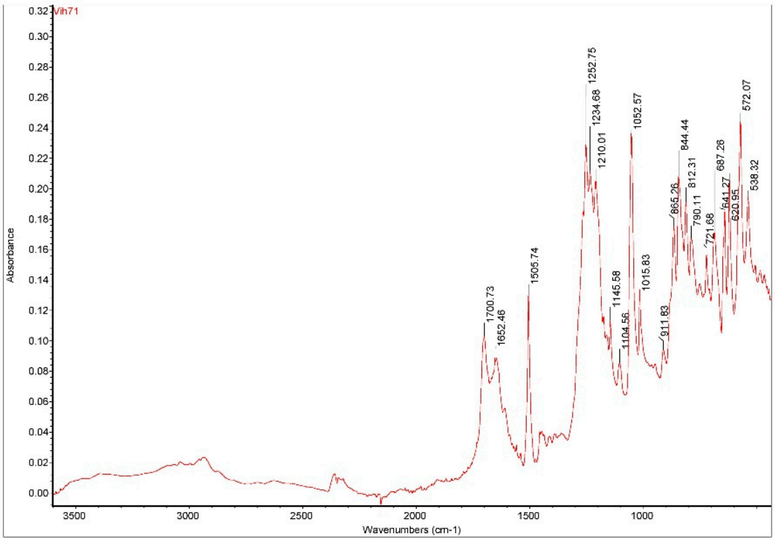

Figure S28. IR spectrum of **K_2 4-HPP-S**.

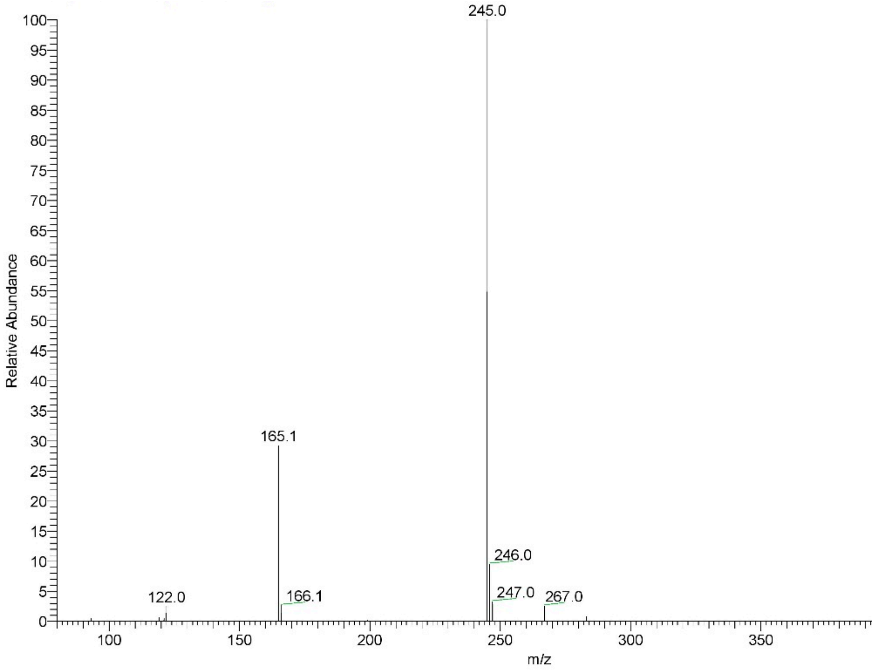

Figure S29. MS (ESI$^-$) spectrum of **K_2 4-HPP-S**.

([M − 2K + H]$^-$, *m/z* 245.0).

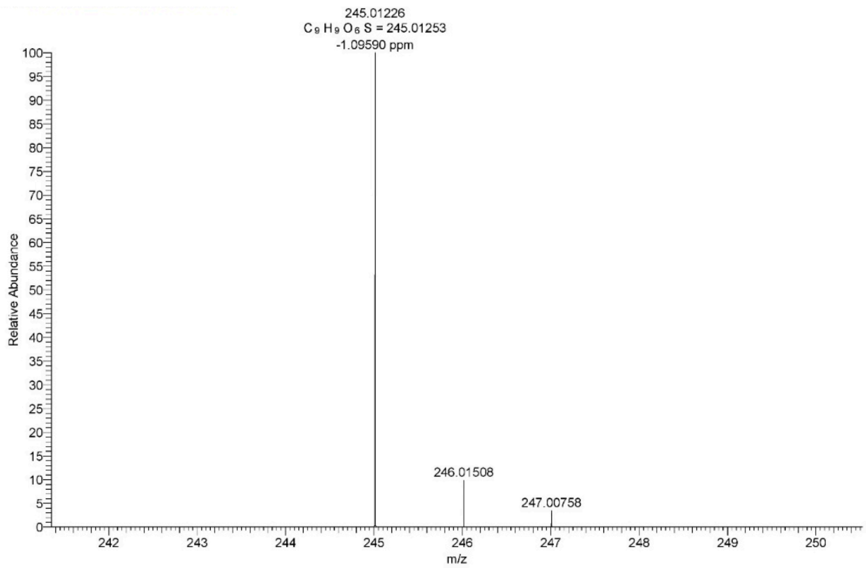

Figure S30. HRMS (ESI$^-$) spectrum of **K$_2$ 4-HPP-S**.

Calculated (for $C_9H_9O_6S^-$) 245.01253, measured 245.01226 (-1.1 ppm).

Sodium 3-(4-(sulfonatooxy)phenyl)propanoate (Na$_2$ 4-HPP-S)

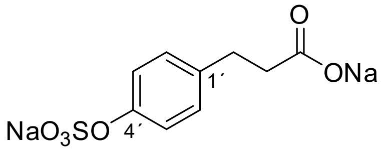

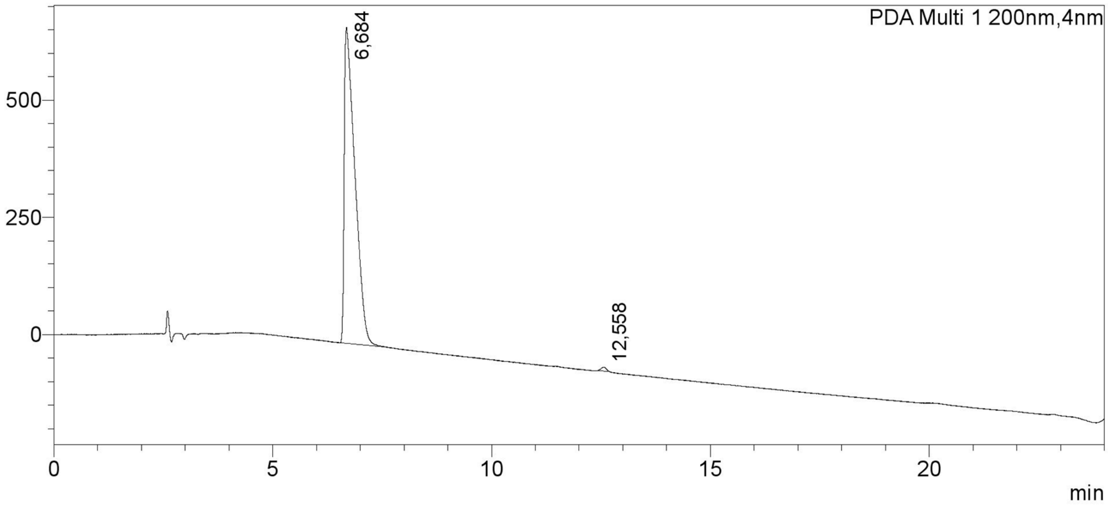

Figure S31. HPLC chromatogram of **Na$_2$ 4-HPP-S**

(RT= 6.684 min, 99% purity)

Table S6. ^{1}H and ^{13}C NMR data for **Na₂ 4-HPP-S**

(600.23 MHz for ^{1}H, 150.93 MHz for ^{13}C, DMSO-d_6)

Atom	δ_C	m.	δ_H	n_H	m.	*J* [Hz]	δ_C^{HPP}	$\delta_C - \delta_C^{HPP}$
**1**	176.36	S	-	0	-	-	173.76	2.60
**2**	40.06	T	2.153	2	m	-	35.64	4.42
**3**	31.90	T	2.704	2	m	-	29.50	2.40
**1'**	137.93	S	-	0	-	-	130.84	7.09
**2'6'**	128.16	D	7.067	2	m	Σ*J* = 8.7	128.97	-0.81
**3'5'**	120.24	D	7.007	2	m	Σ*J* = 8.7	114.97	5.27
**4'**	151.09	S	-	0	-	-	155.43	-4.34

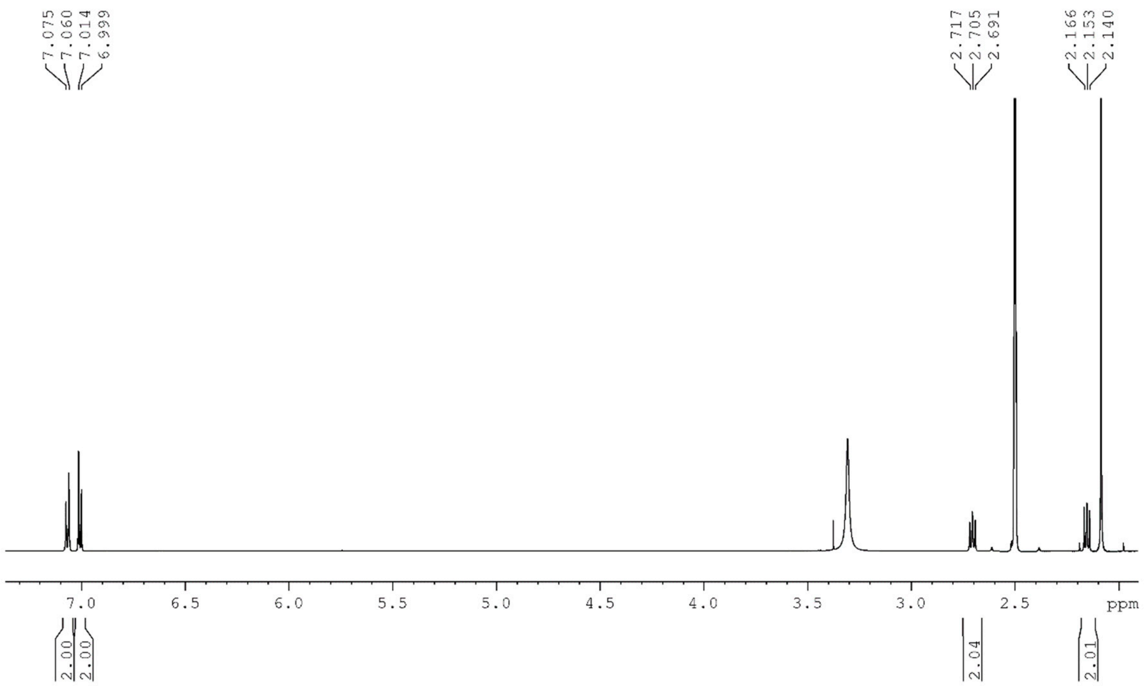

Figure S32. ^{1}H NMR spectrum of **Na₂ 4-HPP-S**

(600.23, DMSO-d_6)

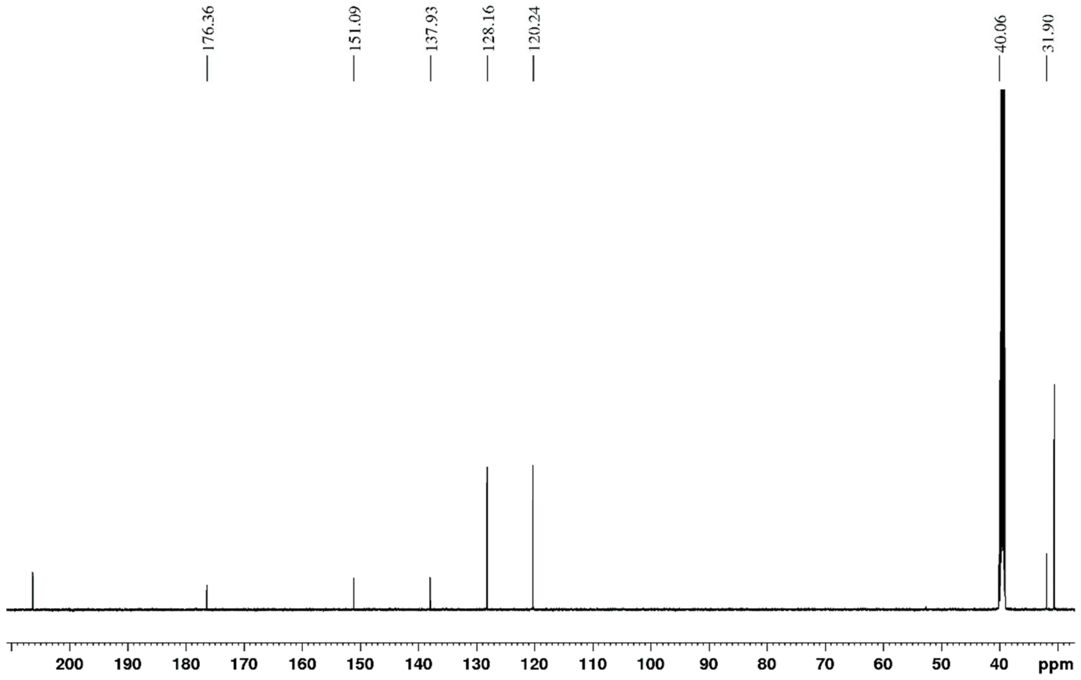

Figure S33. ^{13}C NMR spectrum of **Na_2 4-HPP-S**
(150.93 MHz, DMSO-d_6)

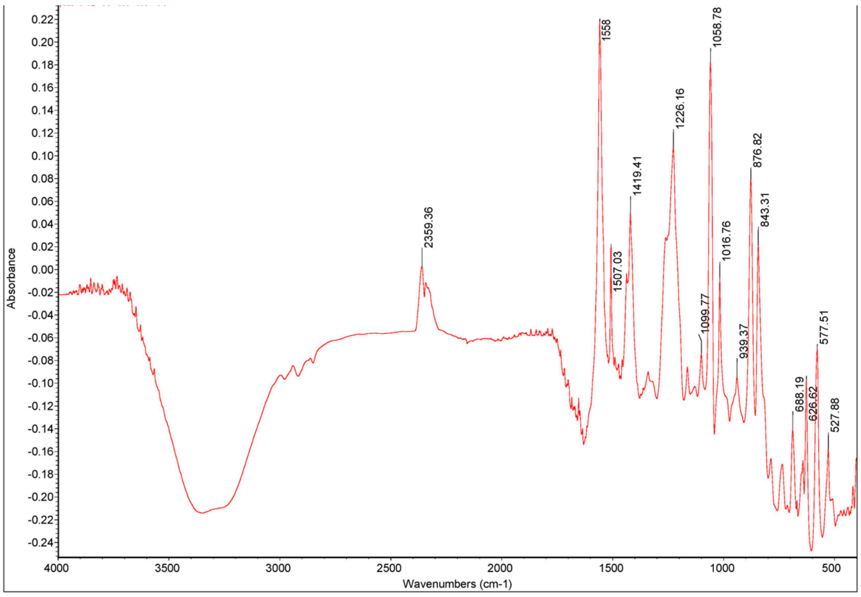

Figure S34. IR spectrum of **Na_2 4-HPP-S**.

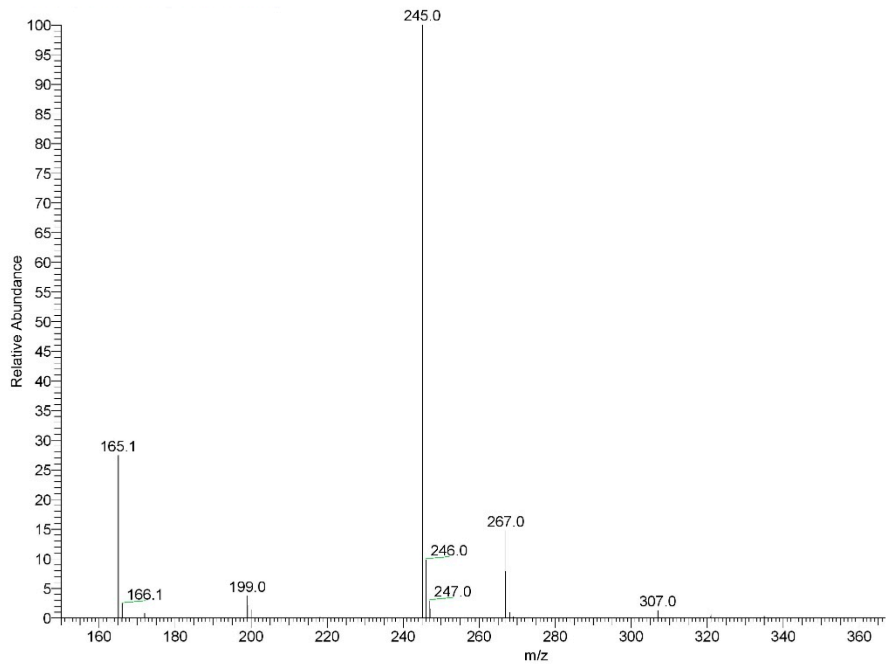

Figure S35. MS (ESI$^-$) spectrum of **Na$_2$ 4-HPP-S**.

([M – 2Na + H]$^-$, *m/z* 245.0; [M – Na]$^-$, *m/z* 267.0).

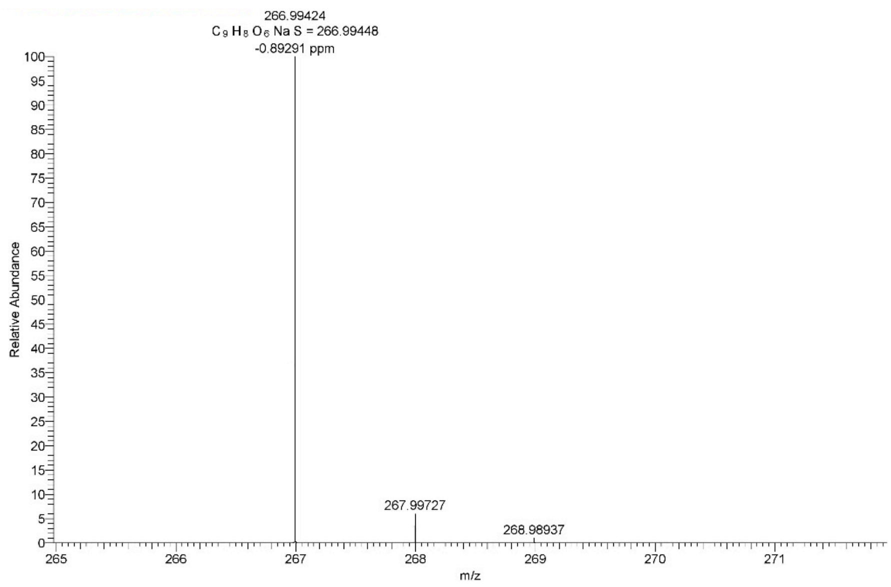

Figure S36. HRMS (ESI$^-$) spectrum of **Na$_2$ 4-HPP-S**.

Calculated (for $C_9H_8O_6NaS^-$) 266.99448, measured 266.99424 (-0.9 ppm).

Potassium 2-(2-hydroxyphenyl)acetate (K 2-HPA) and potassium 2-(2-hydroxy-5-sulfonatophenyl)acetate (K$_2$ 2-HPA-CS)

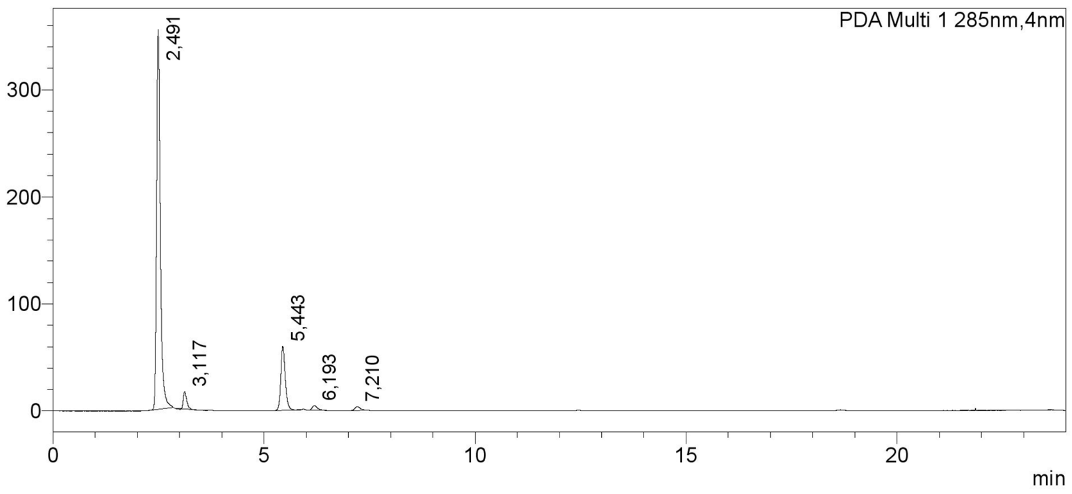

Figure S37. HPLC chromatogram of **K 2-HPA** and **K_2 2-HPA-CS**

(RT= 2.491 min, 79%; RT= 5.443 min, 16%)

Potassium 2-(2-(sulfonatooxy)phenyl)acetate (K 2-HPA)

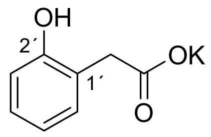

Table S7. ^{1}H and ^{13}C NMR data for **K 2-HPA**.

(399.83 MHz for ^{1}H, 100.54 MHz for ^{13}C, DMSO-d_6)

Atom	δ_C	m.	δ_H	n_H	m.	J [Hz]	δ_C^{2HPA}	$\delta_C - \delta_C^{2HPA}$
**1**	174.24	S	-	0	-	-	172.63	1.61
**2**	40.88	T	3.352	2	s	-	35.23	5.65
**1'**	123.774	S	-	0	-	-	121.75	2.02
**2'**	157.14	S	-	0		-	155.26	1.88
**3'**	115.94	D	6.688	1	dd	8.0, 1.3	114.68	1.26
**4'**	127.15	D	6.987	1	ddd	n.e.	127.67	-0.52
**5'**	117.89	D	6.633	1	ddd	1.3, ΣJ = 14.7	118.58	-0.69
**6'**	130.25	D	6.960	1	dd	n.e.	130.92	-0.67

n.e. … not extracted

Potassium 2-(2-hydroxy-5-sulfonatophenyl)acetate (K_2 2-HPA-CS)

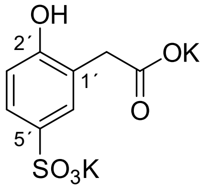

Table S8. ^{1}H and ^{13}C NMR data for **K_2 2-HPA-CS**.

(399.83 MHz for ^{1}H, 100.54 MHz for ^{13}C, DMSO-d_6)

**Atom**	δ_C	**m.**	δ_H	n_H	m.
**1**	174.13	S	-	0	-
**2**	40.95	T	3.347	2	s
**1’**	122.42	S	-	0	-
**2’**	157.51	S	-	0	-
**3’**	114.65	D	6.593	1	m
**4’**	124.89	D	7.267	1	m
**5’**	138.25	S	-	0	-
**6’**	128.00	D	7.26^{H}	1	m

^{H} … HSQC readout

approximate molar ratio **K 2-HPA** : **K_2 2-HPA-CS** = 1 : 1

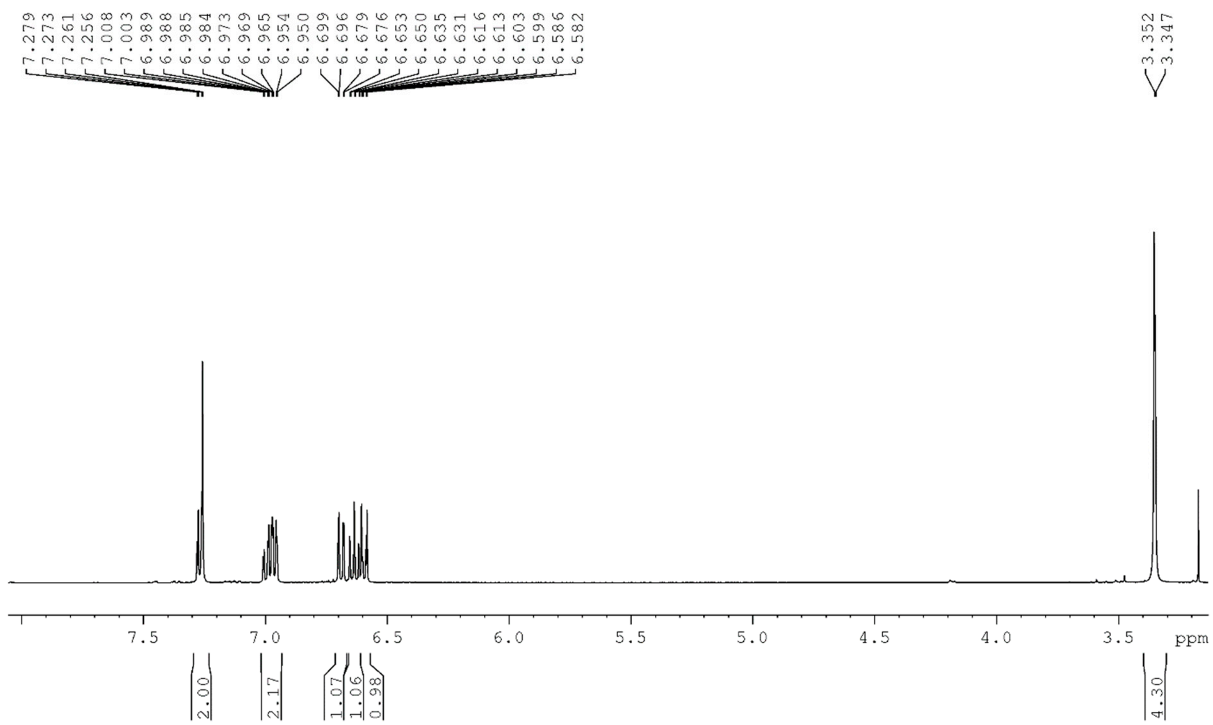

Figure S38. ^{1}H NMR spectrum of **K 2-HPA** and **K_2 2-HPA-CS**.

(399.83 MHz, DMSO-d_6)

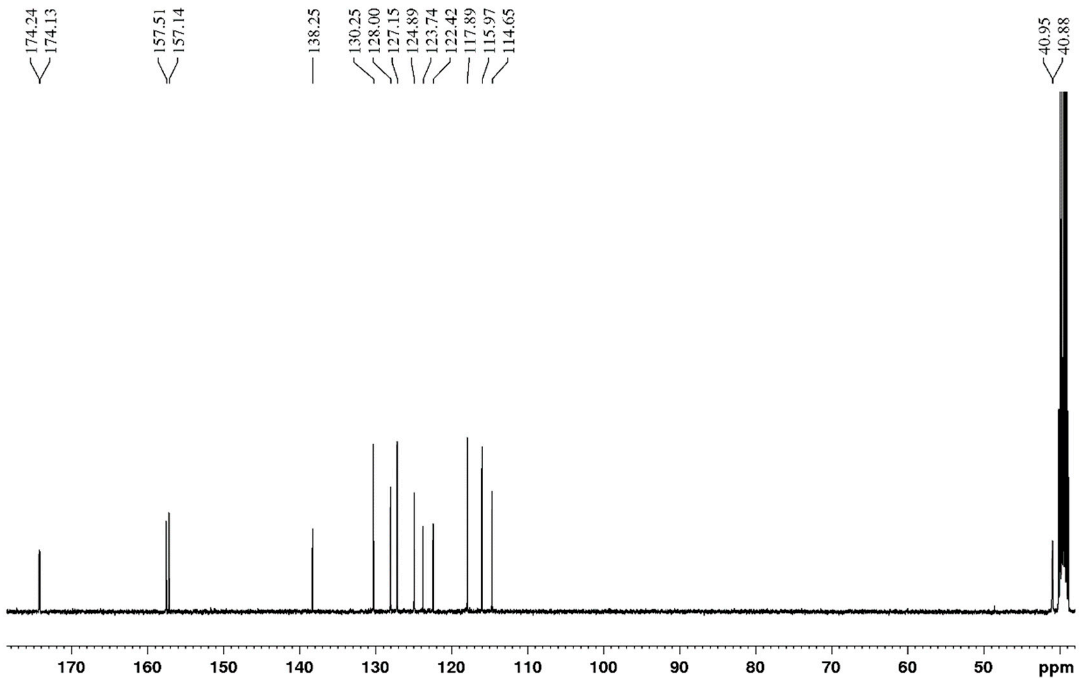

Figure S39. ^{13}C NMR spectrum of **K 2-HPA** and **K_2 2-HPA-CS**.
(100.54 MHz, DMSO-d_6)

3-(4,5-Dihydroxy-2-sulfophenyl)propanoic acid (DHPP-CS)

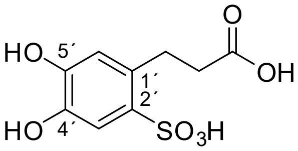

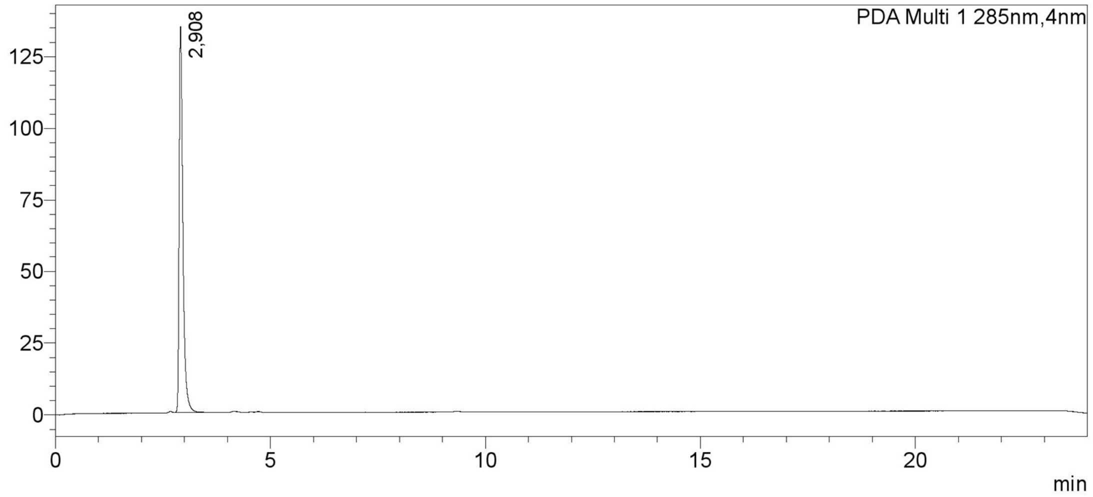

Figure S40. HPLC chromatogram of **DHPP-CS**
(RT= 2.908 min, 99% purity)

Table S9. ^{1}H and ^{13}C NMR data for **DHPP-CS**.

(399.83 MHz for ^{1}H, 100.54 MHz for ^{13}C, DMSO-d_6)

Atom	δ_C	m.	δ_H	n_H	m.	J [Hz]
**1**	174.39	S	-	0	-	-
**2**	35.53	T	2.489	2	m	Σ*J* = 15.9
**3**	27.30	T	3.015	2	m	Σ*J* = 15.9
**1'**	129.33	S	-	0	-	-
**2'**	116.21	D	6.517	1	s	-
**3'**	157.04	S	-	0	-	-
**4'**	112.64	S	-	0	-	-
**5'**	128.55	D	7.210	1	s	-
**6'**	119.76	S	-	0	-	-

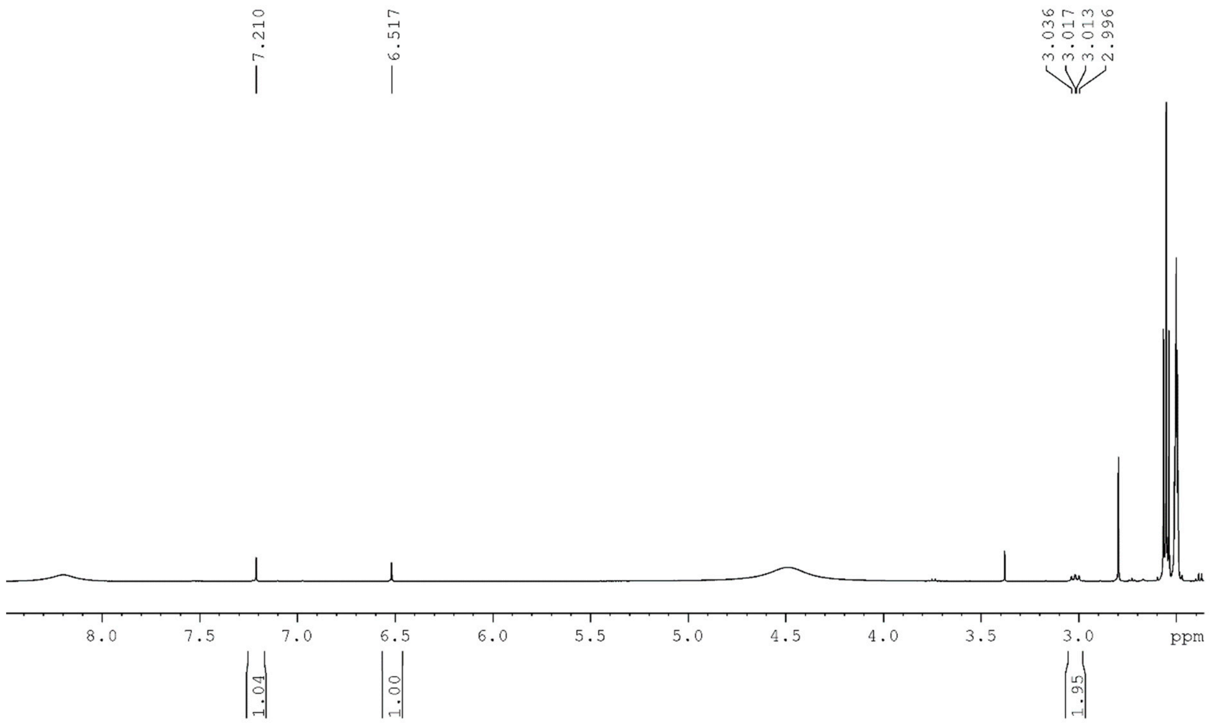

Figure S41. ^{1}H NMR spectrum of **DHPP-CS**.

(399.83 MHz, DMSO-d_6)

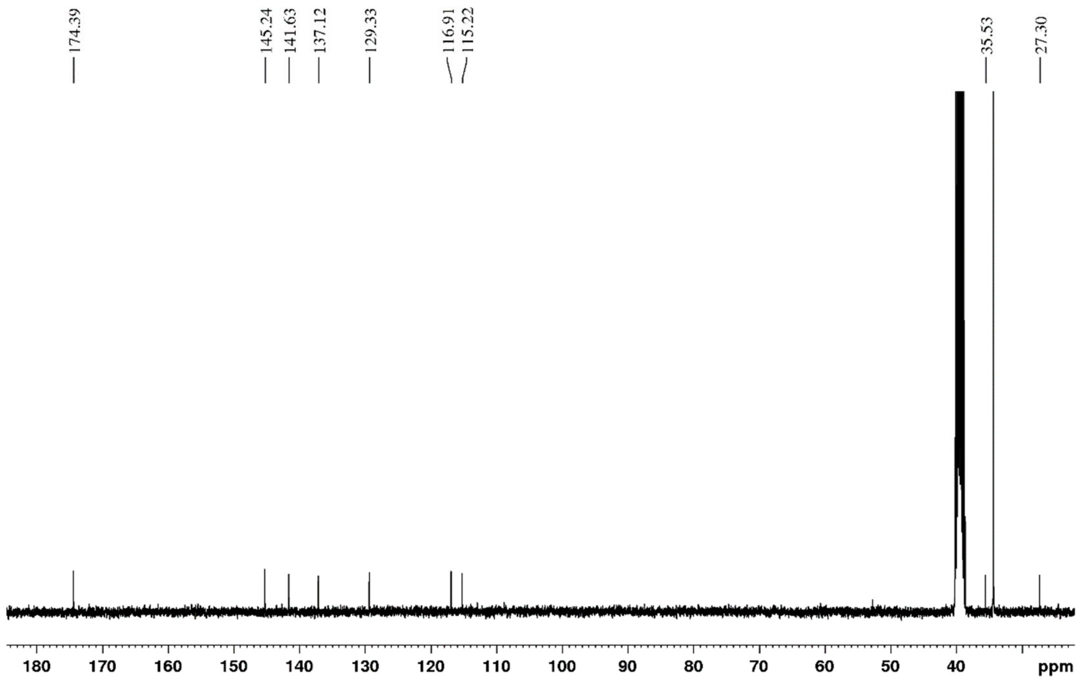

Figure S42. ^{13}C NMR spectrum of **DHPP-CS**

(100.54 MHz, DMSO-d_6)

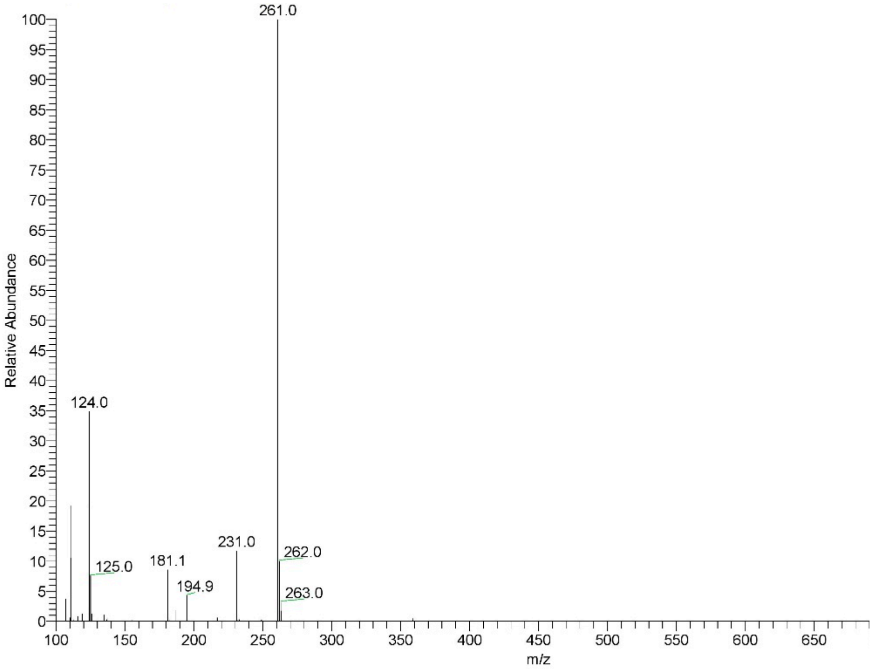

Figure S43. MS (ESI$^-$) spectrum of **DHPP-CS**.

([M – H]$^-$, *m/z* 261.0).

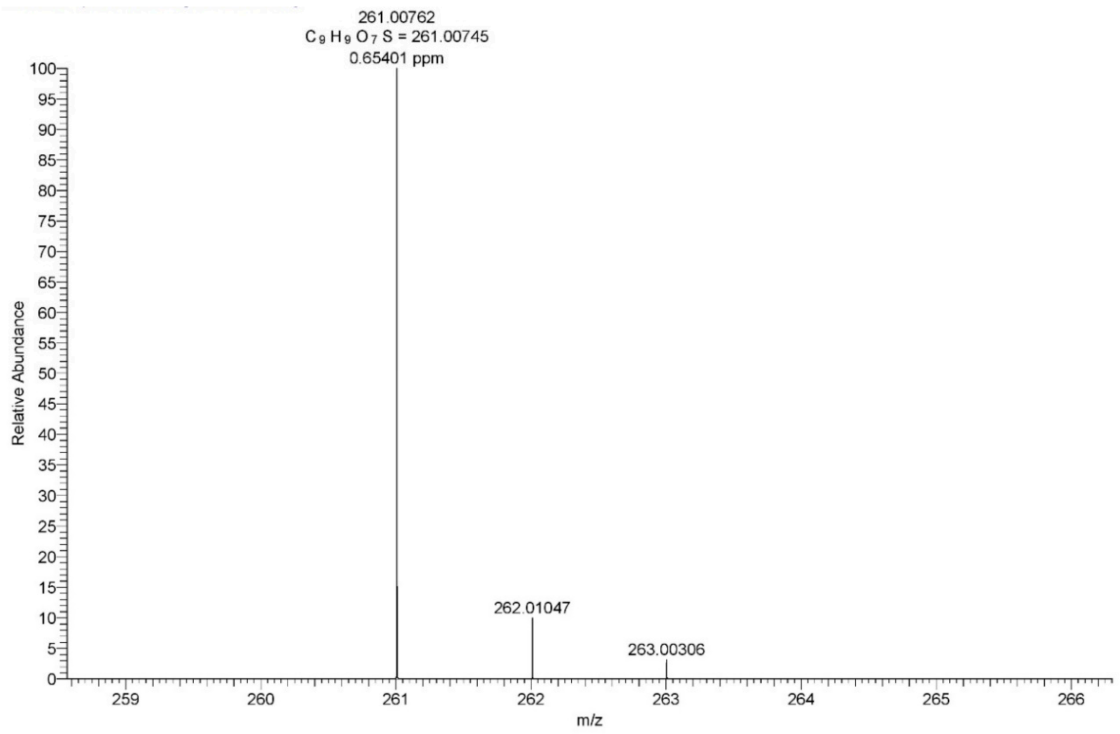

Figure S44. HRMS (ESI$^-$) spectrum of **DHPP-CS**.

Calculated (for $C_9H_9O_7S^-$) 261.00745, measured 261.00762 (0.7 ppm).

3,4-dihydroxyphenylacetic acid 3-*O*-sulfate (DHPA-3'-S) and 3,4-dihydroxyphenylacetic acid 4-*O*-sulfate (DHPA-4'-S)

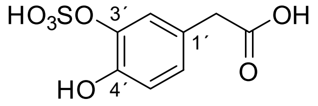

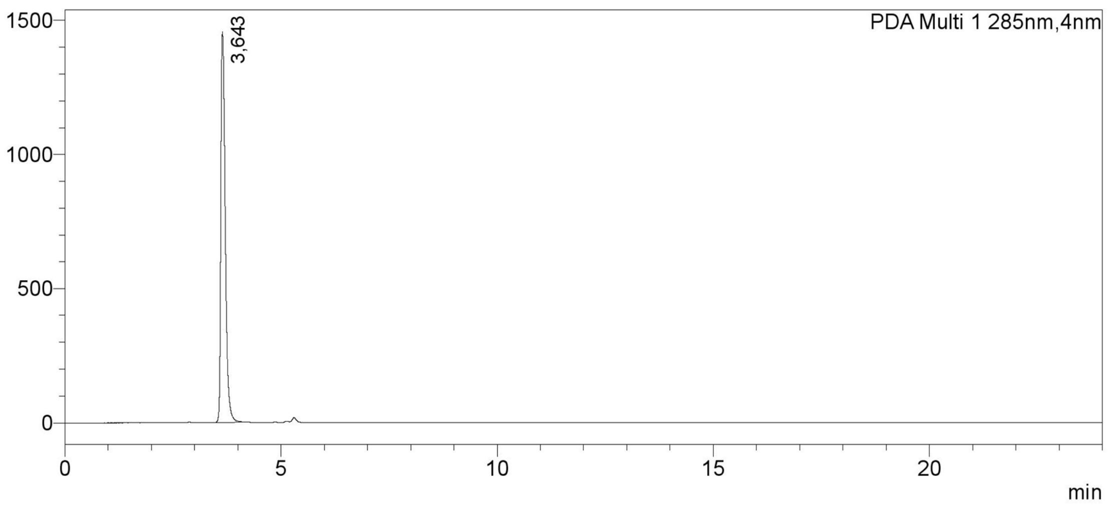

Figure S45. HPLC chromatogram of **DHPA-3'-S** and **DHPA-4'-S**

(RT= 3.643 min, 99%)

Table S10. ^{1}H and ^{13}C NMR data for **DHPA-3'-S** and **DHPA-4'-S**

(600.23 MHz for ^{1}H, 150.93 MHz for ^{13}C, DMSO-d_6)

DHPA-3'-S

Atom	δ_C	m.	δ_H	n_H	m.	*J* [Hz]	δ_C^{DHPA}	$\delta_C - \delta_C^{DHPA}$
**1**	173.74	S	-	0	-	-	173.00	0.74
**2**	41.13^x	T	3.33^H	2	m	-	40.14	0.99
**1'**	127.17^x	S	-	0	-	-	125.60	1.57
**2'**	123.88	D	6.980	1	d	2.2	116.58	7.30
**3'**	140.32	S	-	0	-	-	144.91	-4.59
**4'**	147.55	S	-	0	-	-	143.94	3.61
**5'**	116.76	D	6.724	1	d	8.2	115.28	1.48
**6'**	127.17	D	6.829	1	dd	8.2, 2.2	119.94	7.23

^{H} … HSQC readout, ^{x} … broad signal

DHPA-4'-S

Atom	δ_C	m.	δ_H	n_H	m.	*J* [Hz]	δ_C^{DHPA}	$\delta_C - \delta_C^{DHPA}$
**1**	173.74	S	-	0	-	-	173.00	0.74
**2**	41.49^x	T	3.33^H	2	m	-	40.14	1.35
**1'**	133.13^x	S	-	0	-	-	125.60	7.53
**2'**	118.14	D	6.710	1	d	2.1	116.58	1.56
**3'**	148.66	S	-	0	-	-	144.91	3.75
**4'**	139.22	S	-	0	-	-	143.94	-4.72
**5'**	122.60	D	6.975	1	d	8.1	115.28	7.32
**6'**	120.19	D	6.598	1	dd	8.1, 2.1	119.94	0.25

^{H} … HSQC readout, ^{x} … broad signal

approximate molar ratio **DHPA-3'-S** : **DHPP-A'-S** = 85 : 15

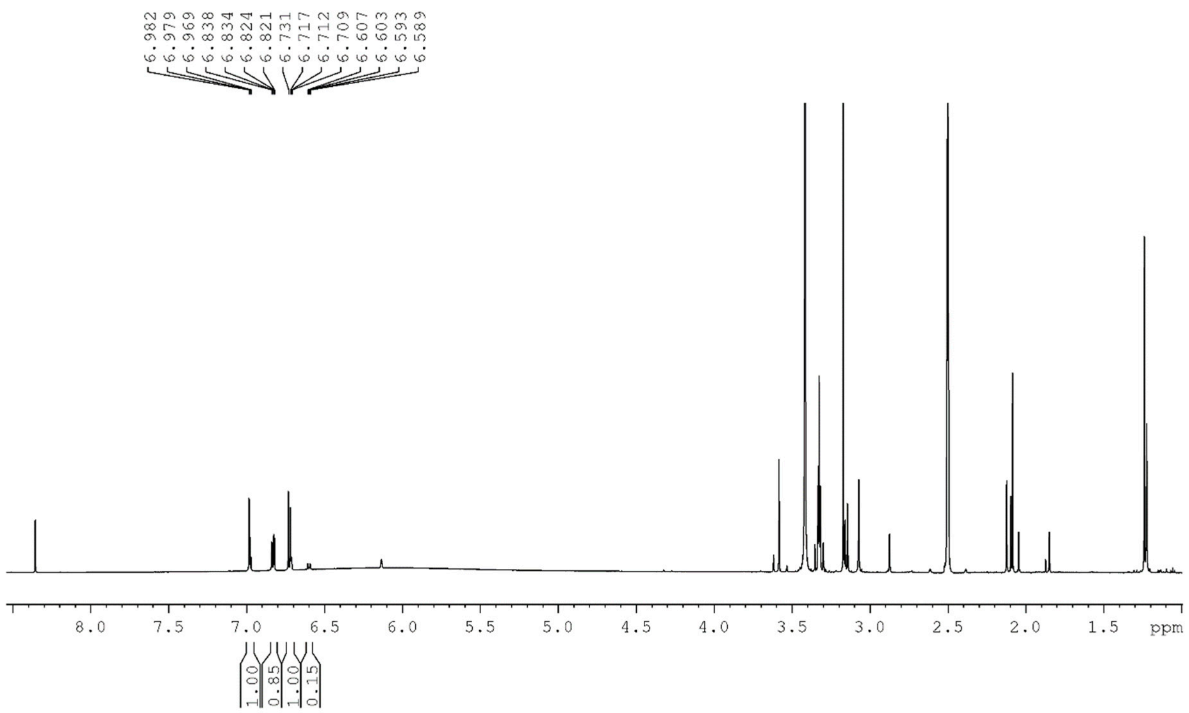

Figure S46. ^{1}H NMR spectrum of **DHPA-3'-S and DHPA-4'-S**
(600.23 MHz, DMSO-d_6)

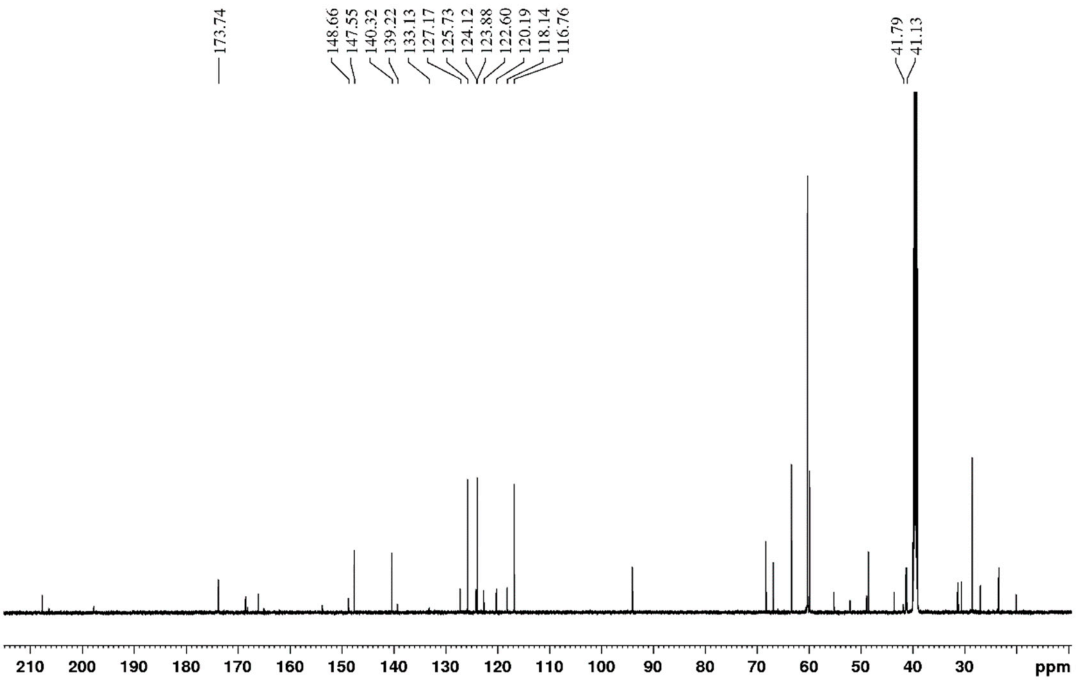

Figure S47. ^{13}C NMR spectrum of **DHPA-3'-S and DHPA-4'-S**.
(150.93 MHz, DMSO-d_6)

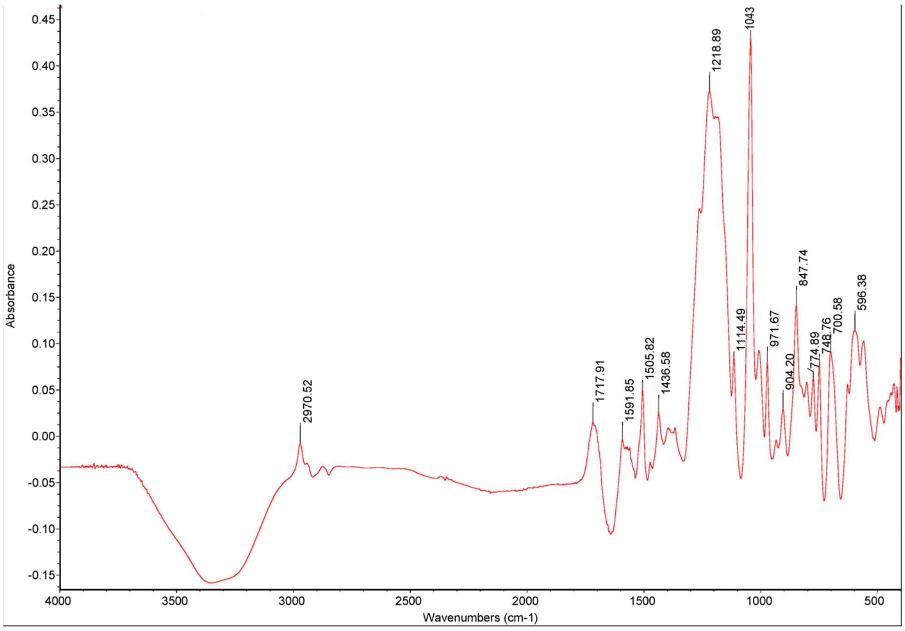

Figure S48. IR spectrum of **DHPA-3’-S** and **DHPA-4’-S**.

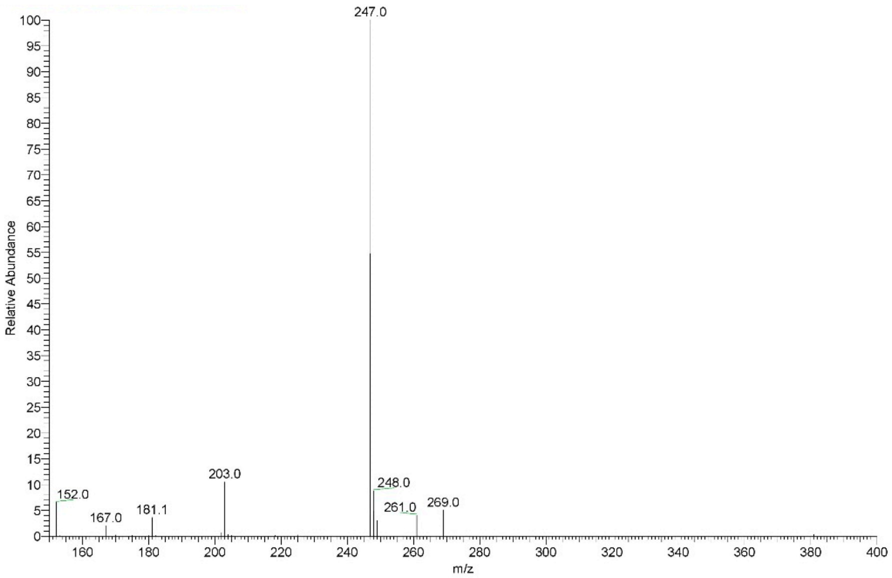

Figure S49. MS (ESI$^-$) spectrum of **DHPA-S**.

([M − H]$^-$, *m/z* 247.0).

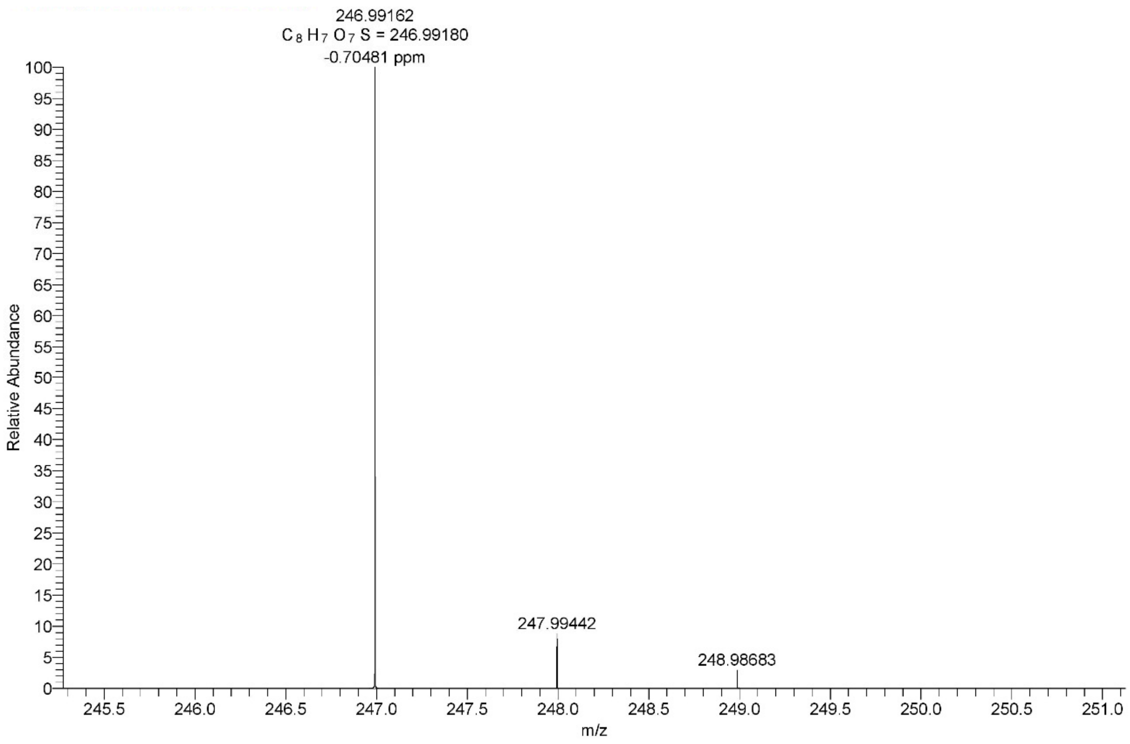

Figure S50. HRMS (ESI⁻) spectrum of compound **DHPA-S**.
Calculated (for $C_8H_7O_7S^-$) 246.99180, measured 246.99162 (-0.7 ppm).

3,4-Dihydroxyphenylpropionic acid sulfates (DHPP-S): 3,4-dihydroxyphenylpropionic acid 3′-*O*-sulfate (DHPP-3′-S) and 3,4-dihydroxyphenylpropionic acid 4′-*O*-sulfate (DHPP-4′-S)

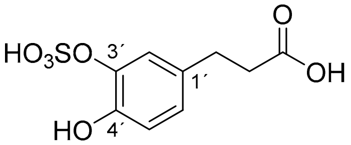

(+ 4´-*O*-sulfate DHPP-4´-S)

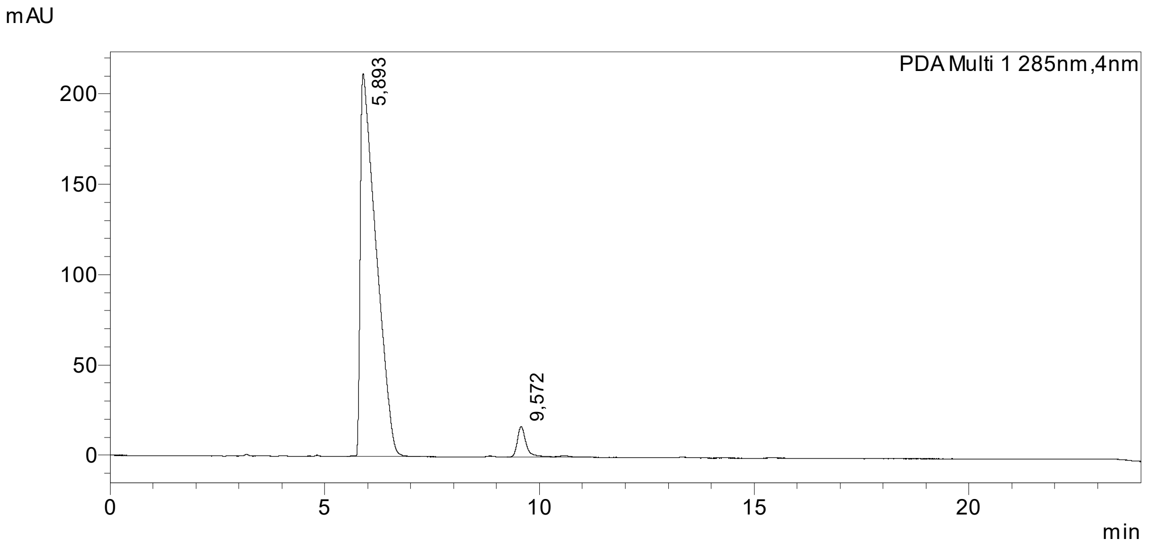

Figure S51. HPLC chromatogram of **DHPP-3'-S** and **DHPP-4'-S**

(RT= 5.839 min, 96%; the peak at 9.572 min belongs to the starting DHPP)

Table S11. ^{1}H and ^{13}C NMR data of **DHPP-3'-S** and **DHPP-4'-S**

(399.83 MHz for ^{1}H, 100.54 MHz for ^{13}C, DMSO-d_6)

DHPP-3'-S

Atom	δ_C	m.	δ_H	n_H	m.	J [Hz]	δ_C^{DHPP}	$\delta_C - \delta_C^{DHPP}$
**1**	174.54	S	-	0	-	-	173.76	0.78
**2**	36.42	T	2.377	2	m	-	35.61	0.81
**3**	29.85	T	2.676	2	m	-	29.68	0.17
**1'**	132.37	S	-	0	-	-	131.60	0.77
**2'**	122.89	D	6.925	1	d	2.1	115.55	7.34
**3'**	140.46	S	-	0	-	-	144.90	-4.44
**4'**	147.23	S	-	0	-	-	143.30	3.93
**5'**	116.95	D	6.706	1	d	8.2	115.36	1.59
**6'**	124.52	D	6.797	1	dd	8.2, 2.2	118.65	5.87

DHPP-4'-S

Atom	δ_C	m.	δ_H	n_H	m.	J [Hz]	δ_C^{DHPP}	$\delta_C - \delta_C^{DHPP}$
**1**	174.54	S	-	0	-	-	173.76	0.78
**2**	36.42	T	2.396	2	m	-	35.61	0.81
**3**	30.26	T	2.676	2	m	-	29.68	0.58
**1'**	138.3^x	S	-	0	-	-	131.60	6.7
**2'**	117.00	D	6.666	1	d	2.1	115.55	1.45
**3'**	148.85	S	-	0	-	-	144.90	3.95
**4'**	138.9^x	S	-	0	-	-	143.30	-4.4
**5'**	122.92	D	6.952	1	d	8.2	115.36	7.56
**6'**	119.06	D	6.579	1	dd	8.2, 2.1	118.65	0.41

approximate molar ratio **DHPP-3'-S** : **DHPP-4'-S** = 87 : 13

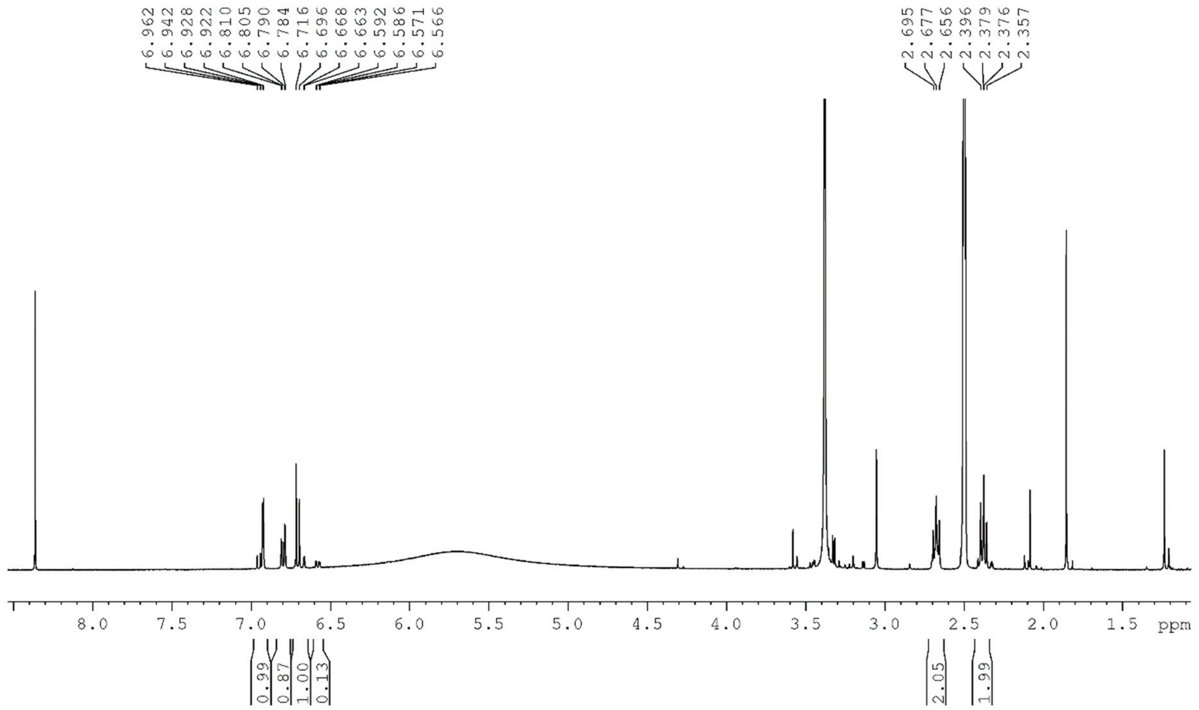

Figure S52. ^{1}H NMR spectrum of **DHPP-3′-S** and **DHPP-4′-S**.

(399.83 MHz, DMSO-d_6)

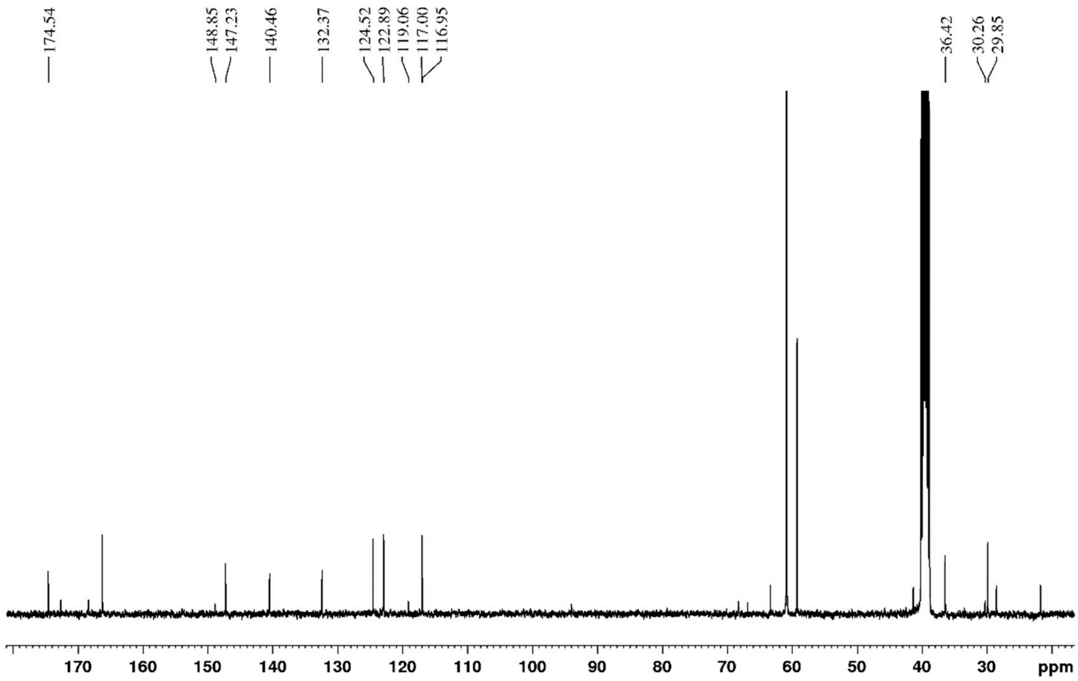

Figure S53. ^{13}C NMR spectrum of **DHPP-3′-S** and **DHPP-4′-S**.
(100.54 MHz, DMSO-d_6)

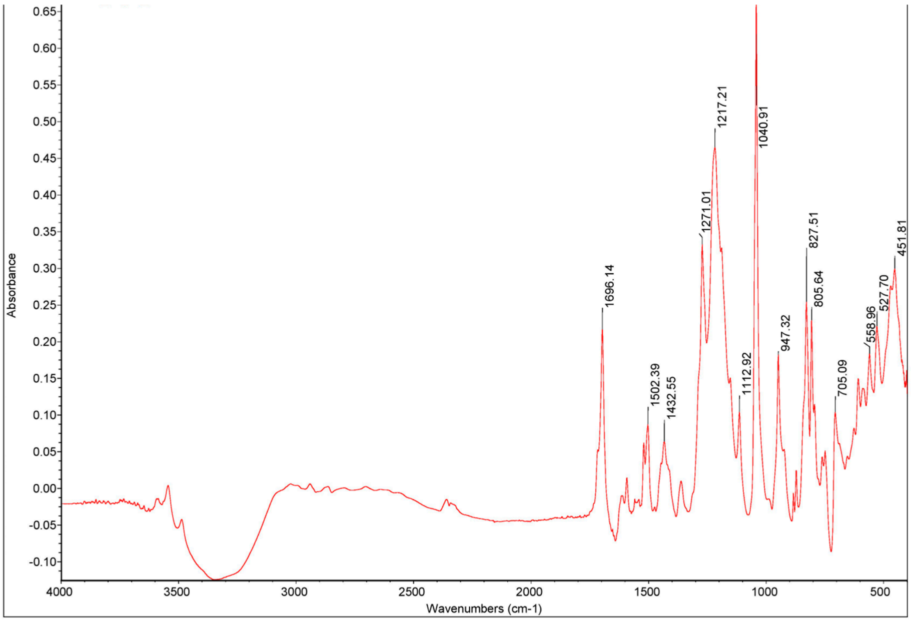

Figure S54. IR spectrum of **DHPP-3′-S** and **DHPP-4’-S**.

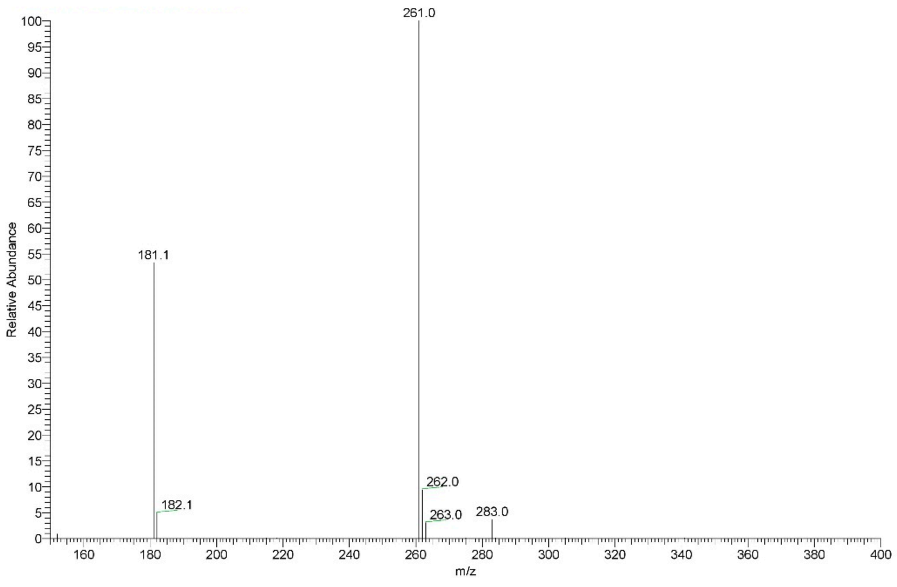

Figure S55. MS (ESI$^-$) spectrum of **DHPP-S**.

([M − H]$^-$, *m/z* 261.0).

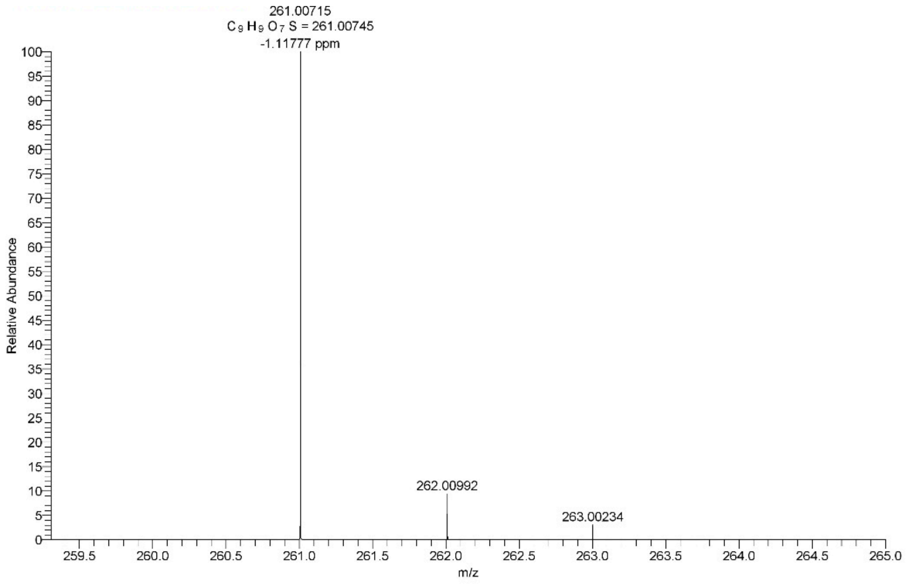

Figure S56. HRMS (ESI$^-$) spectrum of **DHPP-S**.

Calculated (for $C_9H_9O_7S^-$) 261.00745, measured 261.00715 (-1.1 ppm).

Sodium dihydroxyphenylacetate sulfates (Na$_2$ DHPA-S): sodium 2-(4-hydroxy-3-(sulfonatooxy)phenyl)acetate (DHPA-3′-S) and sodium 2-(3-hydroxy-4-(sulfonatooxy)phenyl)acetate (DHPA-4′-S)

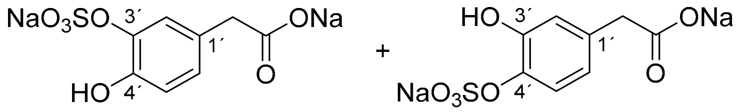

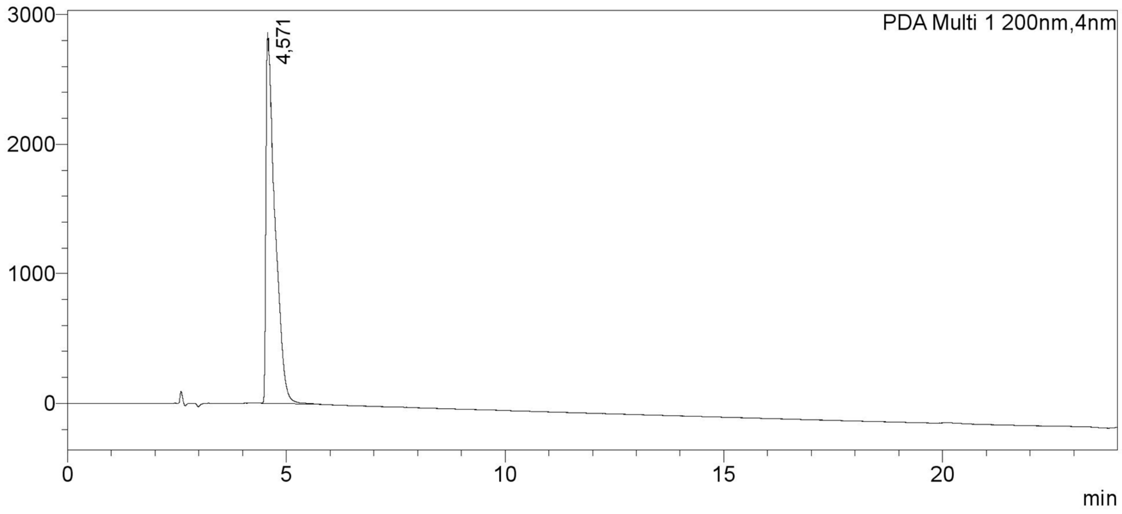

Figure S57. HPLC chromatogram of **Na_2 DHPA-3'-S** and **Na_2 DHPA-4'-S** (RT= 4.571 min, 99%)

Table S12. ^{1}H and ^{13}C NMR data of **Na_2 DHPA-3'-S** and **Na_2 DHPA-4'-S** (600.23 MHz for ^{1}H, 150.93 MHz for ^{13}C, DMSO-d_6)

Na_2 DHPA-3'-S

Atom	δ_C	m.	δ_H	n_H	m.	*J* [Hz]	δ_C^{DHPA}	δ_C - δ_C^{DHPA}
**1**	175.27	S	-	0	-	-	173.00	2.27
**2**	44.90	T	3.101	2	br s	-	40.14	4.76
**1'**	130.76^x	S	-	0	-	-	125.60	5.16
**2'**	123.95	D	6.950	1	d	2.2	116.58	7.37
**3'**	139.97	S	-	0	-	-	144.91	-4.94
**4'**	146.99^x	S	-	0	-	-	143.94	3.05
**5'**	116.36	D	6.649	1	d	8.2	115.28	1.08
**6'**	125.74	D	6.824	1	dd	8.2, 2.2	119.94	5.80

^{x} … broad signal

Na_2 DHPA-4'-S

Atom	δ_C	m.	δ_H	n_H	m.	*J* [Hz]	δ_C^{DHPA}	δ_C - δ_C^{DHPA}
**1**	175.07	S	-	0	-	-	173.00	2.07
**2**	45.52	T	3.110	2	br s	-	40.14	5.38
**1'**	136.77	S	-	0	-	-	125.60	11.17
**2'**	118.19	D	6.737	1	d	2.1	116.58	1.61
**3'**	148.87	S	-	0	-	-	144.91	3.96
**4'**	138.38	S	-	0	-	-	143.94	-5.56
**5'**	122.26	D	6.869	1	d	8.1	115.28	6.98
**6'**	119.72	D	6.563	1	dd	8.1, 2.1	119.94	-0.22

approximate molar ratio **Na_2 DHPA-3'-S**: **Na_2 DHPA-4'-S** = 9 : 91

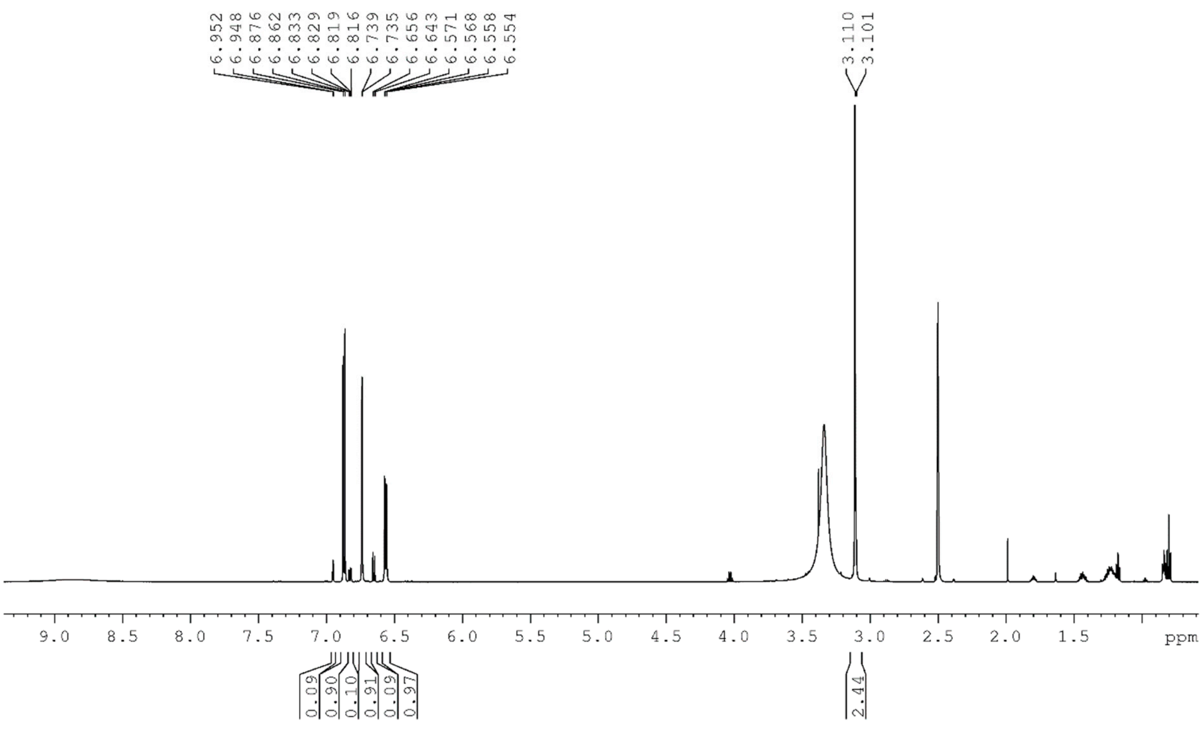

Figure S58. ^{1}H NMR spectrum of **Na_2 DHPA-3'-S** and **Na_2 DHPA-4'-S**.
(600.23 MHz, DMSO-d_6)

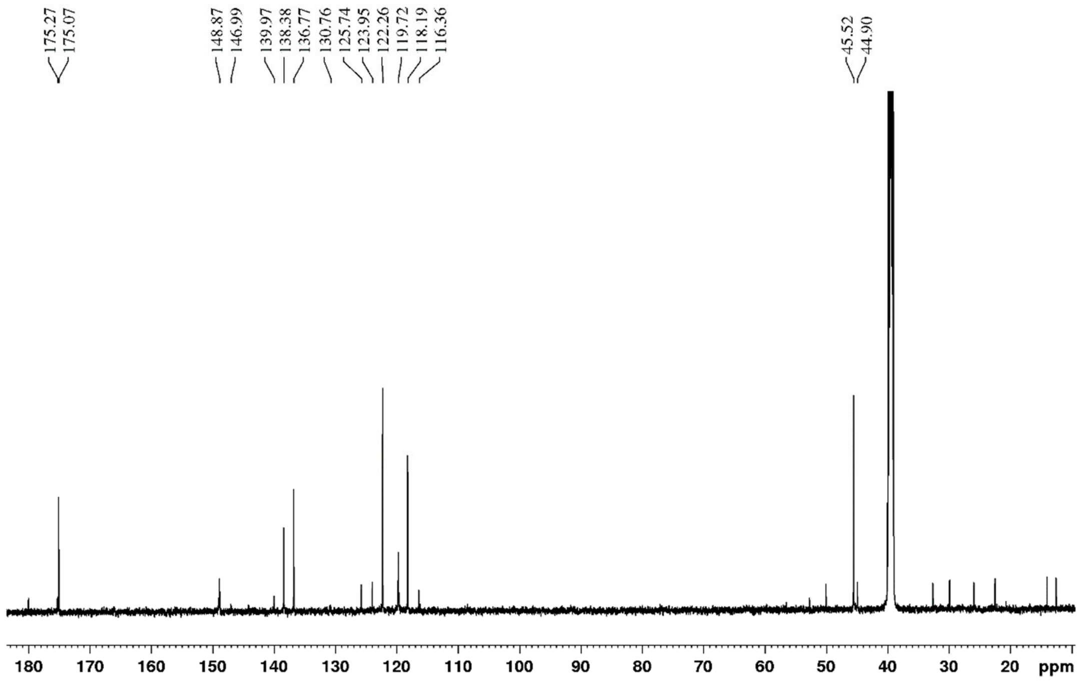

Figure S59. ^{13}C NMR spectrum of **Na2 DHPA-3'-S** and **Na2 DHPA-4'-S**. (150.93 MHz, DMSO-d_6)

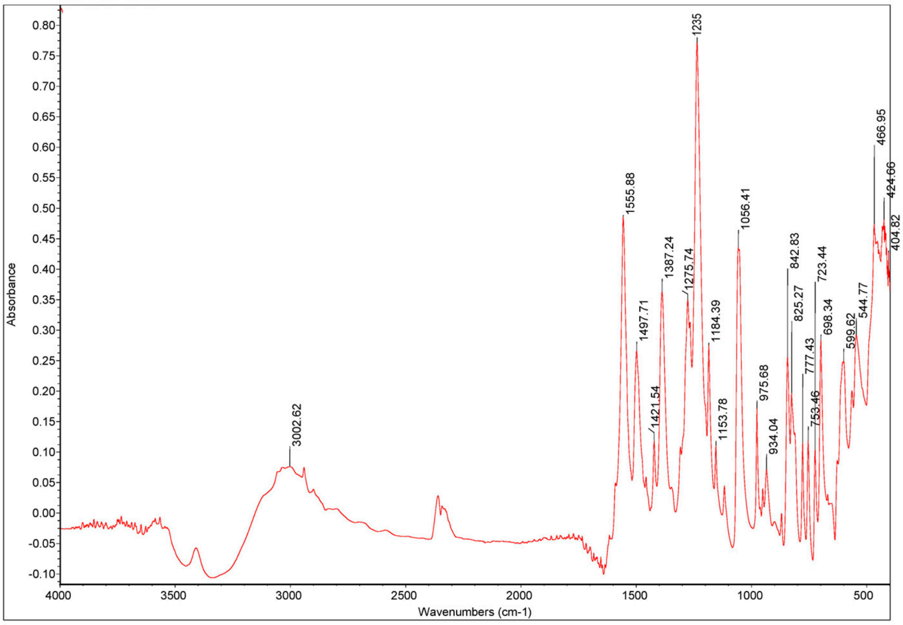

Figure S60. IR spectrum of compound **Na2 DHPA-3′-S** and **Na2 DHPA-4′-S**.

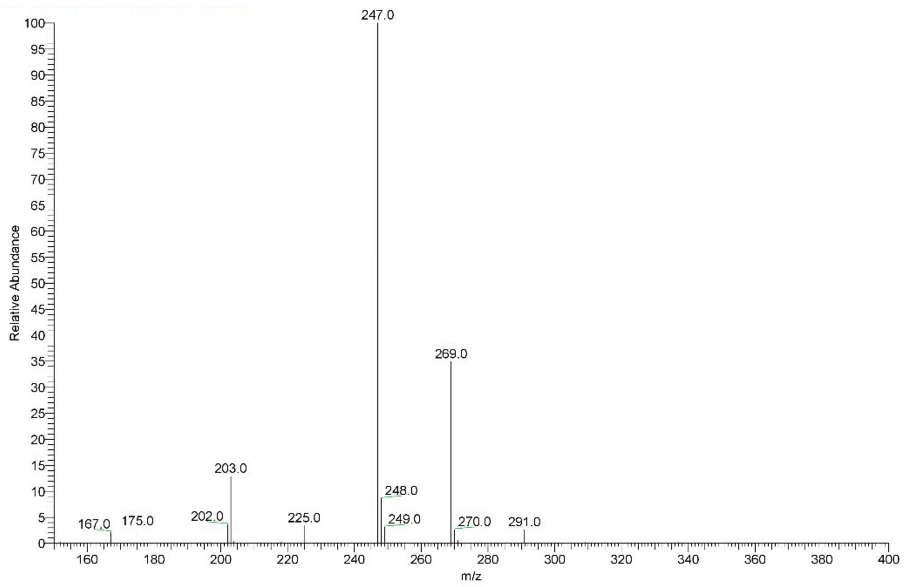

Figure S61. MS (ESI$^-$) spectrum of **Na$_2$ DHPA-S**.

([M – 2Na + H]$^-$, *m/z* 247.0; [M – Na]$^-$, *m/z* 269.0; [M – H]$^-$, *m/z* 291.0).

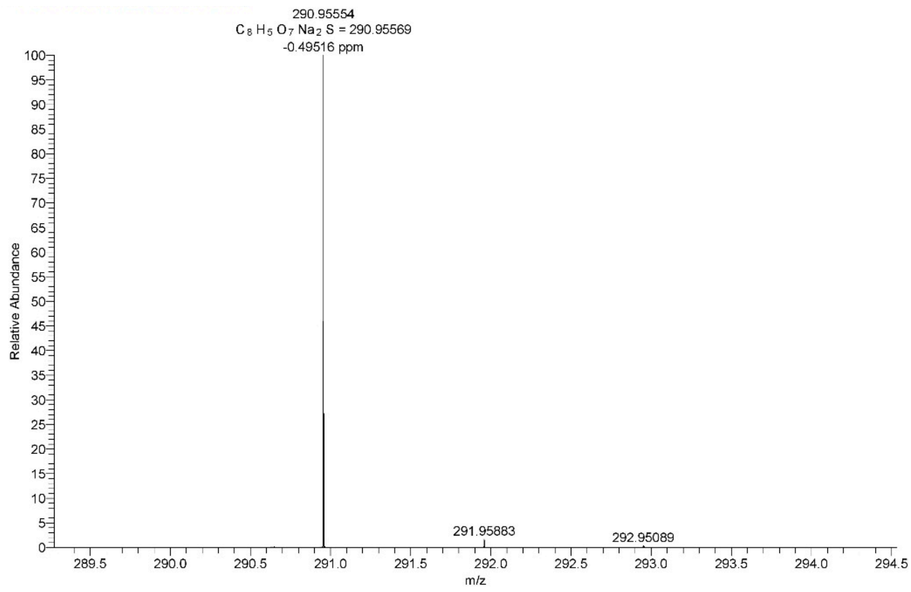

Figure S62. HRMS (ESI$^-$) spectrum of **Na$_2$ DHPA-S**.

Calculated (for $C_8H_5O_7Na_2S^-$) 290.95569, measured 290.95554 (-0.5 ppm).

Sodium phenylpropanoate sulfates (DHPP-S): sodium 3-(4-hydroxy-3-(sulfonatooxy)phenyl)propanoate (DHPP-3′-S) and sodium 2-(3-hydroxy-4-(sulfonatooxy)phenyl)propanoate (DHPP-4′-S)

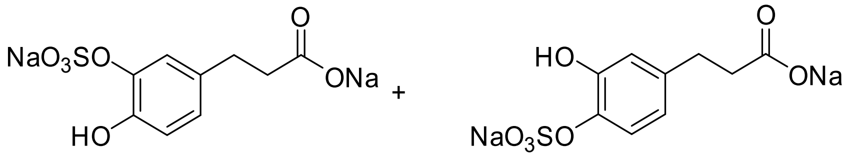

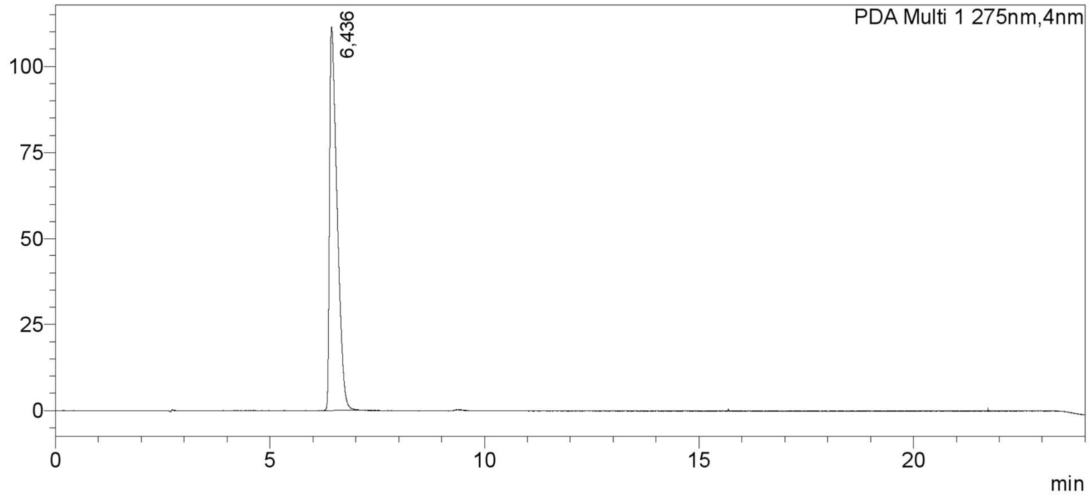

Figure S63. HPLC chromatogram of **Na_2 DHPP-3'-S** and **Na_2 DHPP-4'-S** (RT= 6.436 min, 99%)

Table S13. ^{1}H and ^{13}C NMR data of **Na_2 DHPP-3'-S** and **Na_2 DHPP-4'-S**. (600.23 MHz for ^{1}H, 150.93 MHz for ^{13}C, DMSO-d_6)

Na_2 DHPP-3'-S

Atom	δ_C	m.	δ_H	n_H	m.	J [Hz]	δ_C^{DHPP}	$\delta_C - \delta_C^{DHPP}$
1	177.01	S	-	0	-	-	173.76	3.25
2	39.77	T	2.152	2	m	-	35.61	4.16
3	31.6	T	2.651	2	m	-	29.68	1.92
1'	134.25	S	-	0	-	-	131.60	2.65
2'	122.86	D	6.925	1	d	2.2	115.55	7.31
3'	140.30	S	-	0	-	-	144.90	-4.60
4'	146.84	S	-	0	-	-	143.30	3.54
5'	116.76	D	6.686	1	d	8.2	115.36	1.40
6'	124.47	D	6.781	1	dd	8.2, 2.2	118.65	5.82

Na_2 DHPP-4'-S

Atom	δ_C	m.	δ_H	n_H	m.	J [Hz]	δ_C^{DHPP}	δ_C - δ_C^{DHPP}
**1**	176.90	S	-	0	-	-	173.76	3.14
**2**	39.88	T	2.168	2	m	-	35.61	4.27
**3**	31.99	T	2.660	2	m	-	29.68	2.31
**1'**	140.27	S	-	0	-	-	131.60	8.67
**2'**	117.01	D	6.670	1	d	2.1	115.55	1.46
**3'**	148.87	S	-	0	-	-	144.90	3.97
**4'**	138.43	S	-	0	-	-	143.30	-4.87
**5'**	122.82	D	6.920	1	d	8.1	115.36	7.46
**6'**	118.94	D	6.557	1	dd	8.1, 2.1	118.65	0.29

approximate molar ratio **Na_2 DHPP-3'-S**: **Na_2 DHPP-4'-S** = 14 : 86

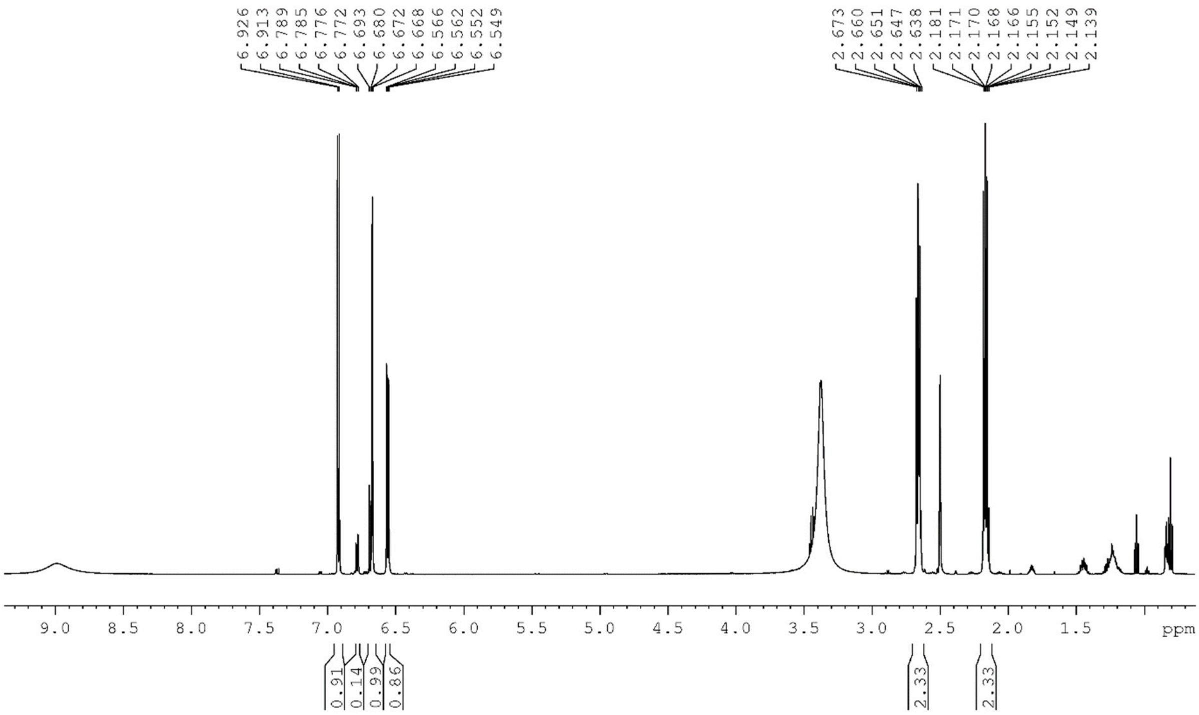

Figure S64. ^{1}H NMR spectrum of **Na_2 DHPP-3'-S and Na_2 DHPP-4'-S**.

(600.23 MHz, DMSO-d_6)

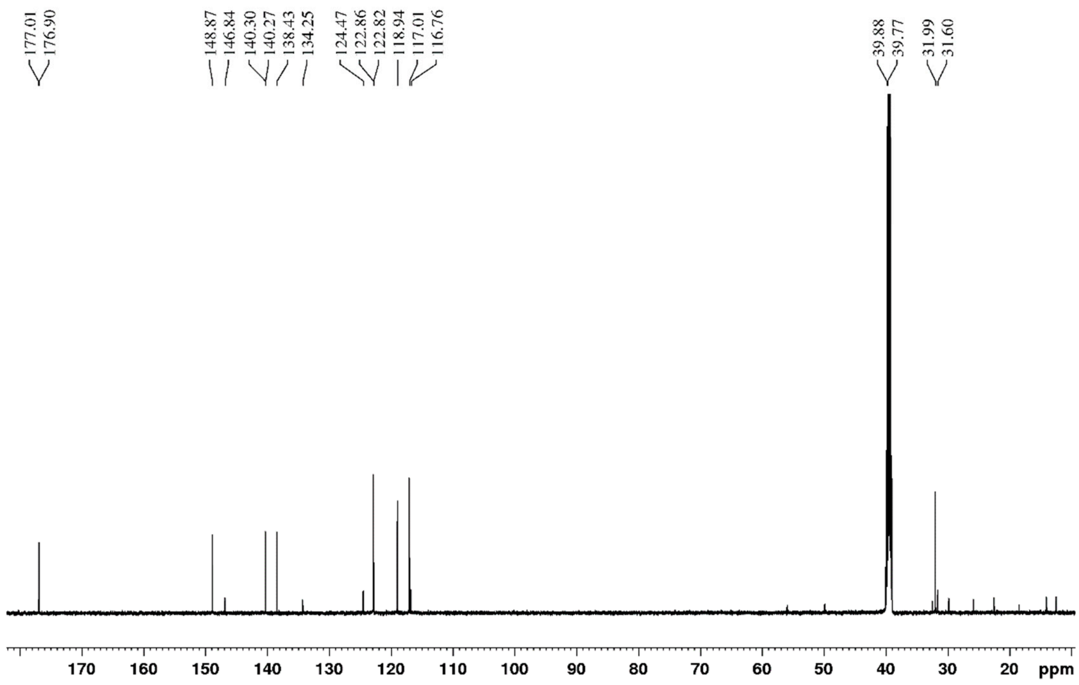

Figure S65. ^{13}C NMR spectrum of **Na_2 DHPP-3'-S and Na_2 DHPP-4'-S**.
(150.93 MHz, DMSO-d_6)

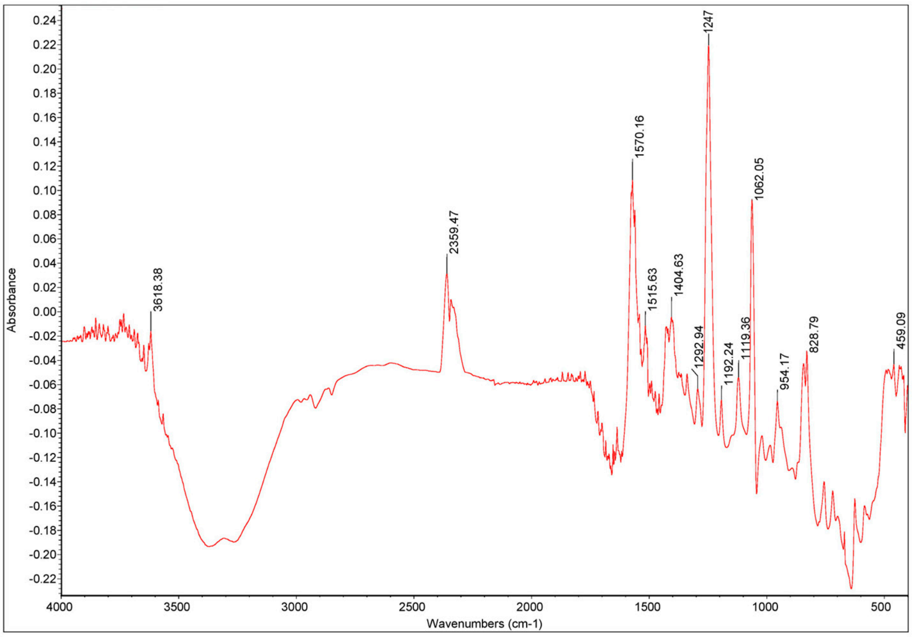

Figure S66. IR spectrum of compound **Na_2 DHPP-3′-S** and **Na_2 DHPP-4′-S**.

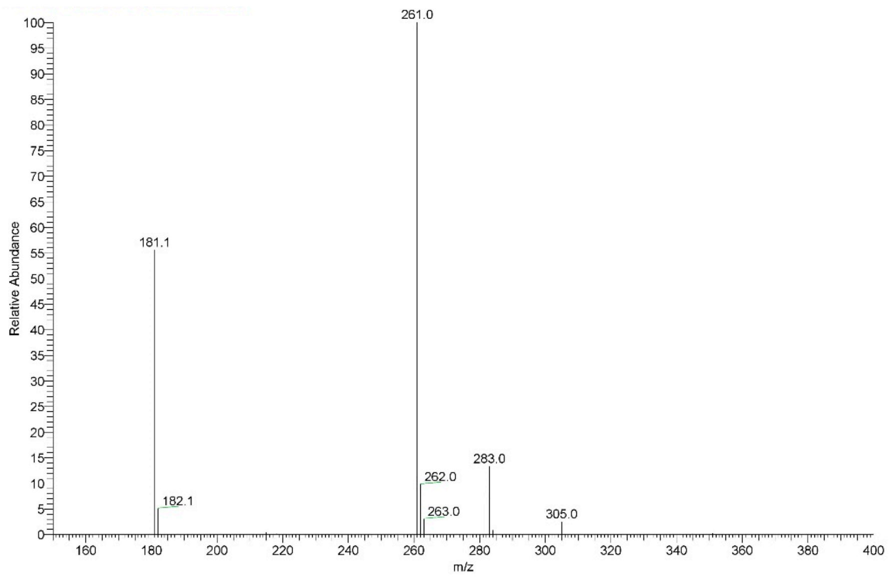

Figure S67. MS (ESI$^-$) spectrum of **Na_2 DHPP-S**.

([M – 2Na + H]$^-$, *m/z* 261.0; [M – Na]$^-$, *m/z* 283.0; [M – H]$^-$, *m/z* 305.0).

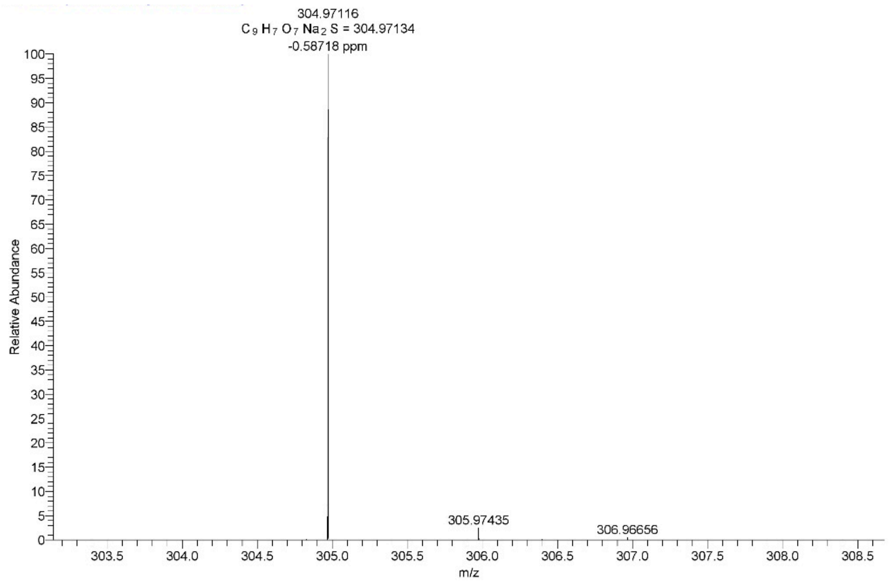

Figure S68. HRMS (ESI$^-$) spectrum of **Na_2 DHPP-S**.

Calculated (for $C_9H_7O_7Na_2S^-$) 304.97134, measured 304.97116 (-0.6 ppm).

(Trioxomethyl)methylammonium (2-hydroxyphenyl)acetate (2-HPA·Tris)

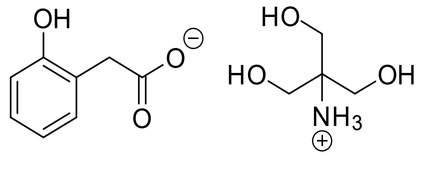

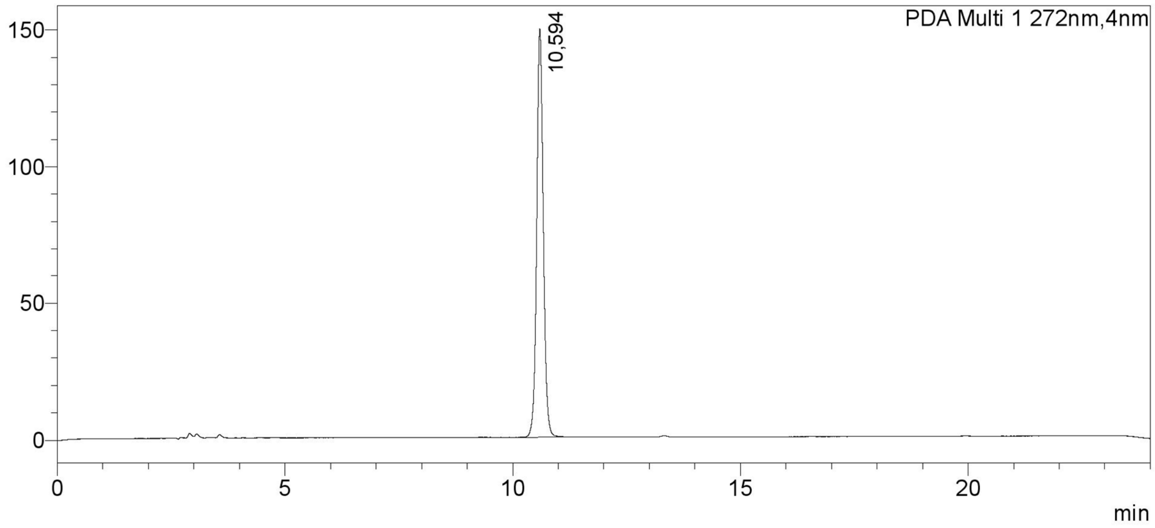

Figure S69. HPLC chromatogram of **2-HPA·Tris**.

(RT= 10.594 min, 99%)

Table S14. ^{1}H and ^{13}C NMR data for compound **2-HPA·Tris**.

(399.83 MHz for ^{1}H, 100.54 MHz for ^{13}C, DMSO-d_6)

**Atom**	δ_C	**m.**	δ_H	n_H	m.	**J [Hz]**	δ_C^{2HPA}	$\delta_C - \delta_C^{2HPA}$
**1**	175.48	S	-	0	-	-	172.63	2.85
**2**	43.53	T	3.301	2	s	-	35.23	8.30
**1'**	124.63	S	-	0	-	-	121.75	2.88
**2'**	157.74	S	-	0	-	-	155.26	2.48
**3'**	116.63	D	6.644	1	dd	8.0, 1.3	114.68	1.95
**4'**	127.00	D	6.972	1	ddd	8.0, 7.3, 1.8	127.67	-0.67
**5'**	117.84	D	6.612	1	ddd	7.4, 7.3, 1.3	118.58	-0.74
**6'**	130.02	D	6.919	1	dd	7.4, 1.8	130.92	-0.90

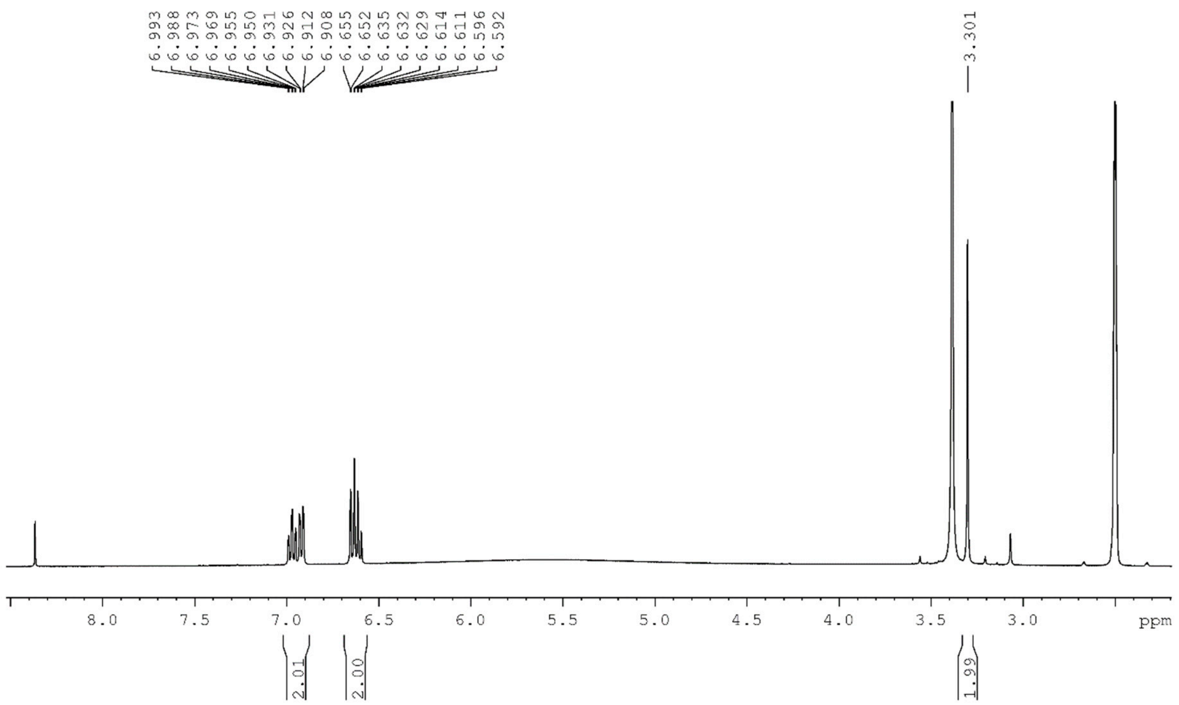

Figure S70. ^{1}H NMR spectrum of **2-HPA·Tris**.

(399.83 MHz, DMSO)

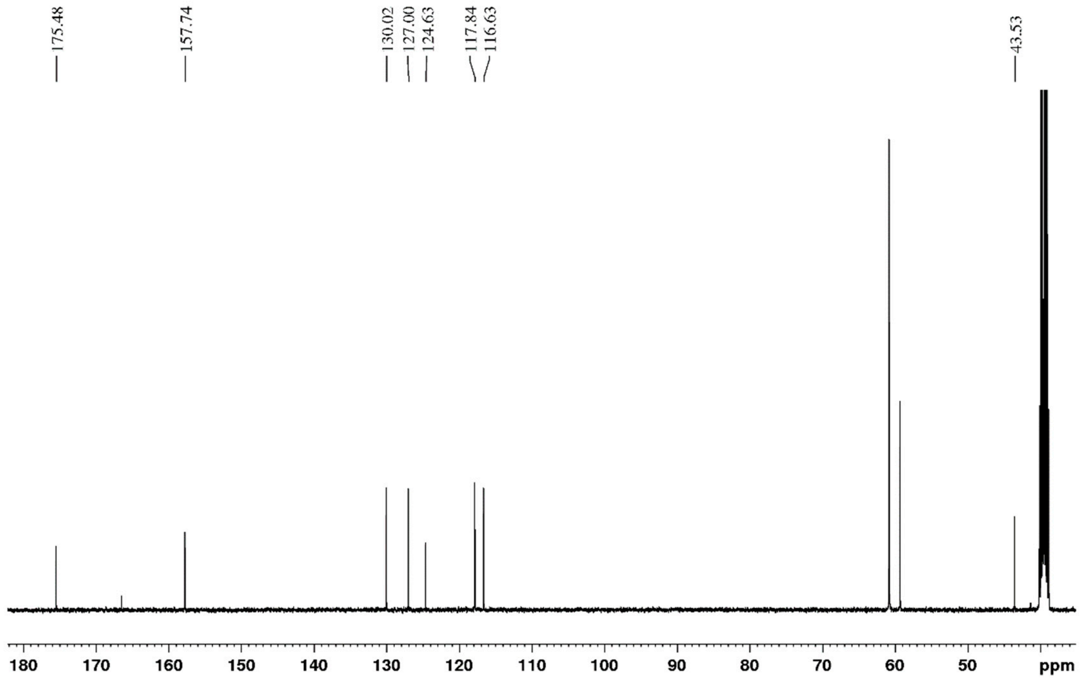

Figure S71. ^{13}C NMR spectrum of **2-HPA·Tris**.

(100.54 MHz, DMSO)

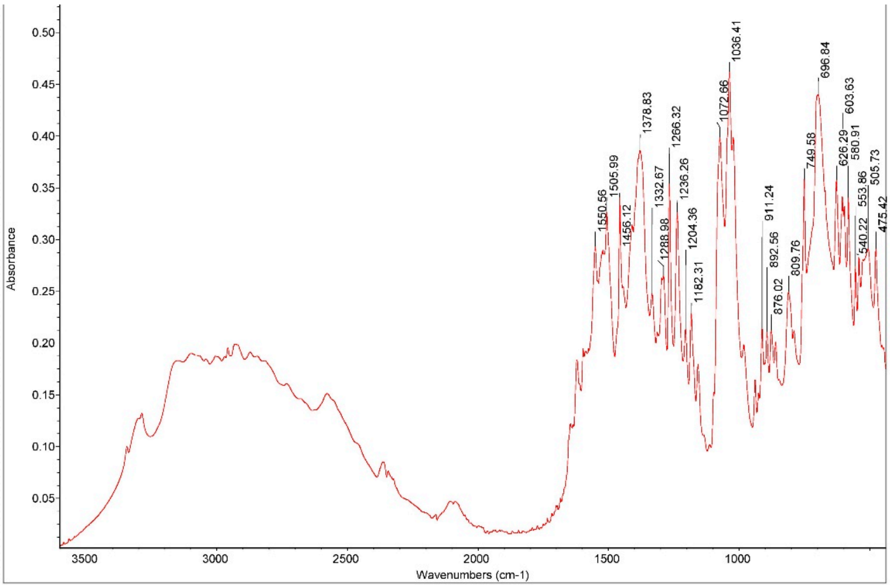

Figure S72. IR spectrum of **2-HPA·Tris**.

(Trioxomethyl)methylammonium (3-hydroxyphenyl)acetate (3-HPA·Tris)

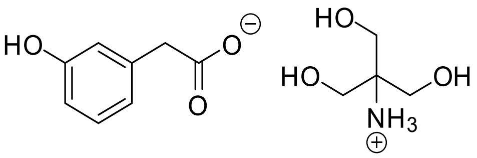

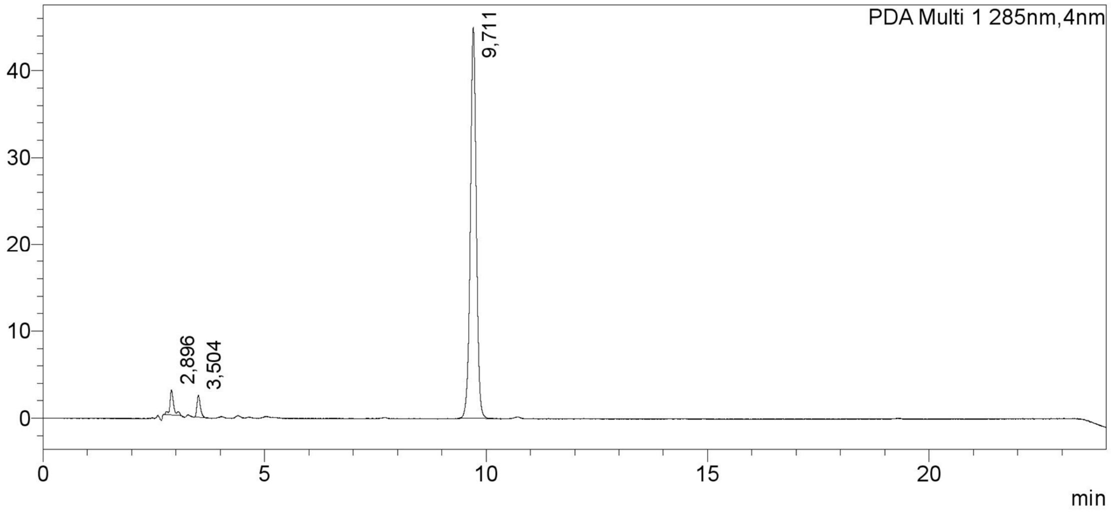

Figure S73. HPLC chromatogram of **3-HPA·Tris**.

(RT= 9.711 min, 93.2%)

Table S15. ^{1}H and ^{13}C NMR data for **3-HPA·Tris**.

(600.23 MHz for ^{1}H, 150.93 MHz for ^{13}C, DMSO-d_6)

Atom	δ_C	m.	δ_H	n_H	m.	J [Hz]	δ_C^{HPA}	δ_C - δ_C^{HPA}
**1**	174.60	S	-	0	-	-	172.52	2.08
**2**	43.89	T	3.251	2	s	-	40.72	3.17
**1'**	138.81	S	-	0	-	-	136.11	2.7
**2'**	116.21	D	6.663	1	dd	2.5, 1.6	116.12	0.09
**3'**	157.04	S	-	0	-	-	157.16	-0.12
**4'**	112.64	D	6.555	1	ddd	8.1, 2.5, 1.0	113.51	-0.87
**5'**	128.55	D	7.006	1	dd	8.1, 7.5	129.08	-0.53
**6'**	119.76	D	6.612	1	ddd	7.5, 1.6, 1.0	119.83	-0.07

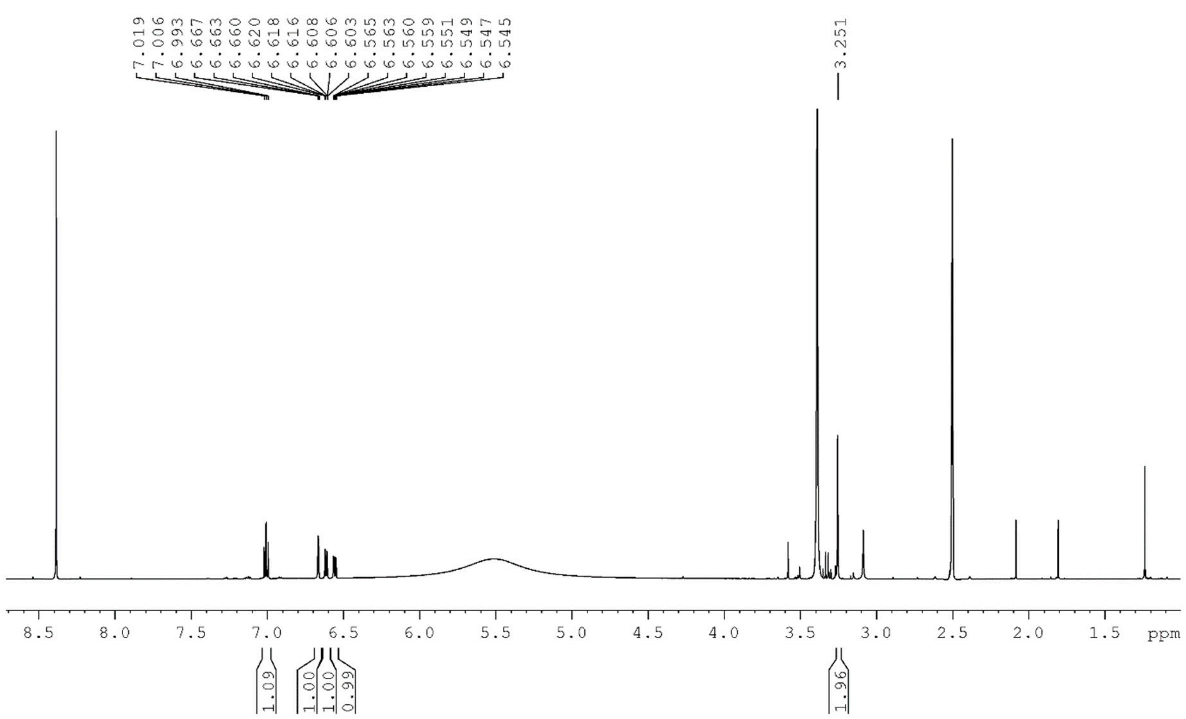

Figure S74. ^{1}H NMR spectrum of **3-HPA·Tris**.

(600.23 MHz, DMSO-d_6)

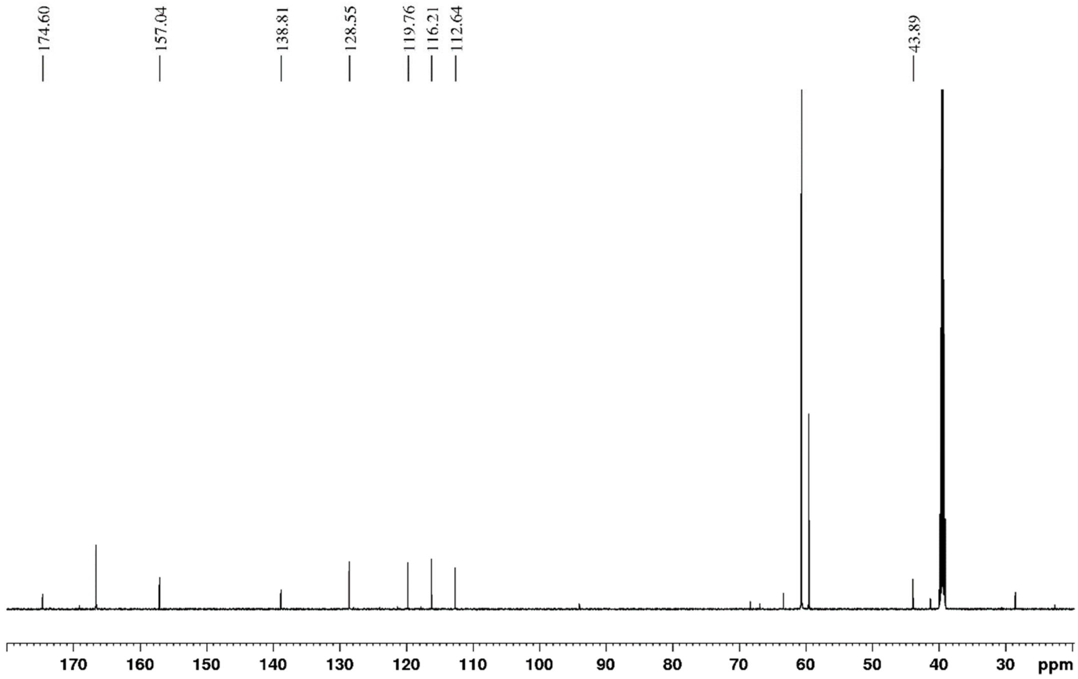

Figure S75. ^{13}C NMR spectrum of **3-HPA·Tris**.

(150.93 MHz for ^{13}C, DMSO-d_6)

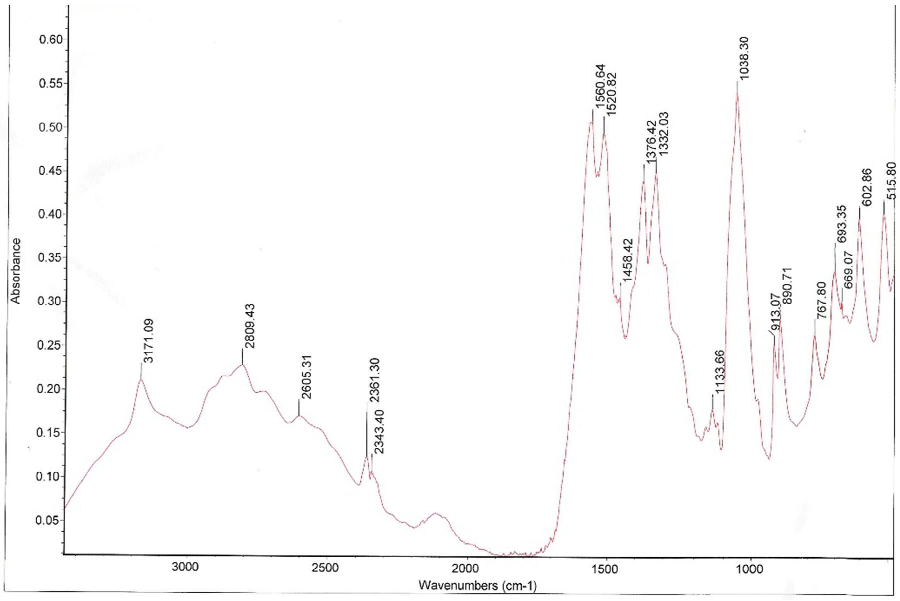

Figure S76. IR spectrum of **3-HPA·Tris**.

(Trioxomethyl)methylammonium (4-hydroxyphenyl)acetate (4-HPA·Tris)

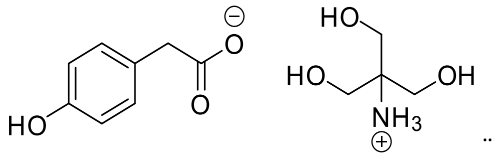

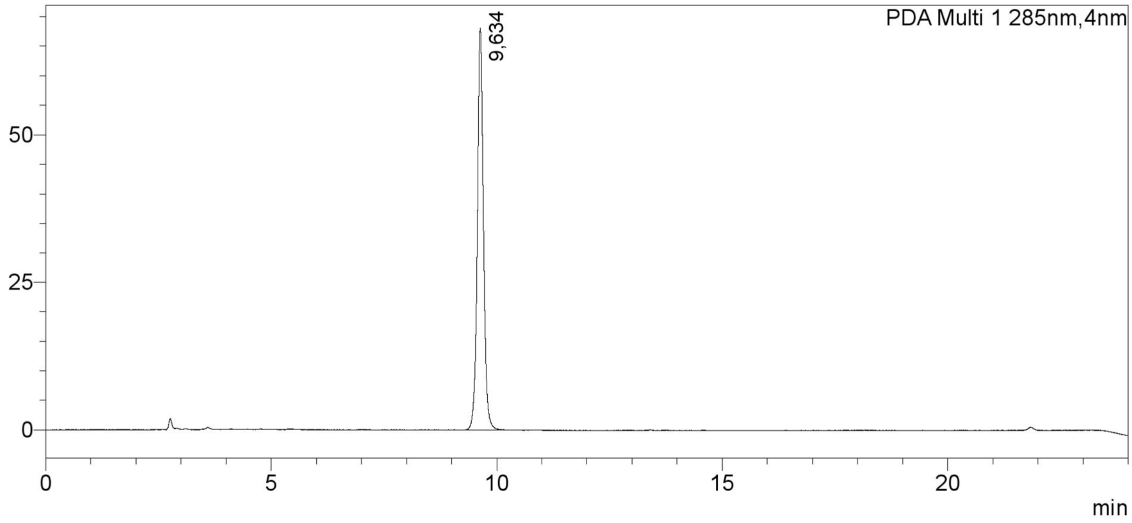

Figure S77. HPLC chromatogram of **4-HPA·Tris**.

(RT= 9.634 min, 99%)

Table S16. ^{1}H and ^{13}C NMR data for **4-HPA·Tris**.
(399.83 MHz for ^{1}H, 100.54 MHz for ^{13}C, DMSO-d_6)

Atom	δ_C	m.	δ_H	n_H	m.	δ_C^{2HPA}	δ_C - δ_C^{2HPA}
**1**	174.55	S	-	0	-	172.99	1.56
**2**	42.05	T	3.268	2	s	39.79	2.26
**1'**	127.02	S	-	0	-	125.03	1.99
**2'**	129.97	D	7.003	2	m	130.13	-0.16
**3'**	114.67	D	6.647	2	m	114.92	-0.25
**4'**	155.51	S	-	0	-	155.95	-0.44

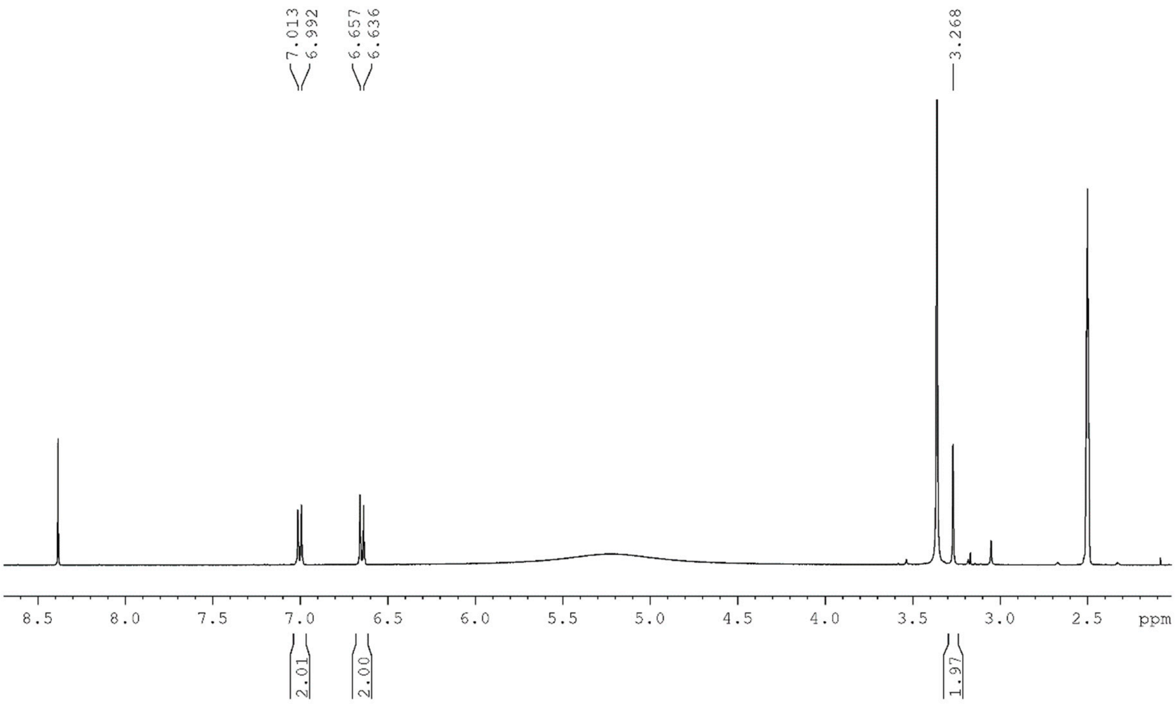

Figure S78. ^{1}H NMR spectrum of **4-HPA·Tris**.
(399.83 MHz, DMSO-d_6)

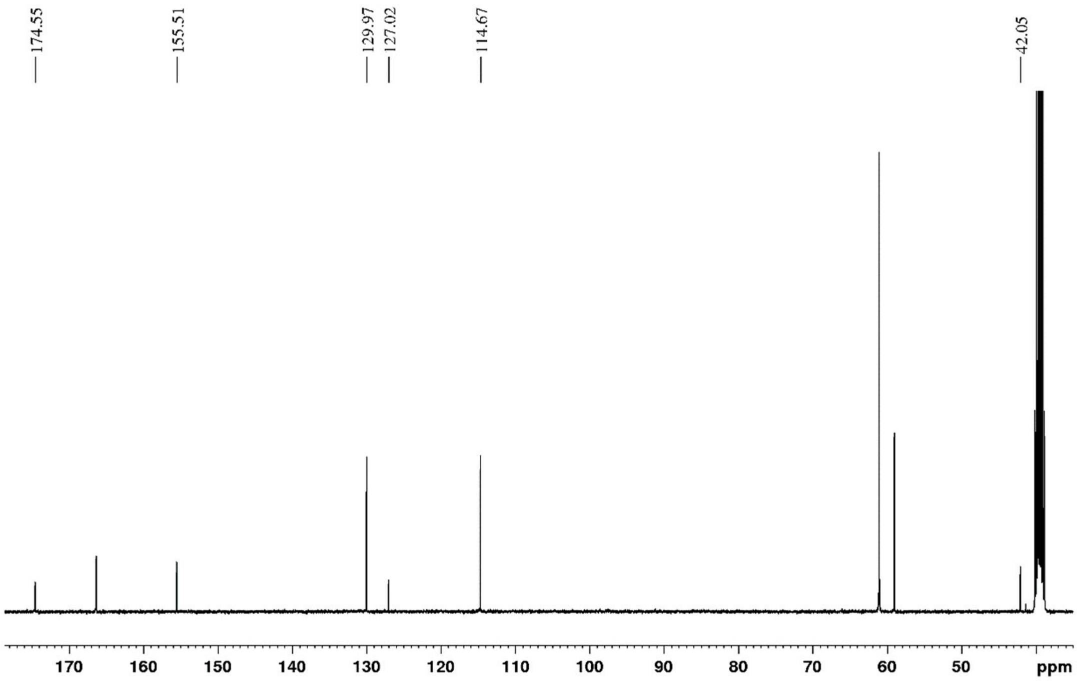

Figure S79. ^{13}C NMR spectrum of **4-HPA·Tris**.
(100.54 MHz, DMSO-d_6)

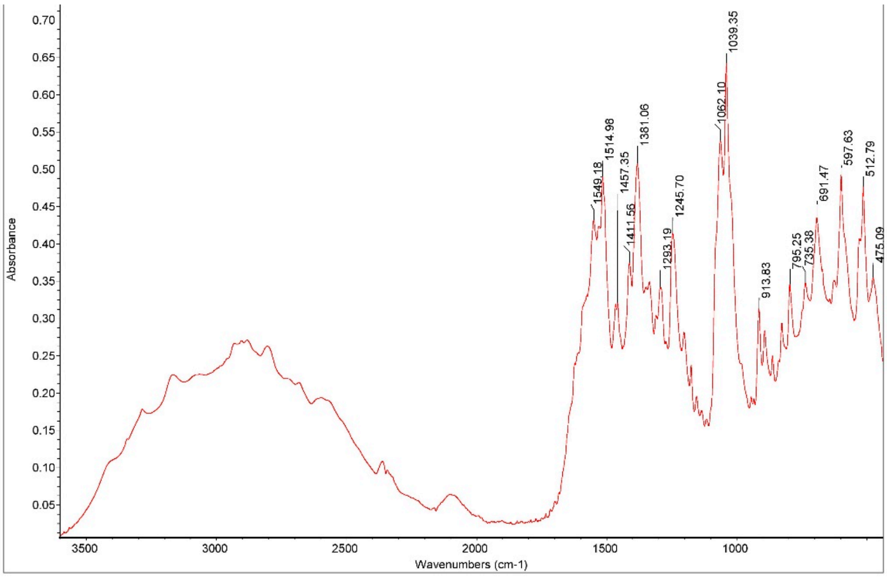

Figure S80. IR spectrum of **4-HPA·Tris**.

(Trioxomethyl)methylammonium (4-hydroxyphenyl)propanoate (4-HPP·Tris)

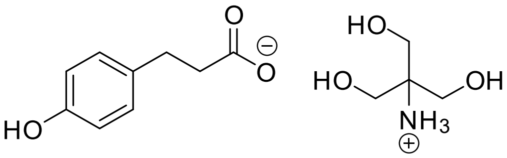

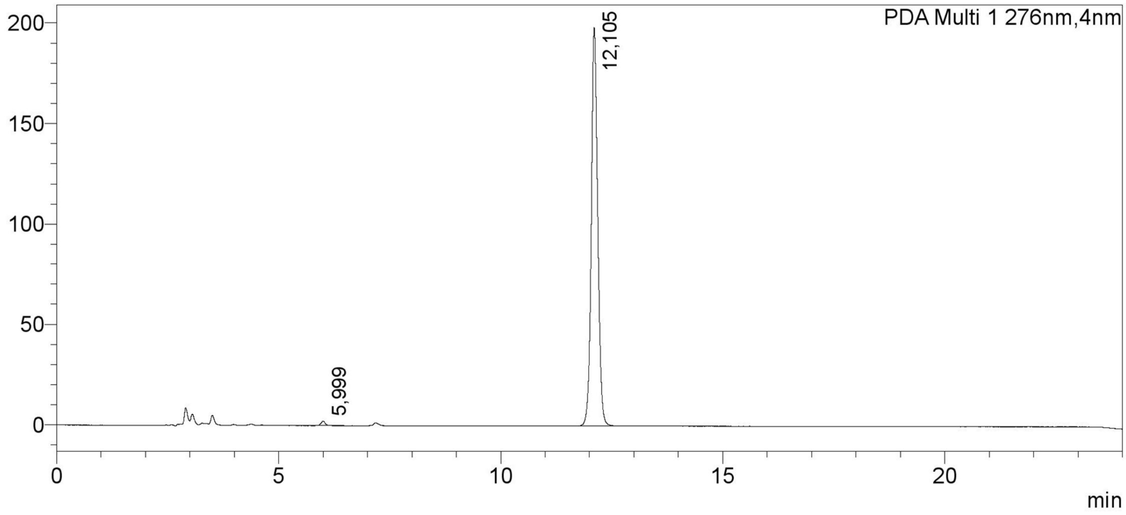

Figure S81. HPLC chromatogram of **4-HPA·Tris**.

(RT= 12.105 min, 99%)

Table S17. ^{1}H and ^{13}C NMR data for **4-HPP·Tris**.

(600.23 MHz for ^{1}H, 150.93 MHz for ^{13}C, DMSO-d_6)

**Atom**	δ_C	**m.**	δ_H	n_H	m.	***J* [Hz]**	δ_C^{HPP}	$\delta_C - \delta_C^{HPP}$
**1**	175.46	S	-	0	-	-	173.76	1.7
**2**	37.39	T	2.289	2	m	-	35.64	1.75
**3**	30.50	T	2.657	2	m	-	29.50	1
**1'**	131.80	S	-	0	-	-	130.84	0.96
**2'6'**	128.84	D	6.966	2	m	Σ*J* = 8.6	128.97	-0.13
**3'5'**	114.91	D	6.640	2	m	Σ*J* = 8.6	114.97	-0.06
**4'**	155.34	S	-	1	-	-	155.43	-0.09

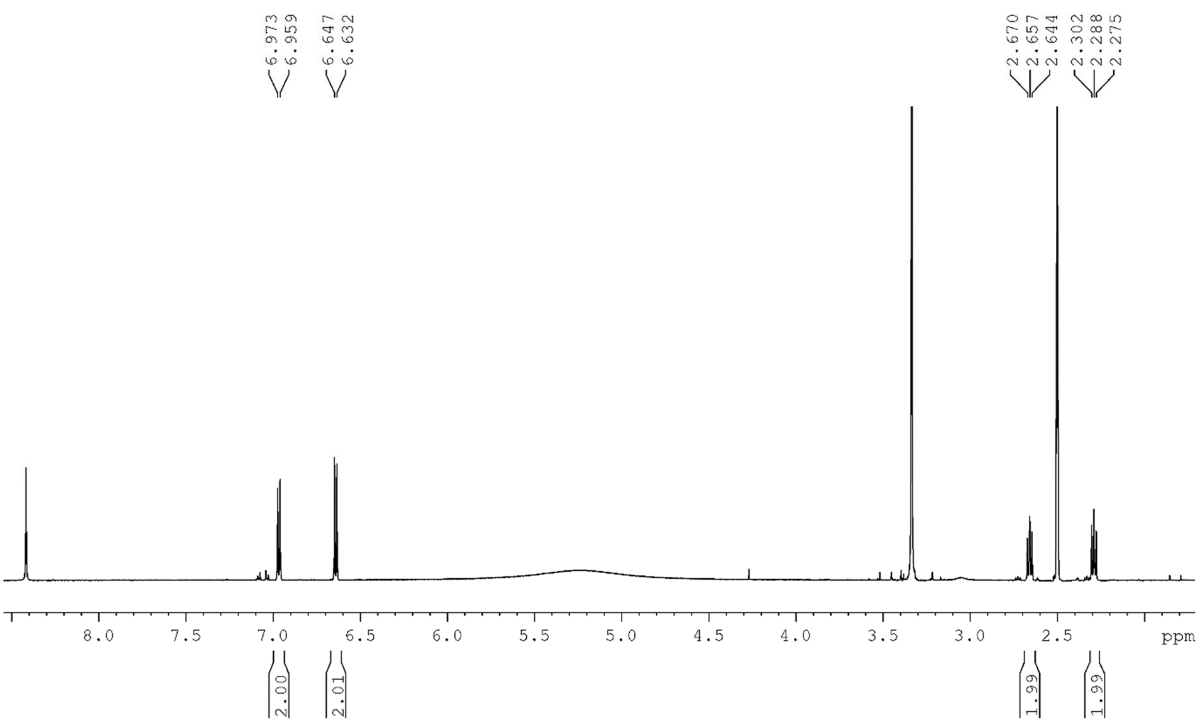

Figure S82. ^{1}H NMR spectrum of **4-HPP·Tris**

(600.23 MHz, DMSO-d_6)

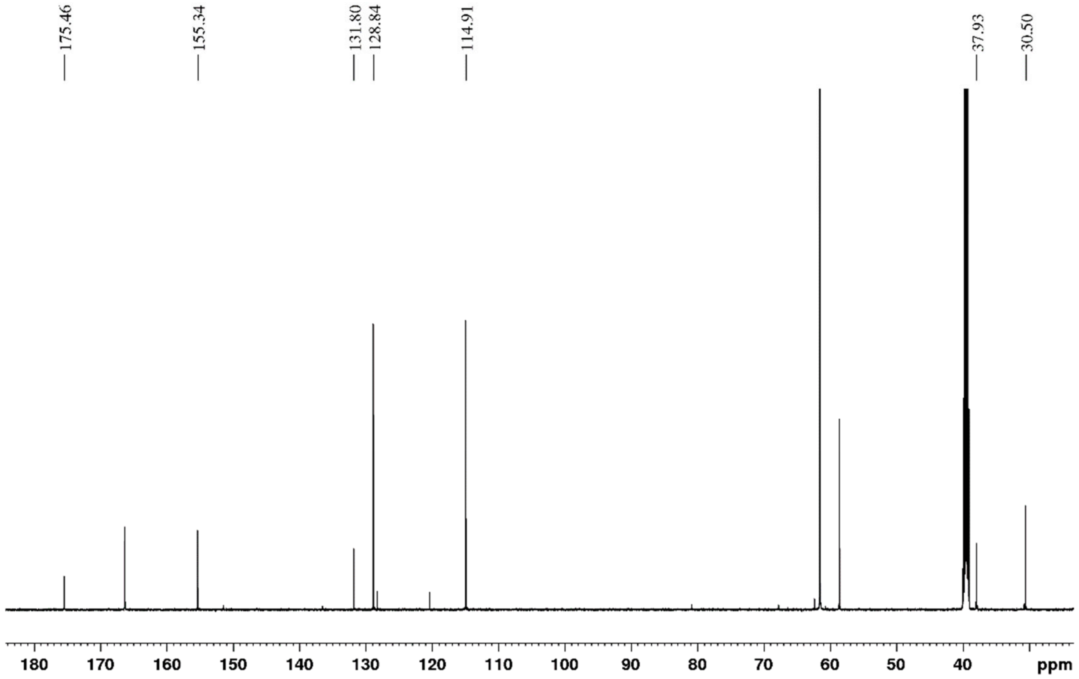

Figure S83. ^{13}C NMR spectrum of **4-HPP·Tris**.

(150.93 MHz, DMSO-d_6)

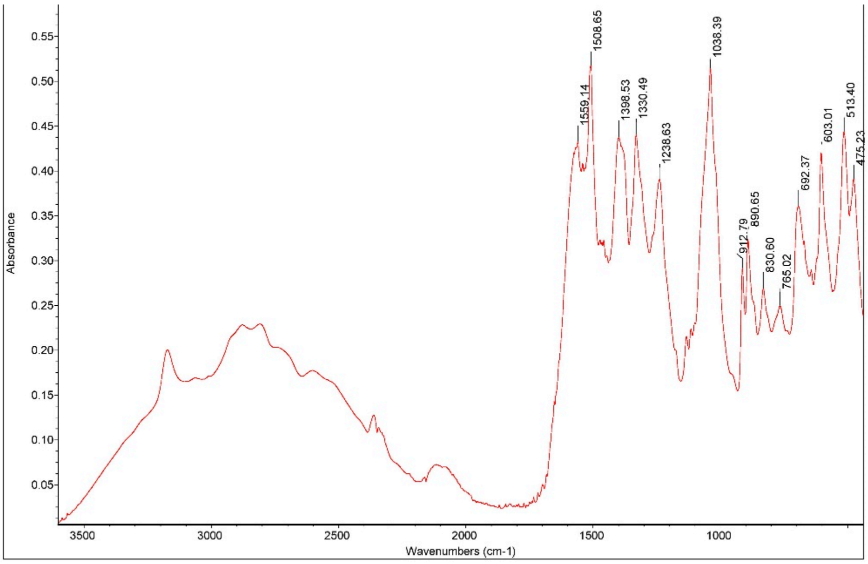

Figure S84. IR spectrum of **4-HPP·Tris**.
